# Descriptive comparison of admission characteristics between pandemic waves and multivariable analysis of the association of the Alpha variant (B.1.1.7 lineage) of SARS-CoV-2 with disease severity in inner London

**DOI:** 10.1136/bmjopen-2021-055474

**Published:** 2022-02-08

**Authors:** Luke B Snell, Wenjuan Wang, Adela Alcolea-Medina, Themoula Charalampous, Rahul Batra, Leonardo de Jongh, Finola Higgins, Gaia Nebbia, COG-UK HOCI Investigators, Yanzhong Wang, Jonathan Edgeworth, Vasa Curcin

**Affiliations:** 1 Centre for Clinical Infection & Diagnostics Research, King’s College London, London, UK; 2 Department of Infection, Guy’s and St Thomas’ NHS Foundation Trust, London, UK; 3 Department of Population Health Sciences, King’s College London, London, UK; 4 Infection Sciences, Viapath, London, UK; 5 NIHR Biomedical Research Centre, Guy’s and St. Thomas’ NHS Foundation Trust, London, UK

**Keywords:** virology, epidemiology, epidemiology, public health

## Abstract

**Background:**

The Alpha variant (B.1.1.7 lineage) of SARS-CoV-2 emerged and became the dominant circulating variant in the UK in late 2020. Current literature is unclear on whether the Alpha variant is associated with increased severity. We linked clinical data with viral genome sequence data to compare admitted cases between SARS-CoV-2 waves in London and to investigate the association between the Alpha variant and the severity of disease.

**Methods:**

Clinical, demographic, laboratory and viral sequence data from electronic health record systems were collected for all cases with a positive SARS-CoV-2 RNA test between 13 March 2020 and 17 February 2021 in a multisite London healthcare institution. Multivariate analysis using logistic regression assessed risk factors for severity as defined by hypoxia at admission.

**Results:**

There were 5810 SARS-CoV-2 RNA-positive cases of which 2341 were admitted (838 in wave 1 and 1503 in wave 2). Both waves had a temporally aligned rise in nosocomial cases (96 in wave 1 and 137 in wave 2). The Alpha variant was first identified on 15 November 2020 and increased rapidly to comprise 400/472 (85%) of sequenced isolates from admitted cases in wave 2. A multivariate analysis identified risk factors for severity on admission, such as age (OR 1.02, 95% CI 1.01 to 1.03, for every year older; p<0.001), obesity (OR 1.70, 95% CI 1.28 to 2.26; p<0.001) and infection with the Alpha variant (OR 1.68, 95% CI 1.26 to 2.24; p<0.001).

**Conclusions:**

Our analysis is the first in hospitalised cohorts to show increased severity of disease associated with the Alpha variant. The number of nosocomial cases was similar in both waves despite the introduction of many infection control interventions before wave 2.

**Strengths and limitations of this study**

- Published evidence on whether the Alpha variant of SARS-CoV-2 causes more severe disease (COVID-19) is mixed.
- Our study benefits from a long study window, including patients since the beginning of the SARS-CoV-2 pandemic.
- Our outcome measure for severity, hypoxia on admission, reflects the natural history of disease prior to medical intervention and hospital treatment.
- Our analysis adjusts for comorbidities, a feature missing from many of the population-level studies currently published.

## Background

SARS-CoV-2 infection has led to the death of over 4 million individuals worldwide since its emergence in China during December 2019, with over 120 000 deaths reported in the UK as of July 2021. In London, the estimated incidence of new cases in the first wave peaked around 23 March 2020 at 2.2%^1^ and then rapidly declined following non-pharmacological interventions. Hospital admissions peaked about 1 week later,^2^ reflecting the median period of symptoms before hospital presentation. A ‘second wave’ of infections started in London around the beginning of October 2020.^3^

Genome sequencing identified the Alpha variant (the B.1.1.7 lineage) around the South East England, which spread rapidly as part of the emerging second wave.^4^ This occurred prior to widespread vaccination, with only 25% of the adult population receiving the first dose by mid-February 2021.^5^ The Alpha variant has been associated with increased transmissibility in community studies,^6^ ^7^ and community studies associate the variant with increased mortality.^8^ ^9^ However, published studies in hospitalised patients suggested no increase in need for ventilation or mortality.^10^

Changes in transmissibility and severity have the potential to affect the burden on healthcare systems, and modify the characteristics of cases presenting to hospitals, including the demographics, comorbidities and severity of disease associated with SARS-CoV-2 infection.

### Objectives

We linked clinical datasets with local SARS-CoV-2 variant analysis to compare admission characteristics of hospitalised cases during the two waves of infection and to look at the association of the Alpha variant with severity of disease at presentation to the hospital.

## Methods

### Setting

Guy’s and St Thomas’ NHS Foundation Trust (GSTT) is a multisite healthcare institution providing general and emergency services predominantly to the South London boroughs of Lambeth and Southwark. An acute-admitting site (St Thomas’ Hospital) has an adult emergency department, with a large critical care service including one of the UK’s eight nationally commissioned extracorporeal membrane oxygenation (ECMO) centres for severe respiratory failure. A second site (Guy’s Hospital) provides more inpatient services such as elective surgery, cancer care and other specialist services. A paediatric hospital (Evelina London) acts as a general and specialised referral centre. Several satellite sites for specialist services like dialysis, rehabilitation and long-term care are also part of the institution. GSTT receives patients from regional hospitals predominantly critical care through ‘mutual aid' schemes.

### SARS-CoV-2 laboratory testing

Our laboratory began testing on 13 March 2020 with initial capacity for around 150 PCR tests per day, before increasing to around 500 tests per day in late April during wave 1 and up to 1000 tests per day during wave 2 (online supplemental figure 1).

10.1136/bmjopen-2021-055474.supp1 Supplementary data

Testing commenced during the first wave on 13 March 2020 was limited to cases requiring admission or inpatients who had symptoms of fever or cough, as per national recommendation; guidance suggested cases which did not require admission should not be tested. For wave 2, all cases admitted to the hospital were screened and underwent universal interval screening at varying time points. Staff testing for symptomatic healthcare workers (HCWs) was also introduced towards the end of wave 1. Comparative analysis was therefore restricted to SARS-CoV-2 RNA-positive cases requiring admission. Cases without laboratory confirmation of SARS-CoV-2 infection were not included.

Assays used for the detection of SARS-CoV-2 RNA include PCR testing using Aus Diagnostics or by the Hologic Aptima SARS-CoV-2 Assay. Nucleic acid was first extracted using the QIAGEN QIAsymphony SP system and a QIAsymphony DSP Virus/Pathogen Mini Kit (catalogue number 937036) with the off-board lysis protocol.

### Definitions and participants

Cases were identified by the first positive SARS-CoV-2 RNA test. Cases were placed in mutually exclusive categories with the following definitions: (1) outpatients; (2) testing through occupational health; (3) emergency department (ED) attenders not subsequently admitted within 14 days; (4) patients admitted within 14 days of a positive test; (5) nosocomial cases, defined based on European Centre for Disease Prevention and Control (ECDC) definitions, as those having a first positive test on day 8 or later after admission to the hospital where COVID-19 was not suspected on admission;^11^ and (6) interhospital transfers.

For the purpose of comparison, only the inpatient group admitted within 14 days following a positive test was taken forward for onward comparison. This methodology of only including admissions was adopted to prevent increased testing during the pandemic affecting case ascertainment and biasing severity of cases. This is evidenced in online supplemental figure 1, with tests increasing steadily from 100 per day to more than 1000 per day. Additionally, in wave 2, more interhospital transfers of severe cases requiring ECMO were received, mostly several days after admission. This category of patients was therefore excluded from analysis to prevent biasing towards severe disease.

A composite data point for ‘hypoxia’ was created, equivalent to WHO ordinal scale of ≥4,^12^ with cases taken to be hypoxic if on admission they had oxygen saturations of <94%, if they were recorded as requiring supplemental oxygen or if the fraction of inspired oxygen was recorded as being greater than 0.21.

### Determination of SARS-CoV-2 lineage

Whole-genome sequencing of residual samples from SARS-CoV-2 cases was performed using GridION (Oxford Nanopore Technology), using V.3 of the ARTIC protocol^13^ and bioinformatics pipeline.^14^ Samples were selected for sequencing if the corrected CT value was 33 or below, or the Hologic Aptima assay was above 1000 Relative Light Units (RLU). During the first wave, sequencing occurred between 1–31 March, while sequencing in the second wave restarted in November 2020–March 2021. Variants were called using updated versions of pangolin V.2.0.^15^ We considered all cases in wave 1 to be non-Alpha variants, as our wave 1 cut-off of 25 July 2020 was 6 weeks prior to first identified cases of the Alpha variant in the UK^16^ and before the Alpha variant was first identified in our population in November 2020.

### Data sources, extraction and integration

Clinical, laboratory and demographic data for all cases with a laboratory-reported SARS-CoV-2 PCR RNA test on nose and throat swabs or lower respiratory tract specimens were extracted from hospital electronic health record data sources using records closest to the test date. Data were linked to the Index of Multiple Deprivation. Age, sex and ethnicity were extracted from the Electronic Patient Record (EPR). Self-reported Office for National Statistics (ONS) ethnic categories were stratified into white (British, Irish, Gypsy and white–other) or non-white (black (African, Caribbean and black–other) or Asian (Bangladeshi, Chinese, Indian, Pakistan and Asian–other) and mixed/other). Numbers for which data were missing are listed by each variable. Comorbidities and medication history were extracted from the EPR and e-noting using natural language processing (NLP). If a comorbidity was not recorded, it was assumed not to be present. Cases were characterised as having/not having a medical history of hypertension, cardiovascular disease (stroke, transient ischaemic attack, atrial fibrillation, congestive heart failure, ischaemic heart disease, peripheral artery disease or atherosclerotic disease), diabetes mellitus, chronic kidney disease, chronic respiratory disease (chronic obstructive pulmonary disease, asthma, bronchiectasis or pulmonary fibrosis) and neoplastic disease (solid tumours, haematological neoplasias or metastatic disease). Obesity was defined as either obesity present in the notes or recorded body mass index ≥30 kg/m^2^. Medicines data were extracted using both structured queries and NLP tools with medical and drug dictionaries. Additionally, checks on free text data were performed by a cardiovascular clinician to ensure the information was accurate.

Analysis was carried out on the secure Rosalind high-performance computer infrastructure^17^ running Jupyter Notebook V.6.0.3, R V.3.6.3 and Python V.3.7.6.

### Statistical analysis and outcome measures

Descriptive statistics were summarised with mean and standard deviation for continuous variables if the distribution is normal, and the median and IQR if the distribution are non-normal. Count and percentages were used for categorical variables. For the comparisons of variables for wave 1 versus wave 2 variables, Alpha variant versus non-Alpha variants, as well as sequenced patients versus non-sequenced patients in wave 2, Kruskal-Walllis test was used for continuous variables and χ^2^ test for categorical variables with significance level of p<0.05. Multivariate analysis was performed using logistic regression to assess the odds ratios of different risk factors (including age, sex, ethnicity (white, non-white and unknown), variant status (Alpha or non-Alpha), and cardiovascular disease, hypertension, diabetes, chronic respiratory disease, cancer, kidney disease, HIV, transplant and frailty) for hypoxia on admission as the binary outcome indicating severity at admission. Variables to be included in the multivariate analysis were chosen by literature review and expert opinion (see online supplemental material). Cases with missing data points were dropped from analysis.

10.1136/bmjopen-2021-055474.supp2 Supplementary data

## Results

### General epidemiology and results of viral genome sequencing

**Figure 1:**
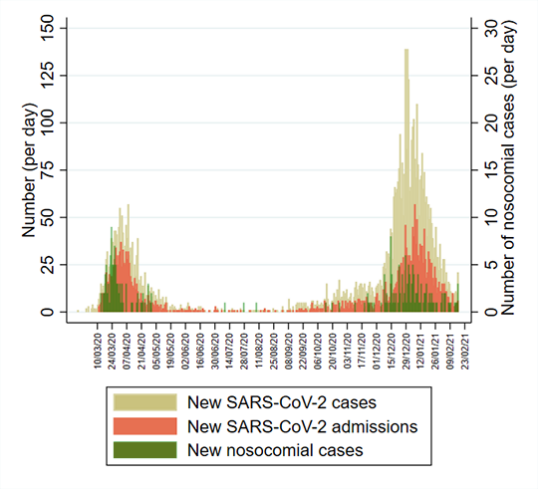
Distribution of laboratory-confirmed SARS-CoV-2 cases over time. Daily incidence of new cases (beige), newly admitted cases (orange) and nosocomial acquisitions (green) over time.

Figure 1 shows the incidence of SARS-CoV-2 cases, SARS-CoV-2 admissions and nosocomial cases since 13 March 2020. In total, 5810 individuals had a positive SARS-CoV-2 PCR test up until the data extraction date of 17 February 2021. Two ‘waves’ are evident with 25 July taken as a separation date between waves, at which point a minimum of 12 wave 1 cases remained in the hospital. Wave 1 comprised 1528 cases (26.3%) from when laboratory testing commenced on 13 March to peak rapidly between 1 and 8 April 2020 with 57 new cases per day, before falling to a baseline by 12 May 2020. Ninety-one per cent (1391/1528) of all cases in wave 1 occurred during these 60 days. Wave 2 comprised 4282 cases (73.7%), with incidence first increasing gradually from the beginning of October. There was then a period of rapidly escalating incidence from about 10 December, peaking on 28 December 2020 when 139 cases per day were diagnosed. Of 4282 wave 2 cases, 3446 (80%) were detected during a comparable 60-day period between 10 December 2020 and 8 February 2021. In both waves, nosocomial cases peaked early, increasing along with admissions but then fell while the number of community admissions continued at peak levels.

**Figure 2:**
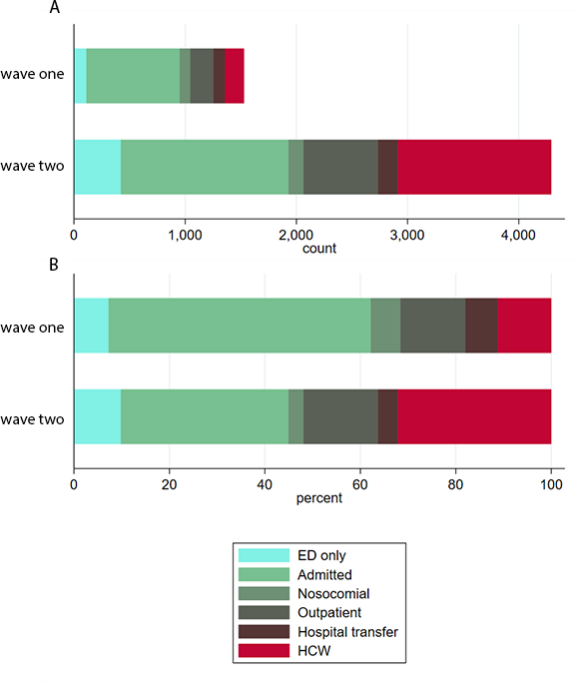
(A) Absolute number of cases within the different hospital cohorts during wave 1 (upper) and wave 2 (lower). (B) Proportion of cases within the different hospital cohorts during wave 1 (upper) and wave 2 (lower). ED, emergency department; HCW, healthcare worker.

Individuals with a positive test were placed into six categories (figure 2). The 5810 SARS-CoV-2 cases were categorised as follows: inpatients admitted within 14 days of a positive test (n=2341), HCWs (n=1549), outpatients (n=874), ED attenders not subsequently admitted (n=532), interhospital transfers (n=281) and nosocomial cases (n=233). Some observed differences between waves 1 and 2 reflected the increased availability of testing particularly for outpatients (208, 13.6%, vs 666, 15.6%), people sent home from ED (111, 7.3%, vs 421, 9.8%) and HCWs (171, 11.2%, vs 1378, 32.2%). There were also more interhospital transfers of known COVID-19 cases in wave 2 (177, 4.1%, vs 104, 6.8%, in wave 1). In wave 2, the number of admissions increased (1503, 35.1%, vs 838, 54.8%) along with nosocomial cases (137, 3.2%, vs 96, 6.3%) compared with wave 1.

**Figure 3:**
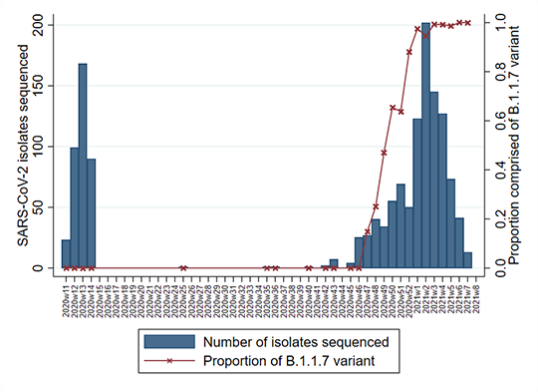
Number of cases with sequenced SARS-CoV-2 isolates by epi-week (bar) and the proportion of which were made up of the Alpha variant B.1.1.7 (red line).

Figure 3 shows the 1470 successfully sequenced SARS-CoV-2 isolates over time, with 382 from wave 1 and 1088 from wave 2. Sequencing was successful for 216/838 (26%) admitted cases from wave 1, 472/1503 (31%) admitted cases in wave 2, and 121/233 (52%) nosocomial cases. The proportion of Alpha variant increased rapidly after the first Alpha isolate was identified on 15 November 2020, accounting for approximately two-thirds within 3 weeks, and almost 100% (600/617 isolates, 97%) in January 2021. In the second wave, the Alpha variant made up 83% (908/1088) of all sequenced isolates, 85% (400/472) of sequenced isolates from admitted cases and 88% (51/59) of sequenced isolates from nosocomial cases. In addition, two cases of the B.1.351 beta variant of concern were also detected in the wave 2 admission cohort.

### Comparison of characteristics of admitted cases between waves 1 and 2

Descriptive statistics of cases admitted during wave 1 (n=838) and wave 2 (n=1503) were compared (table 1). There was a statistically significant difference in median age of 2 years (62 years in wave 1 vs 60 years in wave 2, p=0.019), and admitted cases were more likely to be female in wave 2 (47.3% vs 41.8%, p=0.011). A larger proportion of admitted cases in wave 2 were obese (29.1% vs 24.6%, p=0.02). Comparison of comorbidities showed that those in wave 2 were less likely to have a diagnosis of frailty (11.5% vs 22.8%, p<0.001), history of stroke (4.3% vs 8.6%, p<0.001) or cancer (4.8% vs 7.2%, p=0.022). There was no significant difference in proportion with known comorbidities of diabetes, kidney disease, hypertension, cardiovascular disease or respiratory disease.

**Table 1:** Descriptive statistics of the cohort for wave 1 (n=838) and wave 2 (n=1503) admissions

	Missing	Wave 1 n (%)	Wave 2 n (%)	Wave 1 Median (IQR)	Wave 2 Median (IQR)	P value
**Demographics**						
Age (years)	0			62.0 (49.0–78.0)	60.0 (47.0–74.0)	0.019
Male	0	488 (58.2)	792 (52.7)			0.011
Ethnicity	0					0.013
White		331 (39.5)	598 (39.8)			
Asian		64 (7.6)	121 (8.1)			
Black–African		177 (21.1)	262 (17.4)			
Black–Caribbean		73 (8.7)	98 (6.5)			
Mixed		15 (1.8)	18 (1.2)			
Other		45 (5.4)	107 (7.1)			
Unknown		133 (15.9)	299 (19.9)			
BMI	577			27.0 (23.8–31.7)	27.7 (24.0–32.9)	0.022
>30		206 (24.6)	438 (29.1)			0.02
>40		34 (4.1)	86 (5.7)			0.098
**Physiological parameters**						
Heart rate (beats/min)	360			84.0 (75.0–94.0)	81.0 (72.0–91.0)	<0.001
>100		105 (12.5)	142 (9.4)			0.02
Blood pressure (mm Hg)						
Systolic	369			125.0 (113.0–139.0)	127.0 (115.0–141.0)	0.013
Diastolic	369			73.0 (65.0–80.0)	75.0 (68.0–82.0)	<0.001
MAP	369			90.7 (82.2–99.0)	92.3 (84.7–101.3)	<0.001
Respiratory rate (breaths/min)	359			19.0 (18.0,22.0)	19.0 (18.0–22.0)	0.764
>20		200 (23.9)	365 (24.3)			0.86
Hypoxia	658	370 (64.3)	726 (65.5)			0.67
Temperature (°C)	361			36.9 (36.4–37.5)	36.6 (36.2–37.2)	<0.001
NEWS2	405					0.86
0		95 (11.3)	173 (11.5)			
1		108 (12.9)	192 (12.8)			
2		117 (14.0)	188 (12.5)			
>2		371 (44.3)	692 (46.0)			
**Laboratory parameters**						
Neutrophils (×10^9^ /L)	8			4.9 (3.4–7.6)	5.0 (3.3–7.5)	0.724
Lymphocytes (×10^9^ /L)	7			0.9 (0.6–1.3)	0.9 (0.6–1.4)	0.741
NLR	8			5.4 (3.1–9.9)	5.4 (3.2–9.8)	0.951
Creatinine (μmol/L)	43			83.0 (64.0–115.0)	86.0 (68.0–117.0)	0.065
Urea (mmol/L)	855			7.0 (4.6–12.2)	6.0 (4.3–9.9)	0.001
Estimated GFR (mL/min)	114			73.0 (48.0–98.0)	69.0 (48.0–89.0)	0.001
Albumin (g/L)	185			37.0 (32.0–40.0)	38.0 (34.0–41.0)	<0.001
CRP (mg/L)	61			74.5 (26.0–148.0)	51.0 (18.0–103.8)	<0.001
D-dimer (mg/L FEU)	1297			1.1 (0.6–3.0)	0.9 (0.5–2.2)	0.001
Ferritin (μg/L)	905			855.0 (394.0–1533.5)	699.0 (342.0–1359.0)	0.05
**Comorbidities**						
Stroke	0	72 (8.6)	64 (4.3)			<0.001
TIA	0	9 (1.1)	20 (1.3)			0.731
Hypertension	0	288 (34.4)	464 (30.9)			0.091
Diabetes	0	246 (29.4)	384 (25.5)			0.052
AF	0	63 (7.5)	115 (7.7)			0.972
IHD	0	146 (17.4)	244 (16.2)			0.495
Heart failure	0	54 (6.4)	105 (7.0)			0.679
COPD	0	64 (7.6)	109 (7.3)			0.796
Asthma	0	74 (8.8)	138 (9.2)			0.835
Cancer	0	60 (7.2)	72 (4.8)			0.022
Kidney disease	0	112 (13.4)	181 (12.0)			0.389
HIV	0	21 (2.5)	36 (2.4)			0.979
Solid organ transplant	0	24 (2.9)	49 (3.3)			0.686
Frailty	0	191 (22.8)	173 (11.5)			<0.001

P value was from Kruskal-Wallis test for continuous variables and χ^2^ test for categorical variables.

AF, atrial fibrillation; BMI, body mass index; COPD, chronic obstructive pulmonary disease; CRP, C reactive protein; FEU, fibrinogen equivalent units; GFR, glomerular filtration rate; IHD, ischaemic heart disease; MAP, mean arterial pressure; NEWS2, National Early Warning Score 2; NLR, neutrophil and lymphocyte ratio; TIA, transient ischaemic attack.

There were no significant differences between waves in the proportion with severe SARS-CoV-2 disease on admission as judged by hypoxia (64.3% in wave 1 vs 65.5% in wave 2, p=0.67) or tachypnoea (respiratory rate >20 breaths/min) (23.9% vs 24.3%, p=0.86). There were small differences in other physiological parameters on admission, some of which reached statistical significance, but differences were not clinically relevant.

Laboratory markers were compared between waves (table 1). There were small but significant differences, such as lower C reactive protein (CRP) (median 51.0 mg/dL, IQR 18.0–103.8, vs 74.5 mg/dL, IQR 26.0–148.0; p<0.001) and lower ferritin (699.0, IQR 342.0–1359.0, vs 855.0, IQR 394.0–1533.5; p=0.05) in wave 2. There were other small statistically significant differences without clear clinical significance, such as a lower D-dimer in wave 2 (0.9 mg/L fibrinogen equivalent units (FEU), IQR 0.5–2.2, vs 1.1 mg/L FEU, IQR 0.6–3.0; p=0.001) and lower estimated glomerular filtration rate (69.0 mL/min, IQR 48.0–89.0, vs 73.0 mL/min, IQR 48.0–98.0; p=0.001), lower urea (6.0 mmol/L, IQR 4.3–9.3, vs 7.0 mmol/L, IQR 4.6–12.2; p=0.001) and higher albumin (38.0 g/L, IQR 34.0–41.0 g/L, vs 37.0 g/L, IQR 32.0–40.0; p<0.001). There was no significant difference with neutrophils, lymphocytes, neutrophil and lymphocyte ratio, creatinine, and glucose.

### Comparison of characteristics of admitted cases infected with Alpha and non-Alpha variants

Given the reported association between increased disease severity and transmission with the Alpha variant, we compared demographic, physiological and laboratory parameters between admitted cases with infection caused by Alpha variant (n=400) with non-Alpha (n=910) variants (table 2).

**Table 2:** Descriptive statistics of the cohort for non-Alpha variant (n=910) and Alpha variant (n-400) admissions

	Missing	Non-Alpha variant n (%)	Alpha variant n (%)	Non-Alpha variant value (IQR)	Alpha variant value (IQR)	P value
**Demographics**						
Age (years)	0			62.0 (49.0–78.0)	64.0 (52.0–78.0)	0.22
Male		530 (58.2)	208 (52.0)			0.042
Ethnicity	0					0.402
White		358 (39.3)	164 (41.0)			
Asian		71 (7.8)	38 (9.5)			
Black–African		191 (21.0)	67 (16.8)			
Black–Caribbean		78 (8.6)	27 (6.8)			
Mixed		16 (1.8)	6 (1.5)			
Other		50 (5.5)	23 (5.8)			
Unknown		146 (16.0)	75 (18.8)			
BMI	334			27.1 (23.8–31.7)	28.1 (24.0–34.2)	0.036
>30		226 (24.8)	121 (30.2)			0.048
>40		36 (4.0)	26 (6.5)			0.063
**Physiological parameters**						
Heart rate (beats/min)	198			84.0 (74.0–94.0)	80.0 (72.0–90.0)	0.001
>100		118 (13.0)	36 (9.0)			0.05
Blood pressure (mm Hg)						
Systolic	201			125.0 (113.0–139.5)	127.0 (115.0–142.0)	0.138
Diastolic	201			73.0 (65.0–80.0)	75.0 (67.0–83.0)	0.01
MAP	201			90.7 (82.3–99.2)	92.7 (84.0–101.7)	0.022
Respiratory rate (breaths/min)	194			19.0 (18.0–21.0)	19.0 (18.0–22.0)	0.591
>20		209 (23.0)	96 (24.0)			0.737
Hypoxia	0	392 (62.5)	217 (70.0)			0.029
Temperature (°C)	199			36.9 (36.4–37.5)	36.6 (36.2–37.1)	<0.001
NEWS2	218					0.038
0		107 (11.8)	43 (10.8)			
1		125 (13.7)	39 (9.8)			
2		127 (14.0)	53 (13.2)			
>2		391 (43.0)	207 (51.7)			
**Laboratory parameters**						
Neutrophils (×10^9^/L)	2			4.9 (3.4–7.6)	4.8 (3.3–6.9)	0.479
Lymphocytes (×10^9^/L)	1			0.9 (0.6–1.3)	0.8 (0.5–1.2)	0.005
NLR	2			5.4 (3.1–9.9)	5.8 (3.5–10.2)	0.195
Creatinine (μmol/L)	16			83.0 (64.0–115.0)	92.0 (74.0–126.0)	<0.001
Urea (mmol/L)	536			6.8 (4.3–12.0)	6.6 (4.4–10.6)	0.573
Estimated GFR (mL/min)	43			73.0 (48.5–97.0)	63.5 (44.0–81.0)	<0.001
Albumin (g/L)	107			37.0 (33.0–41.0)	38.0 (34.0–41.0)	0.009
CRP (mg/L)	21			70.0 (25.0–142.0)	54.0 (24.0–102.0)	<0.001
D-dimer (mg/L FEU)	727			1.1 (0.6–2.8)	0.9 (0.5–1.9)	0.019
Ferritin (μg/L)	501			815.0 (366.2–1499.0)	712.0 (357.5–1294.0)	0.341
**Comorbidities**						
Stroke	0	74 (8.1)	22 (5.5)			0.117
TIA	0	12 (1.3)	5 (1.2)			0.87
Hypertension	0	315 (34.6)	144 (36.0)			0.674
Diabetes	0	267 (29.3)	106 (26.5)			0.326
AF	0	72 (7.9)	42 (10.5)			0.154
IHD	0	162 (17.8)	78 (19.5)			0.513
Heart failure	0	61 (6.7)	34 (8.5)			0.299
COPD	0	74 (8.1)	32 (8.0)			0.977
Asthma	0	84 (9.2)	39 (9.8)			0.846
Cancer	0	64 (7.0)	21 (5.2)			0.278
Kidney disease	0	122 (13.4)	62 (15.5)			0.359
HIV	0	22 (2.4)	10 (2.5)			0.916
Solid organ transplant	0	25 (2.7)	19 (4.8)			0.092
Frailty	0	204 (22.4)	58 (14.5)			0.001

P value was from Kruskal-Wallis test for continuous variables and χ^2^ test for categorical variables.

AF, atrial fibrillation; BMI, body mass index; COPD, chronic obstructive pulmonary disease; CRP, C reactive protein; FEU, fibrinogen equivalent units; GFR, Glomerular Filtration Rate; IHD, ischaemic heart disease; MAP, mean arterial pressure; NEWS2, National Early Warning Score 2; NLR, neutrophil and lymphocyte ratio; TIA, transient ischaemic attack.

Groups with non-Alpha and Alpha variants were not significantly different in median age (62 years vs 64 years, p=0.22) or ethnicity. The proportion of admissions who were female was larger in the group infected with the Alpha variant compared with those infected by non-Alpha variants (48.0% vs 41.8%, p=0.01).

Cases infected with the Alpha variant were less likely to be frail (14.5% vs 22.4%, p=0.001). A higher proportion of those in the Alpha variant group were obese (30.2% v 24.8%, p=0.048). Other minor differences in comorbidities between groups are shown in table 2 but did not reach statistical significance.

On admission, a higher proportion of those infected with the Alpha variant were hypoxic (70.0% vs 62.5%, p=0.029), the main indicator of severe disease. CRP on admission was lower in the Alpha variant group (54 mg/L, IQR 24.0–102.0) compared with those infected with non-Alpha variants (70 mg/L, IQR 25.0–142.0; p<0.001). Differences in other laboratory parameters did not meet either statistical or clinical significance.

### Multivariate analysis of factors associated with severity of COVID-19 on admission

Multivariate logistic regression was applied to look at associations with severity of disease on admission as measured by hypoxia (table 3), equivalent to WHO ordinal scale of ≥4.^12^ Age, sex, ethnicity, comorbidities and variant status (Alpha vs non-Alpha) were entered into the model. Severity of disease on admission, as measured by hypoxia, was the outcome variable. Age was a significant predictor of severity, with an OR of 1.02 (95% CI 1.01 to 1.03, p<0.001) for hypoxia on admission for every advancing year. Obesity was associated with severity, giving an OR of 1.70 (95% CI 1.28 to 2.26, p<0.001). Infection with the Alpha variant was also associated with increased hypoxia on admission (OR 1.68, 95% CI 1.26 to 2.24; p<0.001). Other variables were not significantly associated with hypoxia on admission, including sex, ethnicity and comorbidities.

**Table 3:** ORs for severity (hypoxia) at admission from multivariate logistic regression model

	OR	P value	95% CI
Age	1.02	<0.001	1.01 to 1.03
Male	0.96	0.75	0.73 to 1.25
Ethnicity			
Non-white	1.15	0.35	0.86 to 1.55
Unknown	1.20	0.36	0.81 to 1.77
Comorbidity			
Body mass index >30	1.70	<0.001	1.28 to 2.26
Cardiovascular	0.79	0.15	0.58 to 1.09
Hypertension	1.11	0.52	0.81 to 1.51
Diabetes	0.75	0.07	0.55 to 1.02
Chronic respiratory disease	1.20	0.32	0.83 to 1.74
Cancer	0.60	0.06	0.35 to 1.02
Kidney disease	0.74	0.17	0.48 to 1.14
HIV	1.74	0.16	0.80 to 3.78
Organ transplant	0.79	0.55	0.37 to 1.71
Frailty	0.96	0.85	0.64 to 1.45
Alpha variant	1.68	<0.001	1.26 to 2.24

### Comparison of non-sequenced and sequenced cases in wave 2

We assessed for differences between the non-sequenced and sequenced inpatient cases to identify any possible bias in those that were sequenced. Demographics, admission physiological and laboratory parameters, and the outcome measure of hypoxia on admission are presented in table 4. There was no significant difference of the proportion with the outcome measure, hypoxia on admission, in both the sequenced and non-sequenced inpatient groups (47% vs 50%, p=0.381). There was no significant difference in the proportion of men in the sequenced group compared with the non-sequenced group (52.2% vs 53.8%, p=0.595) as with obesity (39.5% vs 38.4%, p=0.783) or the proportion of those from non-white ethnic backgrounds (41.4% vs 40.5%, p=0.934). On average, sequenced inpatient cases were significantly older (63 vs 57 years, p<0.001) and had a larger proportion of some comorbidities than non-sequenced cases.

**Table 4:** Patient characteristics of sequenced and non-sequenced inpatients in wave 2

	Non-sequenced	Sequenced	P value
n	1031	472	
Age (SD)	57.3 (21.0)	62.9 (19.9)	<0.001
Male (%)	538 (52.2)	254 (53.8)	0.595
Ethnicity (%)			0.934
White	418 (40.5)	194 (41.1)	
Non-white	417 (40.4)	192 (40.7)	
Unknown	196 (19.0)	86 (18.2)	
Comorbidities			
Body mass index >30 (%)	302 (38.4)	139 (39.5)	0.783
Cardiovascular (%)	218 (21.1)	142 (30.1)	<0.001
Hypertension (%)	300 (29.1)	172 (36.4)	0.005
Diabetes (%)	269 (26.1)	127 (26.9)	0.787
Chronic respiratory disease (%)	143 (13.9)	82 (17.4)	0.091
Cancer (%)	46 (4.5)	26 (5.5)	0.452
Kidney disease (%)	116 (11.3)	74 (15.7)	0.021
HIV (%)	26 (2.5)	11 (2.3)	0.966
Organ transplant (%)	31 (3.0)	18 (3.8)	0.509
Frailty (%)	108 (10.5)	76 (16.1)	0.003
Hypoxia (%)	491 (47.6)	237 (50.2)	0.381

## Discussion

Our data from a large, multisite healthcare institution in one of the worst affected regions internationally provide a large dataset for in-depth comparison; for instance, we report a similar number of cases as reported from a national observational cohort study from Japan.^18^ Our hospitalised cohort shares similar demographics to other city populations in the UK, representative of London with around 40% of individuals from non-white ethnicities.^19^ This compares to national population studies where the average age of cases was much lower and with lower proportion from non-white ethnicities.^8 20^

There were threefold more SARS-CoV-2 RNA positive cases reported by the hospital laboratory in wave 2. Partly, this is attributed to increased testing capacity and changing testing strategy throughout 2020 (online supplemental figure 1). Due to capacity limits, during wave 1, it was not local policy to offer testing to outpatients and those not requiring admission, instead relying on clinical diagnosis. HCWs were not offered occupational health testing until the end of wave 1. We therefore restricted comparison to inpatient and nosocomial cases.

There were almost twice as many admitted cases in wave 2 compared with wave 1 (1503 vs 838). This is consistent with a higher local community incidence as reported by the ONS infection survey with 3.5% of individuals in London infected in January 2021,^21^ compared with 2.2% of individuals in London at the peak of wave 1.^1^ The increase in peak hospital occupancy in wave 2 has also been reported nationally.^22^ A major contributor to this increase in hospital admissions is likely to be the emergence of the Alpha variant, which is reported to be more transmissible.^7^

Our finding is the first study in hospitalised cohorts to show increased severity of disease with the Alpha variant, as defined by hypoxia on admission, which is equivalent to WHO ordinal scale of ≥4^12^ and a key marker of severe disease. The validity of using hypoxia as a marker of severity is shown by the clinical characteristics of SARS-CoV-2, with respiratory illness causing hypoxia in a minority of cases and with a smaller proportion having respiratory failure necessitating ventilation.^23^ Hypoxia on admission was chosen as a marker of severity to prevent confounding of results by changes in management of hospitalised patients across the pandemic. For instance steroid treatment, which was introduced during the study period around November 2020, have been shown to reduce risk of ventilation and death.^24^ Other improvements in management, such as proning, anticoagulation and tocilizumab, could also confound other severity outcomes like death and intensive care unit (ICU) admission. Hypoxia on admission is not at risk of confounding by changes in management of cases, as currently no significant management or treatment options are deployed in the community.

Our finding of increased severity with the Alpha variant is consistent with that reported in community studies, which show increased hospitalisation^20^ and mortality^8^ with a similar hazard to which we find here for hypoxia on admission. Notably however, these community studies failed to control for comorbidities.^8 20^ The association with severity we find persists even after adjustment for age, sex and comorbidities. Moreover, testing in the first wave prior to emergence of the Alpha variant was strict due to limited testing capacity, potentially leading to an ascertainment bias towards more severe cases in the first wave. In comparison, in the second wave, testing was more widespread, potentially leading to increased ascertainment of less severe cases. This makes it even more striking that the association of the Alpha variant, which dominated the second wave, with severe disease is so prominent.

Notably, the only other published study in hospital cohorts showed no difference in severity as measured by the composite outcome of need for ventilation or death.^10^ Broadly, the two cohorts from these hospital cohorts are similar, with an average age of around 60 and a high proportion of non-white ethnicities. In general, this supports the external validity of our findings, but replication in dissimilar cohorts are awaited. The difference between findings in our study and those of Frampton *et al*^10^ may be related to the choice of outcome. Our choice of outcome, hypoxia on admission, represents the natural history of disease prior to medical intervention as no treatments are currently deployed in the community. The mortality outcome investigated by Frampton *et al* is after hospital treatment, which may ameliorate the severity increase that we find with the Alpha variant, thereby explaining the differences in severity seen between our studies. Interestingly, despite male sex being widely reported to be a risk factor for severe disease, our multivariate model confirms findings by these authors that sex is not significantly associated with severity in hospitalised cohorts after adjusted analysis.^10^

The lack of association between severity and male sex may correspond to the increase in the proportion of women in the admitted cohort of wave 2 and those infected with Alpha, accounting for an extra 5% of admissions with SARS-CoV-2 infection. A study in press^25^ suggests the Alpha variant may be more severe in hospitalised women who may have increased mortality and/or requirement for ICU care. Our data, showing an increase in the proportion of women in the admission cohort and lack of expected association of severity with male sex is consistent with the finding that Alpha may show increased virulence in women.

We also included an assessment of bias by comparing characteristics of non-sequenced cases with those successfully sequenced. While sequenced patients were older and more comorbid, there was no significant difference between the proportion with the outcome measure of hypoxia on admission between our sequenced and non-sequenced cases. This suggests no significant bias towards severity in the sequenced group, which was predominantly made up of cases of the Alpha variant.

Admitted cases in wave 2 were also around half as likely to have a diagnosis of frailty, which may be due to fewer admissions from care homes during wave 2, which has been reported both nationally^26^ and internationally.^27^ Additionally, admitted cases were around a third less likely to have cancer in wave 2. Both of these reductions may also be as a result of individuals shielding, and therefore at reduced risk of acquiring SARS-CoV-2 infection. Other differences in comorbidities between waves were small and of unclear clinical significance.

One additional striking observation was the similarity in the number of nosocomial cases in wave 1 (n=96 of 934 (10%) inpatient cases) and wave 2 (n=137 of 1640 (8%) inpatient cases). This incidence of nosocomial infection is a major challenge for UK healthcare institutions, with associated crude mortality at around 30% during the first wave.^28 29^ Interestingly, nosocomial cases in wave 1 increased and started to fall before impact of the main infection control interventions of banning hospital visitors (25 March), introducing universal surgical mask wearing (28 March 2020) and universal regular inpatient screening (after the first wave). In comparison, all these measures were in place prior to the second wave. The similar number of cases in wave 2 may in part be due to increased inpatient screening, which would identify asymptomatic cases, or introduction of the more transmissible Alpha variant, which made up the vast majority of our sequenced nosocomial cases.

Some healthcare institutions report far fewer nosocomial acquisitions; for instance, an academic hospital in Boston, USA, reported only two nosocomial cases in over 9000 admissions.^30^ This could be due to greater availability of side rooms for isolation or their use of N95 masks by HCWs, which may decrease transmission between HCWs and patients. In contrast, current UK public health policy recommends surgical facemasks for patient interactions unless performing aerosol-generating procedures.^31^ For this reason, it will be important to further investigate the factors involved in nosocomial acquisition in both waves.

One limitation of our study is that the population comes from one city, and findings therefore need to be compared with findings in other regions. Our dataset included cases confirmed by SARS-CoV-2 RNA testing in our laboratory and so may miss those diagnosed only clinically. We could not compare outcomes after hospital admission, such as ICU admission or mortality, due to changes in in-hospital management between waves. In addition, we were unable to include some variables associated with severity in other studies due to few cases with these features (eg, pregnancy) or due to poor coding in the dataset (eg, liver disease), which prevents us from commenting on the risk associated with these variables.

The number of cases diagnosed, admissions and nosocomial cases were higher in wave 2 than in wave 1, likely due to the increased incidence caused by the more transmissible Alpha variant. Infection with the Alpha variant was associated with severity as measured by hypoxia on admission, the first such finding in hospitalised cohorts. Our findings support growing evidence that emerging variants may have altered virulence as well as increased transmissibility, with such evidence providing support for public health efforts to contain their spread. More broadly, it also increases understanding of the emergence of novel pathogens as they adapt to human hosts.

10.1136/bmjopen-2021-055474.supp3 Supplementary data

## Supplementary Material

Reviewer comments

Author's manuscript

## Data Availability

Data are available in a public, open access repository. Sequencing data are available on Global Initiative on Sharing Avian Influenza Data (GISAID). Patient-level metadata are not otherwise available due to Research Ethics Committee (REC) approval.
