# Supplementary figures and images for: Descriptive comparison of admission characteristics between pandemic waves and multivariable analysis of the association of the Alpha variant (B.1.1.7 lineage) of SARS-CoV-2 with disease severity in inner London
Source: BMJ Open. 2022 Feb 8;12(2):e055474. doi: 10.1136/bmjopen-2021-055474 (PMC8829842; doi:10.1136/bmjopen-2021-055474)

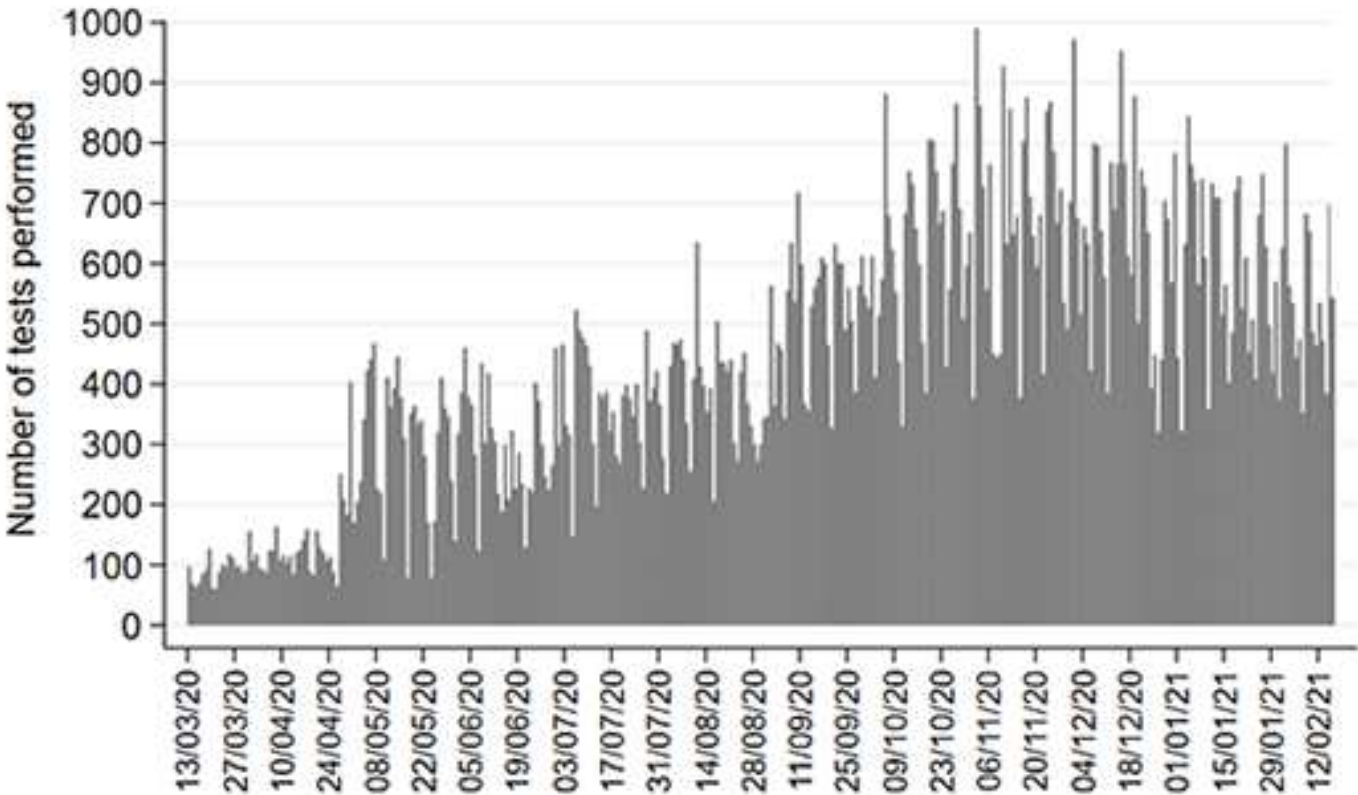

Supplement: Supplementary data [file bmjopen-2021-055474supp001.pdf]
