# Supplementary material for: Descriptive comparison of admission characteristics between pandemic waves and multivariable analysis of the association of the Alpha variant (B.1.1.7 lineage) of SARS-CoV-2 with disease severity in inner London
Source: BMJ Open. 2022 Feb 8;12(2):e055474. doi: 10.1136/bmjopen-2021-055474 (PMC8829842; doi:10.1136/bmjopen-2021-055474)
Supplement: Supplementary data [file bmjopen-2021-055474supp002.pdf]

Supplementary Material

Risk factors for severe disease from COVID-19 - lIterature review

Risk factor	References	Comment on inclusion in multivariable model
Age	[1–5]	Included as a continuous variable.
Sex	[2,5]	Included as binary variable.
Co-morbiidities<br>1. Cancer<br>2. Chronic kidney disease<br>3. Chronic lung disease<br>4. Dementia<br>5. DIabetes<br>6. Cardiac disease<br>7. HIV<br>8. Immunocompromise<br>9. Liver disease<br>10. Obesity<br>11. Pregnancy<br>12. Transplant<br>13. Stroke<br>14. Frailty	1. [2,3,5–7]<br>2. [2,4,6]<br>3. [2,3,5,6]<br>4. [5,6]<br>5. [2,3,6]<br>6. [2,4–6]<br>7. [6,8]<br>8. [2,6]<br>9. [2,5,6]<br>10. [4,6,9–11]<br>11. [6,12]<br>12. [2,6]<br>13. [2,6]<br>14. [13–15]	1. Included<br>2. Included<br>3. Included<br>4. Poorly coded in our dataset so not included.<br>5. Included<br>6. Included<br>7. Included<br>8. Poorly coded in our dataset so not included.<br>9. Poorly coded in our dataset so not included<br>10. Included<br>11. Small number of pregnant individuals in our dataset so not included<br>12. Included<br>13. Included with cardiovascular disease
Ethnicity	[2,5,16,17]	Evidence suggests individuals of non-White ethnicities are at increased risk in multiple territories. Included as categorical variable.
Socioeconomic background	[18,19]	These data suggest risk posed by certain ethnicity may be related to socioeconomic background. For simplicity we included only ethnicity variable.
Variant status	[20–22]	Alpha variant associated with increased mortality in population level studies, but not in single hospitalised study (full discussion in main text)

REFERENCES

1 Richardson S, Hirsch JS, Narasimhan M, *et al.* Presenting Characteristics, Comorbidities, and Outcomes Among 5700 Patients Hospitalized With COVID-19 in the New York City Area. *JAMA* 2020;**323**:2052–9.

2 Williamson EJ, Walker AJ, Bhaskaran K, *et al.* Factors associated with COVID-19-related death using OpenSAFELY. *Nature* 2020;**584**:430–6.

3 Wu Z, McGoogan JM. Characteristics of and Important Lessons From the Coronavirus Disease 2019 (COVID-19) Outbreak in China: Summary of a Report of 72 314 Cases From the Chinese Center for Disease Control and Prevention. *JAMA* 2020;**323**:1239–42.

4 Petrilli CM, Jones SA, Yang J, *et al.* Factors associated with hospital admission and critical illness among 5279 people with coronavirus disease 2019 in New York City: prospective cohort study. *BMJ* 2020;**369**:m1966.

5 Harrison SL, Fazio-Eynullayeva E, Lane DA, *et al.* Comorbidities associated with mortality in 31,461 adults with COVID-19 in the United States: A federated electronic medical record analysis. *PLoS Med* 2020;**17**:e1003321.

6 Know WYNT. People with Certain Medical Conditions. https://www.cdc.gov/coronavirus/2019-ncov/need-

extra-precautions/people-with-medical-conditions.html

7 Dai M, Liu D, Liu M, *et al.* Patients with Cancer Appear More Vulnerable to SARS-CoV-2: A Multicenter Study during the COVID-19 Outbreak. *Cancer Discov* 2020;**10**:783–91.

8 Geretti AM, Stockdale AJ, Kelly SH, *et al.* Outcomes of COVID-19 related hospitalization among people with HIV in the ISARIC WHO Clinical Characterization Protocol (UK): a prospective observational study. *Clin Infect Dis* Published Online First: 23 October 2020. doi:10.1093/cid/ciaa1605

9 Lighter J, Phillips M, Hochman S, *et al.* Obesity in Patients Younger Than 60 Years Is a Risk Factor for COVID-19 Hospital Admission. *Clin Infect Dis* 2020;**71**:896–7.

10 Tartof SY, Qian L, Hong V, *et al.* Obesity and Mortality Among Patients Diagnosed With COVID-19: Results From an Integrated Health Care Organization. *Ann Intern Med* 2020;**173**:773–81.

11 Kompaniyets L, Goodman AB, Belay B, *et al.* Body Mass Index and Risk for COVID-19-Related Hospitalization, Intensive Care Unit Admission, Invasive Mechanical Ventilation, and Death - United States, March-December 2020. *MMWR Morb Mortal Wkly Rep* 2021;**70**:355–61.

12 Allotey J, Stallings E, Bonet M, *et al.* Clinical manifestations, risk factors, and maternal and perinatal outcomes of coronavirus disease 2019 in pregnancy: living systematic review and meta-analysis. *BMJ* 2020;**370**:m3320.

13 Dumitrascu F, Branje KE, Hladkowicz ES, *et al.* Association of frailty with outcomes in individuals with COVID-19: A living review and meta-analysis. *J Am Geriatr Soc* 2021;**69**:2419–29.

14 Kastora S, Kounidas G, Perrott S, *et al.* Clinical frailty scale as a point of care prognostic indicator of mortality in COVID-19: a systematic review and meta-analysis. *EClinicalMedicine* 2021;**36**:100896.

15 Yang Y, Luo K, Jiang Y, *et al.* The Impact of Frailty on COVID-19 Outcomes: A Systematic Review and Meta-analysis of 16 Cohort Studies. *J Nutr Health Aging* 2021;**25**:702–9.

16 Price-Haywood EG, Burton J, Fort D, *et al.* Hospitalization and Mortality among Black Patients and White Patients with Covid-19. *N Engl J Med* 2020;**382**:2534–43.

17 Gold JAW, Rossen LM, Ahmad FB, *et al.* Race, Ethnicity, and Age Trends in Persons Who Died from COVID-19 - United States, May-August 2020. *MMWR Morb Mortal Wkly Rep* 2020;**69**:1517–21.

18 Kabarriti R, Brodin NP, Maron MI, *et al.* Association of Race and Ethnicity With Comorbidities and Survival Among Patients With COVID-19 at an Urban Medical Center in New York. *JAMA Netw Open* 2020;**3**:e2019795.

19 Muñoz-Price LS, Nattinger AB, Rivera F, *et al.* Racial Disparities in Incidence and Outcomes Among Patients With COVID-19. *JAMA Netw Open* 2020;**3**:e2021892.

20 Davies NG, Jarvis CI, CMMID COVID-19 Working Group, *et al.* Increased mortality in community-tested cases of SARS-CoV-2 lineage B.1.1.7. *Nature* 2021;**593**:270–4.

21 Frampton D, Rampling T, Cross A, *et al.* Genomic characteristics and clinical effect of the emergent SARS-CoV-2 B.1.1.7 lineage in London, UK: a whole-genome sequencing and hospital-based cohort study. *Lancet Infect Dis* Published Online First: 12 April 2021. doi:10.1016/S1473-3099(21)00170-5

22 Challen R, Brooks-Pollock E, Read JM, *et al.* Risk of mortality in patients infected with SARS-CoV-2 variant of concern 202012/1: matched cohort study. *BMJ* 2021;**372**:n579.
