# Supplementary material for: Descriptive comparison of admission characteristics between pandemic waves and multivariable analysis of the association of the Alpha variant (B.1.1.7 lineage) of SARS-CoV-2 with disease severity in inner London
Source: BMJ Open. 2022 Feb 8;12(2):e055474. doi: 10.1136/bmjopen-2021-055474 (PMC8829842; doi:10.1136/bmjopen-2021-055474)
Supplement: Supplementary data [file bmjopen-2021-055474supp003.pdf]

Appendix - List of COG-UK HOCI Investigators

Barts site

Name of individual	Employing Institution	Role on HOCI study
**Teresa Cutino-Moguel**	**Barts Heath NHS Trust**	**PI Barts Health**
Tabassum Khan	Barts Heath NHS Trust	Research assistant
Beatrix Kele	Barts Heath NHS Trust	Sequencing scientist
Raghavendran Kulasegaran-Shylini	Barts Heath NHS Trust	Sequencing scientist
Claire E. Broad	Barts Heath NHS Trust	Sequencing scientist
Dola Owoyemi	Barts Heath NHS Trust	Sequencing scientist
David Harrington	Barts Heath NHS Trust	Infection Control Doctor
Clare Coffey	Barts Heath NHS Trust	Infection Control nurse
Martina Cummins	Barts Heath NHS Trust	Infection Control nurse
Anna Riddell	Barts Heath NHS Trust	Virology Consultant
Tyrra D'Souza	Barts Heath NHS Trust	Research Assistant

Glasgow site

Name of individual	Employing Institution	Role on HOCI study
Guy Mollett	MRC-University of Glasgow Centre for Virus Research	Clinical Research Fellow
**Emma Thomson**	**MRC-University of Glasgow Centre for Virus Research and NHS Greater Glasgow and Clyde**	**Principal Investigator**
Christine Peters	NHS Greater Glasgow and Clyde	Microbiology Consultant
Aleks Marek	NHS Greater Glasgow and Clyde	Infection Control Lead/Microbiology Consultant
Rory Gunson	NHS Greater Glasgow and Clyde	Virology laboratory lead
Emily Goldstein	NHS Greater Glasgow and Clyde	Sample extraction
Emilie Shepherd	NHS Greater Glasgow and Clyde	Sample extraction
James Shepherd	MRC-University of Glasgow Centre for Virus Research	Clinical Research Fellow
David Robertson	MRC-University of Glasgow Centre for Virus Research	Lead bioinformatician
Katherine Smollett	MRC-University of Glasgow Centre for Virus Research	Sequencing
Ana da Silva Filipe	MRC-University of Glasgow Centre for Virus Research	Sequencing
Alice Broos	MRC-University of Glasgow Centre for Virus Research	Sequencing
Stephen Carmichael	MRC-University of Glasgow Centre for Virus Research	Sequencing
Nicholas Suarez	MRC-University of Glasgow Centre for Virus Research	Sequencing

Chris Davis	MRC-University of Glasgow Centre for Virus Research	Sample extraction
Sreenu Vattipally	MRC-University of Glasgow Centre for Virus Research	Bioinformatician
Joseph Hughes	MRC-University of Glasgow Centre for Virus Research	Bioinformatician
Ioulia Tsatsani	MRC-University of Glasgow Centre for Virus Research	Bioinformatician
Jacqueline McTaggart	NHS Greater Glasgow and Clyde	Research Nurse
Stephanie McEnhill	NHS Greater Glasgow and Clyde	Research Nurse

Guy's and St Thomas' site

Name of individual	Employing Institution	Role on HOCI study
Adela Medina	Viapath	Sequence
Themoula Charalampous	KCL	Sequence
Bindi Patel	GSTT NHS Trust	Sequence
Flavia Flaviani	GSTT NHS Trust	Bioinformatics
Jörg Saßmannshausen	GSTT NHS Trust	Bioinformatics/IT
May Rabuya	GSTT NHS Trust	Research Nurse-data collection
Sulekha Gurung	GSTT NHS Trust	Research Nurse-data collection
Anu Augustine	GSTT NHS Trust	Research Nurse-data collection
Rahul Batra	GSTT NHS Trust	Sequencing/IT/manager
Luke Snell	GSTT NHS Trust	Sequence, bioinf, data collection, IPC
**Gaia Nebbia**	**GSTT NHS Trust**	**Principal Investigator**

Imperial site

Name of individual	Employing Institution	Role on HOCI study
**Alison Holmes**	**Imperial Healthcare NHS Trust**	**Principal Investigator**
Sid Mookerjee	Imperial Healthcare NHS Trust	Data lead
James Price	Imperial Healthcare NHS Trust	Site IPC Lead
Paul Randell	Imperial Healthcare NHS Trust	Laboratory Lead
Krystal Johnson	Imperial Healthcare NHS Trust	Research Nurse
Thilipan Thaventhiran	Imperial Healthcare NHS Trust	Research Nurse
Damien Mine	Imperial Healthcare NHS Trust	Clinician
Sophie Hunter	Imperial Healthcare NHS Trust	Research Nurse
Isa Ahmad	Imperial Healthcare NHS Trust	Data Analyst
Anitha Ramanathan	Imperial Healthcare NHS Trust	Research Nurse

Liverpool site

Name of individual	Employing Institution	Role on HOCI study
**Anu Chawla**	**Liverpool NHS Foundation Trust**	**Principal Investigator**
Alistair Derby	University of Liverpool	Sequencing lab lead
Sam Haldenby	University of Liverpool	Bioinformatics lead
Becky Taylor	Liverpool NHS Foundation Trust	Research data coordinator
Keith Morris	Liverpool NHS Foundation Trust	Research nurse
Charles Numbere	Liverpool NHS Foundation Trust	Healthcare assistant
Mark Hopkins	Liverpool NHS Foundation Trust	Consultant clinical scientist
Jenifer Mason	Liverpool NHS Foundation Trust	Consultant microbiologist
Alexandra Bailey	Liverpool NHS Foundation Trust	Research administrator
Debbie Lankstead	Liverpool NHS Foundation Trust	Assistant Director of Infection Control
Damian Burns	Liverpool NHS Foundation Trust	Infection Control Nurse

Manchester site

Name of individual	Employing Institution	Role on HOCI study
**Nicholas Machin**	**PHE and MFT**	**Principal Investigator**
Shazaad Ahmad	MFT	Consultant Virologist and IPC Doctor: review of sequencing reports
Julie Cawthorne	MFT	Clinical Director of Infection Prevention and Control: review of sequencing reports and assistance with CRF completion
Ryan George	MFT	IPC surveillance officer: co-ordination of metadata and sequencing reports
James Montgomery	MFT	IPC Nurse: review of sequencing reports and implementation of IPC actions
Deborah McKew	MFT	IPC Nurse: review of sequencing reports and implementation of IPC actions

Newcastle site

Name of individual	Employing Institution	Role on HOCI study
**Yusri Taha**	**Newcastle NHS Trust**	**Site PI**
Angela Cobb	Newcastle NHS Trust	IPC matron
Michelle Ramsay	Newcastle NHS Trust	Infection Control
Maria Leader	Newcastle NHS Trust	Infection Control
Shirelle Burton-Fanning	Newcastle NHS Trust	Virologist
Julie Samuel	Newcastle NHS Trust	Microbiologist and IPC doctor

Sarah Francis	Newcastle NHS Trust	Trial coordinator
Lydia Taylor	Newcastle NHS Trust	Trial's Research Nurse
Darren Smith	Northumbria University	Lead, sequencing
Matthew Bashton	Northumbria University	Bioinformatics lead
Matthew Crown	Northumbria University	Bioinformatics scientist

Nottingham site

Name of individual	Employing Institution	Role on HOCI study
**Nikunj Mahida**	**Nottingham NHS Trust**	**Principal Investigator**
Matthew Loose	University of Nottingham	Sequencing/Bioinfomatics
Patrick McClure	University of Nottingham	Sequencing/Bioinfomatics
Mitch Clarke	Nottingham NHS Trust	IPC - IPC Lead - review of cases, sequencing data
Elaine Baxter	Nottingham NHS Trust	IPC - Senior IPC team member, review of cases, sequencing data
Carl Yates	Nottingham NHS Trust	IPC - Senior IPC team member, review of cases, sequencing data
Irfan Aslam	Nottingham NHS Trust	Data Entry
Vicki Fleming	Nottingham NHS Trust	Sample collection and processing
Michelle Lister	Nottingham NHS Trust	Sample collection and processing
Johnny Debebe	University of Nottingham	Bioinformatics
Nadine Holmes	University of Nottingham	Sequencing
Christopher Moore	University of Nottingham	Sequencing
Matt Carlile	University of Nottingham	Sequencing

Royal Free site

Name of individual	Employing Institution	Role on study
**Tabitha Mahungu**	**Royal Free London NHS Trust**	**Principal Investigator**
Sophie Weller	Royal Free London NHS Trust	Sub-Investigator
Tanzina Haque	Royal Free London NHS Trust	Sub-Investigator
Jennifer Hart	Royal Free London NHS Trust	Sub-Investigator
Dianne Irish-Tavares	Royal Free London NHS Trust	Sub-Investigator
Eric Witele	Royal Free London NHS Trust	Clinical Research Nurse
Mia De Mesa	Royal Free London NHS Trust	Clinical Research Nurse
Vicky Pang	Royal Free London NHS Trust	Head of Infection Prevention & Control Nursing – provided IPC data for CRFs
Jelena Heaphy	Royal Free London NHS Trust	Clinical Lead Nurse Infection Prevention and Control - provided IPC data for CRFs

Wendy Chatterton	Health Services Laboratory	Virology Service Manager, Organised samples & Logistics
Monika Pusok	Health Services Laboratory	Medical laboratory assistant , Organised samples & Logistics

Sandwell site

Name of individual	Employing Institution	Role on HOCI study
**Dr Tranprit Saluja**	**Sandwell & West Birmingham Hospitals NHS Trust**	**Principal Investigator - Consultant Microbiologist and IPC doctor**
Zahira Maqsood	Sandwell NHS Trust	Clinical Research Practitioner
Angie Williams	Sandwell NHS Trust	Research Data Coordinators.
Debbie Devonport	Sandwell NHS Trust	Research Data Coordinators.
Lucy Palinkas	Sandwell NHS Trust	Infection control Data Analyst
Diane Thomlinson	Sandwell NHS Trust	Infection control Nurse
Julie Booth	Sandwell NHS Trust	Lead Nurse IPC
Ashok Dadrah	Sandwell NHS Trust	Laboratory Services Manager
Amanda Symonds	Sandwell NHS Trust	Senior Biomedical Scientist (Microbiology
Cassandra Craig	Sandwell NHS Trust	Laboratory Associate Practitioner
Dr Abhinav Kumar	Sandwell NHS Trust	Consultant microbiologist

Sheffield site

Name of individual	Employing Institution	Role on HOCI study
**Thushan de Silva**	**University of Sheffield**	**Principal Investigator**
Matthew D Parker	University of Sheffield	Bioinformatics processing/management
Peijun Zhang	University of Sheffield	WGS
Max Whiteley	University of Sheffield	WGS
Benjamin B Lindsey	University of Sheffield	WGS
Paige Wolverson	University of Sheffield	WGS
Benjamin H Foulkes	University of Sheffield	WGS
Luke Green	University of Sheffield	WGS
Marta Gallis Ramalho	University of Sheffield	WGS
Stavroula F Louka	University of Sheffield	WGS
Adrienn Angyal	University of Sheffield	WGS
Nikki Smith	University of Sheffield	Management/admin
David G Partridge	Sheffield NHS Trust	Investigator
Cariad Evans	Sheffield NHS Trust	Investigator
Mohammad Raza	Sheffield NHS Trust	Investigator
Hayley Colton	Sheffield NHS Trust	Investigator

Rebecca Gregory	Sheffield NHS Trust	Clinical trial assistant
Phillip Ravencroft	Sheffield NHS Trust	Clinical trial assistant
Katie Johnson	Sheffield NHS Trust	Sample collection and processing
Sharon Hsu	University of Sheffield	Bioinformatics support
Alexander J Keeley	Sheffield NHS Trust	
Alison Cope	Sheffield NHS Trust	
Amy State	Sheffield NHS Trust	Sample collection and processing
Nasar Ali	Sheffield NHS Trust	
Rasha Raghei	Sheffield NHS Trust	
Joe Heffer	Sheffield NHS Trust	
Stella Christou	University of Sheffield	WGS
Samantha E Hansford	University of Sheffield	Management/admin
Hailey R Hornsby	University of Sheffield	WGS
Phil Wade	Sheffield NHS Trust	Data collection
Kay Cawthron	Sheffield NHS Trust	Data collection
Maqsood Khan	Sheffield NHS Trust	Data collection
Amber Ford	Sheffield NHS Trust	Data input
Imogen Wilson	Sheffield NHS Trust	Data input
Kate Harrington	Sheffield NHS Trust	Sample collection
Nic Tinker	Sheffield NHS Trust	Sample collection
Sally Nyinza	Sheffield NHS Trust	Investigator

Southampton site

Name of individual	Employing Institution	Role on study
**Kordo Saeed**	**Southampton NHS Trust**	**Principal Investigator**
Jacqui Prieto	Southampton NHS Trust	Samples/logistics
Adhyana Mahanama	Southampton NHS Trust	Samples/logistics
Buddhini Samaraweera	Southampton NHS Trust	Samples/logistics
Siona Silviera	Southampton NHS Trust	Samples/logistics
Emanuela Pelosi	Southampton NHS Trust	Samples/logistics
Eleri Wilson-Davies	Southampton NHS Trust	Samples/logistics
Sarah Jeremiah	Southampton NHS Trust	Data collection
Helen Wheeler	Southampton NHS Trust	Data collection
Matthew Harvey	Southampton NHS Trust	Data collection
Thea Sass	Southampton NHS Trust	Data collection
Helen Umpleby	Southampton NHS Trust	Data collection
Stephen Aplin	Southampton NHS Trust	Data collection
Samuel Robson	Portsmouth University	Sequencing lead
Sharon Glaysher	Portsmouth Hospital NHS Trust	Sequencing
Scott Elliott	Portsmouth Hospital NHS Trust	Sequencing
Kate Cook	Portsmouth University	Sequencing
Christopher Fearn	Portsmouth University	Sequencing
Salman Goudarzi	Portsmouth University	Sequencing
Katie Loveson	Portsmouth University	Sequencing

St George's site

Name of individual	Employing Institution	Role on HOCI study
Kenneth Laing	St Georges, UoL	Sequencing
Irene Monahan	St Georges, UoL	Sequencing
Adam Witney	St Georges, UoL	Bioinformatician
Joshua Taylor	St Georges NHS Trust	Virology, data collection, CRF completion and upload to MACRO
NgeeKeong Tan	St Georges NHS Trust	Virology, data collection, CRF completion and upload to MACRO
**Cassie Pope**	**St Georges NHS Trust and St Georges, UoL**	**PI, data collection, CRF completion and upload to Macro**
Claudia Cardosa Pereira	St Georges NHS Trust	IPC nurse
Vaz Malik	St Georges, UoL	Upload to macro

UCLH site

Name of individual	Employing Institution	Role on HOCI study
**Gee Yen Shin**	**UCLH NHS Trust**	**Principal Investigator, virologist**
Eleni Nastouli	UCLH NHS Trust	Virologist
Catherine Houlihan	UCLH NHS Trust	Virologist
Judith Heaney	UCLH NHS Trust	Clinical scientist
Matt Byott	UCLH NHS Trust	Bioinformatician
Dan Frampton	UCL / UCLH	Bioinformatician
Gema Martinez-Garcia	UCLH NHS Trust	Senior infection control nurse
Leila Hail	UCLH NHS Trust	Senior infection control nurse
Ndifreke Atang	UCLH NHS Trust	Clinical trials practitioner
Helen Francis	UCLH NHS Trust	Research nurse
Milica Rajkov	UCLH NHS Trust	Clinical trials co-ordinator

UCL Genomics

Name of individual	Employing Institution	Role on HOCI study
**Judith Breuer**	**UCL**	**Chief Investigator**
Rachel Williams	UCL	Sequencing
Sunando Roy	UCL	Sequencing
Charlotte Williams	UCL	Sequencing
Nadua Bayzid	UCL	Sequencing
Marius Cotic	UCL	Sequencing

UCL Comprehensive Clinical Trials Unit

Name of individual	Employing Institution	Role on HOCI study
James Blackstone	UCL	Project Manager
Leanne Hockey	UCL	Trial Manager

Alyson MacNeil	UCL	Trial Manager
Rachel McComish	UCL	Data Analyst
Monica Panca	UCL	Health Economist
Georgia Marley	UCL	Data Manager

UCL Institute for Global Health

Name of individual	Employing Institution	Role on HOCI study
Andrew Copas	UCL	Senior Statistician
Oliver Stirrup	UCL	Statistician
Fiona Mapp	UCL	Qualitative Researcher

UCL Research IT Services

Name of individual	Employing Institution	Role on HOCI study
Alif Tamuri	UCL	IT Developer
Stefan Piatek	UCL	IT Developer

University of Strathclyde

Name of individual	Employing Institution	Role on HOCI study
Paul Flowers	UoS	Senior Qualitative Researcher

Francis Crick Institute

Name of individual	Employing Institution	Role on HOCI study
Marg Crawford	Francis Crick Institute	Sample processing/sequencing
Laura Cubitt	Francis Crick Institute	Sample processing/sequencing
Deborah J Jackson	Francis Crick Institute	Sample processing/sequencing
Jimena Perez-Lloret	Francis Crick Institute	Sample processing/sequencing
Sophie Ward	Francis Crick Institute	Sample processing/sequencing
Makis Fidanis	Francis Crick Institute	Sample processing/sequencing
Aaron Sait	Francis Crick Institute	Sample processing/sequencing
Robert Goldstone	Francis Crick Institute	Data Processing
Harshil Patel	Francis Crick Institute	Data Processing
Chelsea Sawyer	Francis Crick Institute	Data Processing
Aengus Stewart	Francis Crick Institute	Data Processing
Steve Gamblin	Francis Crick Institute	Methodology/Supervision
Charles Swanton	Francis Crick Institute	Methodology/Supervision
Jerome Nicod	Francis Crick Institute	Methodology/Supervision
