# Supplementary material for: Descriptive comparison of admission characteristics between pandemic waves and multivariable analysis of the association of the Alpha variant (B.1.1.7 lineage) of SARS-CoV-2 with disease severity in inner London
Source: BMJ Open. 2022 Feb 8;12(2):e055474. doi: 10.1136/bmjopen-2021-055474 (PMC8829842; doi:10.1136/bmjopen-2021-055474)
Supplement: Author's manuscript [file bmjopen-2021-055474.draft_revisions.pdf]

BMJ Open

BMJ Open is committed to open peer review. As part of this commitment we make the peer review history of every article we publish publicly available.

When an article is published we post the peer reviewers' comments and the authors' responses online. We also post the versions of the paper that were used during peer review. These are the versions that the peer review comments apply to.

The versions of the paper that follow are the versions that were submitted during the peer review process. They are not the versions of record or the final published versions. They should not be cited or distributed as the published version of this manuscript.

BMJ Open is an open access journal and the full, final, typeset and author-corrected version of record of the manuscript is available on our site with no access controls, subscription charges or pay-per-view fees (http://bmjopen.bmj.com).

If you have any questions on BMJ Open's open peer review process please email info.bmjopen@bmj.com

First and second SARS-CoV-2 waves in inner London: A comparison of admission characteristics and the association of the Alpha variant with disease severity.

Journal:	*BMJ Open*
Manuscript ID	bmjopen-2021-055474
Article Type:	Original research
Date Submitted by the Author:	13-Jul-2021
Complete List of Authors:	Snell, Luke; King's College London; Guy's and St Thomas' NHS Foundation Trust<br>Wang , Wenjuan; King's College London<br>Medina, Adela; Viapath; King's College London<br>Charalampous, Themoula; King's College London<br>Nebbia, Gaia; King's College London; Guy's and St Thomas' NHS Foundation Trust<br>Batra, Rahul; King's College London<br>de Jongh, Leonardo; Guy's and St Thomas' NHS Foundation Trust<br>Higgins, Finola; Guy's and St Thomas' NHS Foundation Trust<br>Wang, Yanzhong; King's College London<br>Edgeworth, Jonathan; King's College London; Guy's and St Thomas' NHS Foundation Trust<br>Curcin, Vasa; King's College London
Keywords:	VIROLOGY, EPIDEMIOLOGY, Epidemiology < INFECTIOUS DISEASES, Public health < INFECTIOUS DISEASES

I, the Submitting Author has the right to grant and does grant on behalf of all authors of the Work (as defined in the below author licence), an exclusive licence and/or a non-exclusive licence for contributions from authors who are: i) UK Crown employees; ii) where BMJ has agreed a CC-BY licence shall apply, and/or iii) in accordance with the terms applicable for US Federal Government officers or employees acting as part of their official duties; on a worldwide, perpetual, irrevocable, royalty-free basis to BMJ Publishing Group Ltd ("BMJ") its licensees and where the relevant Journal is co-owned by BMJ to the co-owners of the Journal, to publish the Work in this journal and any other BMJ products and to exploit all rights, as set out in our licence.

The Submitting Author accepts and understands that any supply made under these terms is made by BMJ to the Submitting Author unless you are acting as an employee on behalf of your employer or a postgraduate student of an affiliated institution which is paying any applicable article publishing charge ("APC") for Open Access articles. Where the Submitting Author wishes to make the Work available on an Open Access basis (and intends to pay the relevant APC), the terms of reuse of such Open Access shall be governed by a Creative Commons licence – details of these licences and which Creative Commons licence will apply to this Work are set out in our licence referred to above.

Other than as permitted in any relevant BMJ Author's Self Archiving Policies, I confirm this Work has not been accepted for publication elsewhere, is not being considered for publication elsewhere and does not duplicate material already published. I confirm all authors consent to publication of this Work and authorise the granting of this licence.

For peer review only

First and second SARS-CoV-2 waves in inner London: A comparison of admission characteristics and the association of the Alpha variant with disease severity.

Luke B.Snell MBBS[*], Wenjuan Wang PhD[*], Adela Alcolea-Medina MPhil, Themoula Charalampous PhD, Gaia Nebbia PhD, Rahul Batra MD, Leonardo de Jongh, Finola Higgins, Yanzhong Wang PhD, Jonathan D. Edgeworth PhD[♯], Vasa Curcin PhD[♯]

1 King's College London, London, UK (L.B. Snell, W. Wang, A. Alcolea-Medina, T. Charalampous, G. Nebbia, R. Batra, Y. Wang, J.D. Edgeworth, V Curin)

2 Guy's and St Thomas' NHS Foundation Trust, London, UK (L.B. Snell, G. Nebbia, R. Batra, L. de Jongh, F. Higgins, J.D. Edgeworth)

3 Viapath, St Thomas' Hospital, London, UK (A. Alcolea-Medina)

* These first authors contributed equally

♯ These authors were co–principal investigators

Corresponding author: Dr. Luke B Snell, St. Thomas' Hospital, Westminster Bridge Road, UK SE1 7EH, luke.snell@nhs.net, 02071887188

Running title: Comparison of SARS-CoV-2 waves and alpha variant severity

Article word count: 3714

For peer review only

Abstract

Background. Current literature is unclear on whether the Alpha variant is associated with increased severity. We linked clinical data with viral genome sequence data to compare admitted cases between SARS-CoV-2 waves in London, and to investigate the association between Alpha variant and the severity of disease.

Methods. Clinical, demographic, laboratory and viral sequence data from electronic health record (EHR) systems was collected for all cases with a positive SARS-CoV-2 RNA test between March 13th 2020 and February 17th 2021 in a multi-site London healthcare institution. Univariate and multivariate analysis using logistic regression assessed risk factors for severity at admission as defined by hypoxia.

Results There were 5810 SARS-CoV-2 RNA positive cases. 2341 cases which were admitted, with 838 in wave one and 1503 in wave two. Both waves had a temporally aligned rise in nosocomial cases (n=96 in wave one, n=137 in wave two). The Alpha variant was first identified on 15th November 2020 and increased rapidly to comprise 400/472 (85%) of sequenced isolates from admitted cases in wave two. A multivariate analysis identified risk factors for severity on admission, such as age (OR 1.02 [CI 1.01-1.03] for every year older, p<0.001), obesity (OR 1.7 [CI 1.28-2.26], p<0.001) and infection with the Alpha variant (OR 1.68 [CI 1.26-2.24], p<0.001).

Conclusions Our analysis is the first in hospitalised cohorts to show increased severity of disease associated with the Alpha variant. The number of nosocomial cases was similar in both waves despite the introduction of many infection control interventions before wave two.

Abstract word count: 250

For peer review only

Strengths and limitations of this study

- Published evidence on whether the alpha variant of SARS-CoV-2 has a higher case fatality is mixed.
- Our study benefits from a long study window, including patients since the beginning of the SARS-CoV-2 pandemic
- We combine patient-level data with routinely performed genomic data, capturing a large proportion of inpatients with confirmed SARS-CoV-2 infection.
- Our analysis adjusts for co-morbidities, a feature missing from many of the population-level studies currently published.

For peer review only

Ethics

Ethical approval for data informatics was granted by The London Bromley Research Ethics Committee (reference (20/HRA/1871)) to the King's Health Partners Data Analytics and Modelling COVID-19 Group to collect clinically relevant data points from patient's electronic health records.

Whole genome sequencing of residual viral isolates was conducted under the COVID-19 Genomics UK (COG-UK) consortium study protocol, which was approved by the Public Health England Research Ethics and Governance Group (reference: R&D NR0195).

Acknowledgements

The authors acknowledge use of the research computing facility at King's College London, Rosalind (https://rosalind.kcl.ac.uk), which is delivered in partnership with the National Institute for Health Research (NIHR) Biomedical Research Centres at South London & Maudsley and Guy's & St. Thomas' NHS Foundation Trusts, and part-funded by capital equipment grants from the Maudsley Charity (award 980) and Guy's & St. Thomas' Charity (TR130505). The views expressed are those of the author(s) and not necessarily those of the NHS, the NIHR, King's College London, or the Department of Health and Social Care.

Funding

FH, LBS, YW, and VC are supported by the National Institute for Health Research (NIHR) Biomedical Research Centre programme of Infection and Immunity (RJ112/N027) based at Guy's and St Thomas' National Health Service NHS) Foundation Trust and King's College London.

This work was also supported by The Health Foundation and the Guy's and St Thomas' Charity.

COG-UK is supported by funding from the Medical Research Council (MRC) part of UK Research & Innovation (UKRI), the National Institute of Health Research (NIHR) and Genome Research Limited, operating as the Wellcome Sanger Institute.

For peer review only

Data sharing

Genomic data is publicly available on GISAID, please contact the authors for accession numbers.

Our ethical approval precludes public sharing of patient-level data.

Patient and public involvement

As this study was conducted to meet an urgent public health need patient and public involvement was not sought

Competing interests

None to declare.

For peer review only

Background

SARS-CoV-2 infection has led to the death of over 1 million individuals worldwide since its emergence in China during December 2019, with over 120,000 deaths reported in the UK. In London, the estimated incidence in the first wave peaked around March 23rd at 2.2% [1] and then rapidly declined following non-pharmacological interventions. Hospital admissions peaked about 1 week later [2] reflecting the median period of symptoms before hospital presentation. A “second wave” of infections started in London around the beginning of October [3].

Genome sequencing identified the Alpha variant (the B.1.1.7 lineage) around the South East of England, which spread rapidly as part of the emerging second wave. [4] This occurred prior to widespread vaccination, with only 25% of the adult population receiving the first dose by mid-February 2021 [5]. The Alpha variant has been associated with increased transmissibility in community studies [6] [7], and community studies associate the variant with increased mortality [8] [9]. However, the single published study in hospitalised patients suggested no increase in need for ventilation or mortality [10].

Changes in transmissibility and severity have the potential to affect the burden on healthcare systems, and modify the characteristics of cases presenting to hospitals including the demographics, co-morbidities and severity of disease associated with SARS-CoV-2 infection.

Objectives

We linked clinical datasets with local SARS-CoV-2 variant analysis to compare admission characteristics of hospitalised cases during the two waves of infection and to look at the association of the alpha variant with the severity of disease at presentation to hospital.

For peer review only

Methods

Setting

Guy's and St Thomas' NHS Foundation Trust (GSTT) is a multi-site healthcare institution providing general and emergency services predominantly to the South London boroughs of Lambeth and Southwark. An acute-admitting site (St Thomas' Hospital) has an adult emergency department, with a large critical care service including one of the UK's eight nationally commissioned ECMO centres for severe respiratory failure. A second site (Guy's Hospital) provides more inpatient services such as elective surgery, cancer care and other specialist services. A paediatric hospital (Evelina London) acts as a general and specialised referral centre. Several satellite sites for specialist services like dialysis, rehabilitation and long term care are also part of the institution. GSTT receives patients from regional hospitals predominantly to critical care through ‘mutual aid' schemes.

SARS-CoV-2 laboratory testing

Our laboratory began testing on 13th March 2020 with initial capacity for around 150 PCR tests per day, before increasing to around 500 tests per day in late April during wave one, and up to 1000 tests per day during wave two (Supplementary Figure 1).

Testing commenced during the first wave on 13th March 2020 limited to cases requiring admission or inpatients who had symptoms of fever or cough, as per national recommendation; guidance suggested cases who did not require admission should not be tested. For wave two, all cases admitted to hospital were screened and underwent universal interval screening at varying time points. Staff testing for symptomatic healthcare workers was also introduced towards the end of wave one. Comparative analysis was therefore restricted to SARS-CoV-2 RNA positive cases requiring admission. Cases without laboratory confirmation of SARS-CoV-2 infection were not included.

Assays used for the detection of SARS-CoV-2 RNA include PCR testing using Aus Diagnostics or by the Hologic Aptima SARS-CoV-2 Assay. Nucleic acid was first extracted using the QIAGEN

For peer review only

QIAsymphony SP system and a QIAsymphony DSP Virus/Pathogen Mini Kit (catalogue No: 937036) with the off-board lysis protocol.

Definitions and participants

Cases were identified by the first positive SARS-CoV-2 RNA test. Cases were placed in mutually exclusive categories with the following definitions: 1) outpatients 2) testing through occupational health 3) emergency department attenders not subsequently admitted within 14 days 4) patients admitted within 14 days of a positive test 5) nosocomial cases, defined based on ECDC definitions, as those having a first positive test on day 8 or later after admission to hospital where COVID-19 was not suspected on admission [11] and 6) interhospital transfers. For the purpose of comparison only the inpatient group, admitted within 14 days following a positive test, were taken forward for onward comparison. A composite datapoint for ‘hypoxia' was created, equivalent to WHO ordinal scale of ≥4, with cases taken to be hypoxic if on admission they had oxygen saturations of <94%, if they were recorded as requiring supplemental oxygen, or if the fraction of inspired oxygen was recorded as being greater than 0.21.

Determination of SARS-CoV-2 lineage

Whole genome sequencing of residual samples from SARS-CoV-2 cases was performed using GridION (Oxford Nanopore Technology), using version 3 of the ARTIC protocol [12] and bioinformatics pipeline [13]. Samples were selected for sequencing if the corrected CT value was 33 or below, or the Hologic Aptima assay was above 1000 RLU. During the first wave sequencing occurred between March 1st - 31st, whilst sequencing in the second wave restarted in November 2020 - March 2021. Variants were called using updated versions of pangolin 2.0 [14].

Data sources, extraction and integration

Clinical, laboratory and demographic data for all cases with a laboratory reported SARS-CoV-2 PCR RNA test on nose and throat swabs or lower respiratory tract specimens were extracted from hospital

For peer review only

electronic health record (EHR) data sources using records closest to the test date. Data was linked to the Index of Multiple Deprivation (IMD). Age, sex and ethnicity were extracted from EPR. Self-reported ONS ethnic categories were stratified into White (British, Irish, Gypsy and White-Other) or non-White (Black [African, Caribbean, and Black-Other], Asian [Bangladeshi, Chinese, Indian, Pakistan, and Asian-Other] and Mixed/Other). Numbers for which data was missing is listed by each variable. Comorbidities and medication history were extracted from the EPR and e-Noting using natural language processing (NLP). If a comorbidity was not recorded it was assumed not to be present. Cases were characterised as having/not having a past medical history of hypertension, cardiovascular disease (stroke, transient ischaemic attack, atrial fibrillation, congestive heart failure, ischaemic heart disease, peripheral artery disease or atherosclerotic disease), diabetes mellitus, chronic kidney disease, chronic respiratory disease (chronic obstructive pulmonary disease, asthma, bronchiectasis or pulmonary fibrosis) and neoplastic disease (solid tumours, haematological neoplasias or metastatic disease). Obesity was defined as either obesity present in the notes, or recorded Body Mass Index (BMI) >= 30 kg/m^2. Medicines data were extracted using both structured queries and natural language processing tools with medical and drug dictionaries .Additionally, checks on free text data were performed by a cardiovascular clinician to ensure the information was accurate.

Analysis was carried out on the secure Rosalind high-performance computer infrastructure [15] running Jupyter Notebook 6.0.3, R 3.6.3 and Python 3.7.6.

Statistical analysis and outcome measures

General statistics were summarised with mean and standard deviation (SD) for continuous variables if the distribution is normal and median and interquartile range (IQR) if the distribution is non-normal. Count and percentages were used for categorical variables. For the comparisons of variables for wave one versus two variables, Alpha variant versus non-Alpha variants, as well as sequenced patients versus non-sequenced patients in wave two, Kruskal-Walllis test was used for continuous variables and Chi-squared test for categorical variables with significance level of p=0.05. Multivariate analysis was performed using logistic regression to assess the odds ratios of different risk factors

For peer review only

(including age, sex, ethnicity (White, non-White, Unknown), variant status (Alpha or non-Alpha), and cardiovascular disease, hypertension, diabetes, chronic respiratory disease, cancer, kidney disease, HIV, transplant, and frailty) for hypoxia on admission as the binary outcome indicating severity at admission.

For peer review only

Results

General epidemiology and results of viral sequencing.

Figure 1 shows the incidence of SARS-CoV-2 cases, SARS-CoV-2 admissions, and nosocomial cases since March 13th 2020. In total 5810 individuals had a positive SARS-CoV-2 PCR test up until the data extraction date of 17th February 2021. Two "waves" are evident with July 25th taken as an arbitrary separation date between waves, at which point a minimum of 12 wave one cases remained in hospital. Wave one comprised 1528 unique cases (26.3%) from when laboratory testing commenced on March 13th to peak rapidly between the 1st and 8th April 2020 with 57 new cases, before falling to a baseline by May 12th 2020. 1391/1528 (91%) of all cases in wave one occurred during these 60 days. Wave two comprised 4282 unique cases (73.7%), with incidence first increasing gradually from the beginning of October. There was then a period of rapidly escalating incidence from about 10th December, peaking on 28th December 2020 139 cases were diagnosed. 3446/4282 (80%) of wave two cases detected during a comparable 60 day period ending 8th February 2021. In both waves nosocomial cases peaked early, increasing along with admissions but then fell while the number of community admissions continued at peak levels.

Figure 1: Distribution of laboratory confirmed SARS-CoV-2 cases over time. Daily incidence of new cases (beige), newly admitted cases (orange) and nosocomial acquisitions (green) over time.

Individuals with a positive test were placed into six categories (Figure 2), The 5810 SARS-CoV-2 cases were categorised as follows, (n=2341), healthcare workers (n=1549), outpatients (n=874), emergency department (ED) attenders not subsequently admitted (n=532), inter-hospital transfers (n=281) and nosocomial cases (n=233). Some observed differences between wave one and two reflected the increased availability of testing particularly for outpatients (208;13.6% v 666;15.6%), people sent home from ED (111;7.3% v 421; 9.8%) and healthcare workers (171;11.2% v 1378;32.2%). There were also more interhospital transfers of known COVID-19 cases in wave two (177;4.1% v 104;6.8% in wave one). In wave two, the number of admissions increased (1503; 35.1% v 838; 54.8%) along with nosocomial cases (137;3.2% v 96;6.3%) compared with wave one.

For peer review only

Figure 2 (A) Absolute number of cases within the different hospital cohorts during wave one (upper) and wave two (lower). **(B)** Proportion of cases within the different hospital cohorts during wave one (upper) and wave two (lower).

Figure 3 shows the 1470 successfully sequenced SARS-CoV-2 isolates over time, with 382 from wave one and 1088 from wave two. Sequencing was successful for 216/838 (26%) admitted cases from wave one, 472/1503 (31%) admitted cases in wave two, and 121/233 (52%) nosocomial cases. The proportion of Alpha variant increased rapidly after the first Alpha isolate was identified on 15th November 2020, accounting for approximately two thirds within 3 weeks, and almost 100% (600/617 isolates, 97%) in January 2021. In the second wave, the Alpha variant made up 83% (908/1088) of all sequenced isolates, 85% (400/472) of sequenced isolates from admitted cases, and 88% (51/59) of sequenced isolates from nosocomial cases. In addition, two cases of the B.1.351 beta variant of concern were also detected in the wave two admission cohort.

Figure 3 Number of cases with sequenced SARS-CoV-2 isolates by epi-week (bar) and the proportion of which were made up of the alpha variant B.1.1.7 (red line)

Comparison of characteristics of admitted cases between wave one and two

General statistics of cases admitted during wave one (n=838) and wave two (n=1503) were compared (Table 1). There was only a small difference in mean age (62yrs in wave one v 60yrs in wave two, p=0.019), however admitted cases were more likely to be female in wave two (47.3% v 41.8%, p=0.011). . A larger proportion of admitted cases in wave two were obese (29.1% v 24.6%, p=0.02). Comparison of comorbidities showed those in wave two were less likely to have a diagnosis of frailty (11.5% v 22.8%, p<0.001), history of stroke (4.3% v 8.6%, p<0.001) or cancer (4.8% v 7.2%, p=0.022) There was no significant difference in proportion with known comorbidities of diabetes, kidney disease, hypertension, cardiovascular disease or respiratory disease.

For peer review only

Table 1, General statistics of the cohort for wave one and two admissions

	Missing	Wave one admissions n (%) / value [IQR]	Wave two admissions n (%) / value [IQR]	P-Value
n		838	1503	
Demographics				
Age	0	62.0 [49.0,78.0]	60.0 [47.0,74.0]	0.019
Male	0	488 (58.2)	792 (52.7)	0.011
Ethnicity	0			0.013
White		331 (39.5)	598 (39.8)	
Asian		64 (7.6)	121 (8.1)	
Black-African		177 (21.1)	262 (17.4)	
Black-Caribbean		73 (8.7)	98 (6.5)	
Mixed		15 (1.8)	18 (1.2)	
Other		45 (5.4)	107 (7.1)	
Unknown		133 (15.9)	299 (19.9)	
BMI	577	27.0 [23.8,31.7]	27.7 [24.0,32.9]	0.022
BMI>30		206 (24.6)	438 (29.1)	0.02
BMI>40		34 (4.1)	86 (5.7)	0.098
Physiological parameters				
Heart Rate	360	84.0 [75.0,94.0]	81.0 [72.0,91.0]	<0.001
Heart Rate>100		105 (12.5)	142 (9.4)	0.02
Blood pressure				
Systolic	369	125.0 [113.0,139.0]	127.0 [115.0,141.0]	0.013
Diastolic	369	73.0 [65.0,80.0]	75.0 [68.0,82.0]	<0.001
MAP	369	90.7 [82.2,99.0]	92.3 [84.7,101.3]	<0.001
Respiratory Rate	359	19.0 [18.0,22.0]	19.0 [18.0,22.0]	0.764
Respiratory Rate>20		200 (23.9)	365 (24.3)	0.86
Hypoxia	658	370 (64.3)	726 (65.5)	0.67
Temperature	361	36.9 [36.4,37.5]	36.6 [36.2,37.2]	<0.001
NEWS2	405			0.86
0		95 (11.3)	173 (11.5)	
1		108 (12.9)	192 (12.8)	
2		117 (14.0)	188 (12.5)	
2+		371 (44.3)	692 (46.0)	
Laboratory parameters				
Neutrophils	8	4.9 [3.4,7.6]	5.0 [3.3,7.5]	0.724
Lymphocytes	7	0.9 [0.6,1.3]	0.9 [0.6,1.4]	0.741
NLR	8	5.4 [3.1,9.9]	5.4 [3.2,9.8]	0.951
Creatinine	43	83.0 [64.0,115.0]	86.0 [68.0,117.0]	0.065

For peer review only - http://bmjopen.bmj.com/site/about/guidelines.xhtml

Urea	855	7.0 [4.6,12.2]	6.0 [4.3,9.9]	0.001
Estimated GFR	114	73.0 [48.0,98.0]	69.0 [48.0,89.0]	0.001
Albumin	185	37.0 [32.0,40.0]	38.0 [34.0,41.0]	<0.001
CRP	61	74.5 [26.0,148.0]	51.0 [18.0,103.8]	<0.001
DDimer	1297	1.1 [0.6,3.0]	0.9 [0.5,2.2]	0.001
Ferritin	905	855.0 [394.0,1533.5]	699.0 [342.0,1359.0]	0.05
Co-morbidities				
Stroke	0	72 (8.6)	64 (4.3)	<0.001
TIA	0	9 (1.1)	20 (1.3)	0.731
Hypertension	0	288 (34.4)	464 (30.9)	0.091
Diabetes	0	246 (29.4)	384 (25.5)	0.052
AF	0	63 (7.5)	115 (7.7)	0.972
IHD	0	146 (17.4)	244 (16.2)	0.495
Heart Failure	0	54 (6.4)	105 (7.0)	0.679
COPD	0	64 (7.6)	109 (7.3)	0.796
Asthma	0	74 (8.8)	138 (9.2)	0.835
Cancer	0	60 (7.2)	72 (4.8)	0.022
Kidney disease	0	112 (13.4)	181 (12.0)	0.389
HIV	0	21 (2.5)	36 (2.4)	0.979
Solid organ Transplant	0	24 (2.9)	49 (3.3)	0.686
Frailty	0	191 (22.8)	173 (11.5)	<0.001

Note: p-value was from Kruskal-Wallis test for continuous variables and Chi-squared test for categorical variables.

Abbreviations: BMI: Body Mass Index, TIA: Transient Ischaemic Attack, IHD: Ischemic Heart Disease, COPD: Chronic Obstructive Pulmonary Disease, HIV: Human Immunodeficiency Virus, MAP:Mean Arterial Pressure, SPO2: Oxygen Saturation, GFR: Glomerular Filtration Rate, CRP: C-Reactive Protein, NLR: Neutrophils and Lymphocytes Ratio, NEWS2: National Early Warning Score 2.

For peer review only

There were no significant differences between waves in the proportion with severe SARS-CoV-2 disease upon admission as judged by hypoxia (64.3% in wave one vs 65.5% in wave two, p=0.67) or tachypnoea (respiratory rate>20) (23.9% vs 24.3%, p=0.86). There were small differences in other physiological parameters on admission, some of which reached statistical significance but differences were not clinically relevant.

Laboratory markers were compared between waves (Table 1). There were small but significant differences, such as lower CRP (median 51.0 mg/dL (IQR: 18.0-103.8) v 74.5 mg/dL (IQR: 26.0-148.0), p<0.001) and lower ferritin (699.0 [IQR: 342.0-1359.0] v 855.0 [IQR: 394.0-1533.5], p=0.05) in wave two. There were other small statistically significant differences without clear clinical significance, such as a lower D-Dimer in wave two (0.9 mg/L FEU [IQR: 0.5-2.2] v 1.1 mg/L FEU [IQR:0.6-3.0], p=0.001), and lower estimated GFR (69.0 ml/min [IQR: 48.0-89.0] v 73.0 ml/min [IQR: 48.0-98.0], p=0.001), lower urea (6.0 mmol/L [IQR: 4.3-9.3] v 7.0 mmol/L [IQR: 4.6-12.2], p=0.001) and higher albumin (38.0 g/L [IQR: 34.0-41.0 g/L] vs 37.0 g/L [IQR: 32.0-40.0], p<0.001). There was no significant difference with neutrophils, lymphocytes, neutrophils and lymphocytes ratio (NLR), creatinine and glucose.

Comparison of characteristics of admitted cases infected with Alpha and non-Alpha variants

Given the reported association between increased disease severity and transmission with the Alpha variant, we compared demographic, physiological and laboratory parameters between admitted cases with infection caused by Alpha variant (n=400) compared with non-Alpha (n=910) variants (Table 2). We considered all cases in wave one to be non-Alpha variants, as wave one took place prior to emergence of the Alpha variant and before Alpha variant was first identified in our population in November 2021.

For peer review only

Table 2, General statistics of the cohort for non-Alpha variant and Alpha variant admissions

	Missing	Non B.1.1.7 variant n (%) / value [IQR]	B.1.1.7 variant n (%) / value [IQR]	P-Value
n		910	400	
Demographics				
Age	0	62.0 [49.0,78.0]	64.0 [52.0,78.0]	0.22
Male		530 (58.2)	208 (52.0)	0.042
Ethnicity	0			0.402
White		358 (39.3)	164 (41.0)	
Asian		71 (7.8)	38 (9.5)	
Black-African		191 (21.0)	67 (16.8)	
Black-Caribbean		78 (8.6)	27 (6.8)	
Mixed		16 (1.8)	6 (1.5)	
Other		50 (5.5)	23 (5.8)	
Unknown		146 (16.0)	75 (18.8)	
BMI	334	27.1 [23.8,31.7]	28.1 [24.0,34.2]	0.036
BMI>30		226 (24.8)	121 (30.2)	0.048
BMI>40		36 (4.0)	26 (6.5)	0.063
Physiological parameters				
Heart Rate	198	84.0 [74.0,94.0]	80.0 [72.0,90.0]	0.001
Heart Rate>100		118 (13.0)	36 (9.0)	0.05
Blood pressure				
Systolic	201	125.0 [113.0,139.5]	127.0 [115.0,142.0]	0.138
Diastolic	201	73.0 [65.0,80.0]	75.0 [67.0,83.0]	0.01
MAP	201	90.7 [82.3,99.2]	92.7 [84.0,101.7]	0.022
Respiratory Rate	194	19.0 [18.0,21.0]	19.0 [18.0,22.0]	0.591
Respiratory Rate>20		209 (23.0)	96 (24.0)	0.737
Hypoxia	0	392 (62.5)	217 (70.0)	0.029
Temperature	199	36.9 [36.4,37.5]	36.6 [36.2,37.1]	<0.001
NEWS2	0			0.038
0		107 (11.8)	43 (10.8)	
1		125 (13.7)	39 (9.8)	
2		127 (14.0)	53 (13.2)	
2+		391 (43.0)	207 (51.7)	
nan		160 (17.6)	58 (14.5)	
Laboratory parameters				
Neutrophils	2	4.9 [3.4,7.6]	4.8 [3.3,6.9]	0.479
Lymphocytes	1	0.9 [0.6,1.3]	0.8 [0.5,1.2]	0.005
NLR	2	5.4 [3.1,9.9]	5.8 [3.5,10.2]	0.195

For peer review only - http://bmjopen.bmj.com/site/about/guidelines.xhtml

Creatinine	16	83.0 [64.0,115.0]	92.0 [74.0,126.0]	<0.001
Urea	536	6.8 [4.3,12.0]	6.6 [4.4,10.6]	0.573
Estimated GFR	43	73.0 [48.5,97.0]	63.5 [44.0,81.0]	<0.001
Albumin	107	37.0 [33.0,41.0]	38.0 [34.0,41.0]	0.009
CRP	21	70.0 [25.0,142.0]	54.0 [24.0,102.0]	<0.001
DDimer	727	1.1 [0.6,2.8]	0.9 [0.5,1.9]	0.019
Ferritin	501	815.0 [366.2,1499.0]	712.0 [357.5,1294.0]	0.341
Co-morbidities				
Stroke	0	74 (8.1)	22 (5.5)	0.117
TIA	0	12 (1.3)	5 (1.2)	0.87
Hypertension	0	315 (34.6)	144 (36.0)	0.674
Diabetes	0	267 (29.3)	106 (26.5)	0.326
AF	0	72 (7.9)	42 (10.5)	0.154
IHD	0	162 (17.8)	78 (19.5)	0.513
Heart Failure	0	61 (6.7)	34 (8.5)	0.299
COPD	0	74 (8.1)	32 (8.0)	0.977
Asthma	0	84 (9.2)	39 (9.8)	0.846
Cancer	0	64 (7.0)	21 (5.2)	0.278
Kidney disease	0	122 (13.4)	62 (15.5)	0.359
HIV	0	22 (2.4)	10 (2.5)	0.916
Solid organ transplant	0	25 (2.7)	19 (4.8)	0.092
Frailty	0	204 (22.4)	58 (14.5)	0.001

Note: p-value was from Kruskal-Wallis test for continuous variables and Chi-squared test for categorical variables.

Abbreviations: BMI: Body Mass Index, TIA: Transient Ischaemic Attack, IHD: Ischemic Heart Disease, COPD: Chronic Obstructive Pulmonary Disease, HIV: Human Immunodeficiency Virus, MAP:Mean Arterial Pressure, SPO2: Oxygen Saturation, GFR: Glomerular Filtration Rate, CRP: C-Reactive Protein, NLR: Neutrophils and Lymphocytes Ratio, NEWS2: National Early Warning Score 2.

For peer review only

Groups with non-Alpha and Alpha variants were not significantly different in mean age (62yrs vs 64yrs, p=0.22) or ethnicity. The proportion of admissions who were female was larger in the group infected with the Alpha variant compared to those infected by non-Alpha variants (48.0% vs 41.8%, p=0.01).

Cases infected with the Alpha variant were less likely to be frail (14.5% vs 22.4% p=0.001). A higher proportion of those in the Alpha variant group were obese (30.2% v 24.8%, p=0.048). Other minor differences in comorbidities between groups are shown in Table 2, but did not reach statistical significance.

On admission a higher proportion of those infected with the Alpha variant were hypoxic (70.0% vs 62.5%, p=0.029), the main indicator of severe disease. CRP on admission was lower in the Alpha variant group (54 mg/L IQR: 24.0-102.0) compared to those infected with non-Alpha variants (70 mg/L, IQR: 25.0-142.0 p<0.001). Differences in other laboratory parameters did not meet either statistical or clinical significance.

Multivariate analysis of factors associated with severity of COVID-19 on admission

Multivariate logistic regression was applied to look at associations with severity of disease on admission as measured by hypoxia (Table 3), equivalent to WHO ordinal scale of ≥4. Age, sex, ethnicity, co-morbidities and variant status (Alpha vs non-Alpha) were entered into the model. Severity of disease on admission, as measured by hypoxia, was the outcome variable. Age was a significant predictor of severity, with an odds ratio of 1.02 (CI 1.01-1.03, p<0.001) for hypoxia on admission for every advancing year. Obesity was associated with severity, giving an OR 1.70 (CI 1.28-2.26, p<0.001). Infection with the Alpha variant was also associated with increased hypoxia on admission (OR 1.68 CI: 1.26-2.24, p<0.001). Other variables were not significantly associated with hypoxia on admission, including sex, ethnicity and co-morbidities.

For peer review only

Table 3: Odds ratios for severity (hypoxia) at admission from multivariate logistic regression model

	OR	p-value	95% CI
Age	1.02	<0.001	1.01 - 1.03
Male	0.96	0.75	0.73 - 1.25
Ethnicity			
non-White	1.15	0.35	0.86 - 1.55
Unknown	1.20	0.36	0.81 - 1.77
**Comorbidity**			
BMI>30	1.70	<0.001	1.28 - 2.26
Cardiovascular	0.79	0.15	0.58 - 1.09
Hypertension	1.11	0.52	0.81 - 1.51
Diabetes	0.75	0.07	0.55 - 1.02
Chronic respiratory disease	1.20	0.32	0.83 - 1.74
Cancer	0.60	0.06	0.35 - 1.02
Kidney disease	0.74	0.17	0.48 - 1.14
HIV	1.74	0.16	0.80 - 3.78
Organ transplant	0.79	0.55	0.37 -1.71
Frailty	0.96	0.85	0.64 - 1.45
Alpha variant	1.68	<0.001	1.26 - 2.24

For peer review only

Comparison of non-sequenced and sequenced cases in wave two

We assessed for differences between the non-sequenced and sequenced inpatient cases to identify any possible bias in those that were sequenced. Demographics, admission physiological and laboratory parameters, and the outcome measure of hypoxia on admission are presented in Table 4. There were similar proportions of the outcome measure, hypoxia on admission, in both the sequenced and non-sequenced inpatient group (47% vs 50%, p=0.381). There was no difference in the proportion of males in the sequenced group compared to the non-sequenced group (52.2% vs 53.8%, p=0.595), as with obesity (39.5% vs 38.4%, p=0.783) or the proportion of those from non-White ethnic backgrounds (41.4% vs 40.5%, p=0.934). On average, sequenced inpatient cases were slightly older (63 vs 57 years, p<0.001) and had a larger proportion of some comorbidities than non-sequenced cases.

For peer review only

Table 4, Patient characteristics of sequenced and non-sequenced inpatients in wave two.

	Non-sequenced	Sequenced	P-Value
n	1031	472	
Age	57.3 (21.0)	62.9 (19.9)	<0.001
Male	538 (52.2)	254 (53.8)	0.595
Ethnicity			0.934
White	418 (40.5)	194 (41.1)	
non-White	417 (40.4)	192 (40.7)	
Unknown	196 (19.0)	86 (18.2)	
**Comorbidities**			
BMI>30	302 (38.4)	139 (39.5)	0.783
Cardiovascular	218 (21.1)	142 (30.1)	<0.001
Hypertension	300 (29.1)	172 (36.4)	0.005
Diabetes	269 (26.1)	127 (26.9)	0.787
Chronic respiratory disease	143 (13.9)	82 (17.4)	0.091
Cancer	46 (4.5)	26 (5.5)	0.452
Kidney Disease	116 (11.3)	74 (15.7)	0.021
HIV	26 (2.5)	11 (2.3)	0.966
Organ transplant	31 (3.0)	18 (3.8)	0.509
Frailty	108 (10.5)	76 (16.1)	0.003
Hypoxia	491 (47.6)	237 (50.2)	0.381

For peer review only

Discussion

Our data from a large, multi-site healthcare institution in one of the worst affected regions internationally provides a large dataset for in-depth comparison; for instance we report a similar number of cases as reported from a national observational cohort study from Japan [16]. Our hospitalised cohort shares similar demographics to other city populations in the UK, representative of London with around 40% of individuals from non-White ethnicities [17] . This compares to national population studies where the average age of cases was much lower and with lower proportion from non-White ethnciities [8,18].

There were threefold more SARS-CoV-2 RNA positive cases reported by the hospital laboratory in wave two. Partly this is attributed to increased testing capacity and changing testing strategy throughout 2020 (Supplementary Figure 1). Due to capacity limits, during wave one it was not local policy to offer testing to outpatients and those not requiring admission, instead relying on clinical diagnosis. Healthcare workers were not offered occupational health testing until the end of wave one. We therefore restricted comparison to inpatient and nosocomial cases.

There were almost twice as many admitted cases in wave two compared with wave one (1503 v 838). This is consistent with a higher local community incidence as reported by the ONS infection survey with 3.5% of individuals in London infected in January 2021 [19], compared with 2.2% of individuals in London at the peak of wave one [1]. The increase in peak hospital occupancy in wave two has also been reported nationally [20]. A major contributor to this increase in hospital admissions is likely to be the emergence of the Alpha variant, which is reported to be more transmissible [[7]] .

Our finding is the first study in hospitalised cohorts to show increased severity of disease with the Alpha variant, as defined by hypoxia on admission which is equivalent to WHO ordinal scale of ≥4 and a key marker of severe disease. This is consistent with increased virulence of the Alpha variant as reported in community studies, which show increased hospitalisation [18] and mortality [8] with

For peer review only

the Alpha variant. Notably however, these community studies failed to control for co-morbidities. The association with severity we find persists even after adjustment for age, sex, and co-morbidities. Moreover, testing in the first wave prior to emergence of the Alpha variant was strict due to limited testing capacity, potentially leading to an ascertainment bias towards more severe cases in the first wave. This makes it even more striking that the association of the Alpha variant with severe disease is so prominent.

Notably the only other published study in hospital cohorts showed no difference in severity as measured by the composite outcome of need for ventilation or death [10]. Broadly the two cohorts from these hospital cohorts are similar, with an average age of around 60 and a high proportion of non-white ethnicities. In general this supports the external validity of our findings, however replication in dissimilar cohorts are awaited. Interestingly, despite male sex being widely reported to be a risk factor for severe disease our multivariate model confirms findings by these authors that sex is not significantly associated with severity in hospitalised cohorts after adjusted analysis.

The lack of association between severity and male sex may corresponds to the increase in the proportion of females in the admitted cohort of wave two and those infected with Alpha, accounting for an extra 5% of admissions with SARS-CoV-2 infection. Unpublished data referenced by NERVTAG [21] suggests the Alpha variant may be more severe in hospitalised females, who may have increased mortality or requirement for ICU care. Our data, showing an increase in the proportion of females in the admission cohort and lack of expected association of severity with male sex is consistent with the finding that Alpha may show increased virulence in females.

We also included an assessment of bias by comparing characteristics of non-sequenced cases with those successfully sequenced. We found no significant difference between the proportion with the outcome measure of hypoxia on admission between our sequenced and non-sequenced cases, suggesting no significant bias towards severity in the sequenced group, which was predominantly made up of cases of the Alpha variant.

For peer review only

Admitted cases in wave two were also around half as likely to have a diagnosis of frailty, which may be due to fewer admissions from care homes during wave two, which has been reported both nationally [22] and internationally [23]. Additionally, admitted cases were around a third less likely to have cancer in wave two. Both of these reductions may also be as a result of individuals shielding, and therefore at reduced risk for acquiring SARS-CoV-2 infection. Other differences in comorbidities between waves were small and of unclear clinical significance.

One additional striking observation was the similarity in number of nosocomial cases in wave one (n=96 of 934 [10%] inpatient cases) and wave two (n=137 of 1640 [8%] inpatient cases). Interestingly, nosocomial cases in wave one increased and started to fall before impact of the main infection control interventions of banning hospital visitors (March 25th), introducing universal surgical mask wearing (28th March 2020) and universal regular inpatient screening (after the first wave). In comparison, all these measures were in place prior to the second wave. The similar number of cases in wave two may in part be due to increased inpatient screening, which would identify asymptomatic cases, or introduction of the more transmissible Alpha variant which made up the vast majority of our sequenced nosocomial cases.

Some healthcare institutions report far fewer nosocomial acquisitions; for instance an academic hospital in Boston, USA reported only 2 nosocomial cases in over 9000 admissions [24]. This could be due to greater availability of side rooms for isolation or their use of N95 masks by HCWs, which may decrease transmission between HCWs and patients. In contrast, current UK public health policy recommends surgical facemasks for patient interactions unless performing aerosol generating procedures [34]. This incidence of nosocomial infection is a major challenge for UK healthcare institutions, with associated crude mortality at around 30% during the first wave [25,26]. For this reason it will be important to further investigate the factors involved in nosocomial acquisition in both waves.

For peer review only

Our study population comes from one city and therefore needs to be compared with findings in other regions. Our dataset included cases confirmed by SARS-CoV-2 RNA testing in our laboratory, so may miss those diagnosed only clinically. A follow-up study is needed to assess the effect of the Alpha variant on mortality, when the wave two cohort has completed hospital stay.

The number of cases diagnosed, admissions and nosocomial cases were higher in wave two than wave one, likely due to the increased incidence caused by the more transmissible Alpha variant. Infection with the Alpha variant was associated with severity as measured by hypoxic on admission, the first such finding in hospitalised cohorts.

For peer review only

Contributions

LBS and WW were involved in the conceptualization, methodology, formal analysis of the synthesised data and writing (original drafting, review, and editing). TC, AA-M and GN were involved in investigation being responsible for whole genome sequencing and analysis of results. RB, FH and LdJ were involved in resources, administration and data curation. YW, JE, VC were involved in supervision, funding acquisition and drafting (review and editing). All authors agreed the final manuscript.

For peer review only

References

1. Edelstein M, Obi C, Chand M, Hopkins S, Brown K, Ramsay M. SARS-CoV-2 infection in London, England: changes to community point prevalence around lockdown time, March–May 2020. J Epidemiol Community Health. 2020 [cited 10 Dec 2020]. doi:10.1136/jech-2020-214730

2. Official UK Coronavirus Dashboard. [cited 1 Mar 2021]. Available: https://coronavirus.data.gov.uk/details/healthcare

3. Kirby T. New variant of SARS-CoV-2 in UK causes surge of COVID-19. Lancet Respir Med. 2021;9: e20–e21.

4. Preliminary genomic characterisation of an emergent SARS-CoV-2 lineage in the UK defined by a novel set of spike mutations. 18 Dec 2020 [cited 24 Jan 2021]. Available: https://virological.org/t/preliminary-genomic-characterisation-of-an-emergent-sars-cov-2-lineage-in-the-uk-defined-by-a-novel-set-of-spike-mutations/563

5. Sparrow A, Carroll R, Pidd H. UK Covid: Boris Johnson says “great strides” made with vaccinations but many are yet to be reached – as it happened. The Guardian. 10 Feb 2021. Available: http://www.theguardian.com/politics/live/2021/feb/10/uk-covid-news-shapps-summer-holidays-abroad-travel-coronavirus-vaccine-live-latest-updates. Accessed 1 Mar

2021.

6. Chand M, Others. Investigation of novel SARS-COV-2 variant: Variant of Concern 202012/01 (PDF). Public Health England PHE. 2020.

7. Volz E, Mishra S, Chand M, Barrett JC, Johnson R, Geidelberg L, et al. Transmission of SARS-CoV-2 Lineage B.1.1.7 in England: Insights from linking epidemiological and genetic data. doi:10.1101/2020.12.30.20249034

8. Davies NG, Jarvis CI, John Edmunds W, Jewell NP, Diaz-Ordaz K, Keogh RH. Increased mortality in community-tested cases of SARS-CoV-2 lineage B.1.1.7. Nature. 2021;593: 270–274.

9. Challen R, Brooks-Pollock E, Read JM, Dyson L, Tsaneva-Atanasova K, Danon L. Risk of mortality in patients infected with SARS-CoV-2 variant of concern 202012/1: matched cohort study. BMJ. 2021;372. doi:10.1136/bmj.n579

10. Frampton D, Rampling T, Cross A, Bailey H, Heaney J, Byott M, et al. Genomic characteristics and clinical effect of the emergent SARS-CoV-2 B.1.1.7 lineage in London, UK: a whole-genome sequencing and hospital-based cohort study. Lancet Infect Dis. 2021. doi:10.1016/S1473-3099(21)00170-5

11. Surveillance definitions for COVID-19. [cited 24 Jan 2021]. Available: https://www.ecdc.europa.eu/en/covid-19/surveillance/surveillance-definitions

12. Quick J. nCoV-2019 sequencing protocol v1 (protocols.io.bbmuik6w). protocols.io. ZappyLab, Inc.; 2020. doi:10.17504/protocols.io.bbmuik6w

13. Artic Network. [cited 24 Jan 2021]. Available: https://artic.network/ncov-2019/ncov2019-bioinformatics-sop.html

14. pangolin. Github; Available: https://github.com/cov-lineages/pangolin

15. e-research. Acknowledging - Rosalind research computing infrastructure. [cited 6 Mar 2021].

For peer review only

Available: https://rosalind.kcl.ac.uk/acknowledging/

16. Saito S, Asai Y, Matsunaga N, Hayakawa K, Terada M, Ohtsu H, et al. First and second COVID-19 waves in Japan: A comparison of disease severity and characteristics. Journal of Infection. 2020. doi:10.1016/j.jinf.2020.10.033

17. White E. Ethnicity and National Identity in England and Wales - Office for National Statistics. Office for National Statistics; 11 Dec 2012 [cited 18 Jun 2021]. Available: https://www.ons.gov.uk/peoplepopulationandcommunity/culturalidentity/ethnicity/articles/ethnicityandnationalidentityinenglandandwales/2012-12-11

18. Nyberg T, Twohig KA, Harris RJ, Seaman SR, Flannagan J, Allen H, et al. Risk of hospital admission for patients with SARS-CoV-2 variant B.1.1.7: cohort analysis. BMJ. 2021;373. doi:10.1136/bmj.n1412

19. Kara Steel And. Coronavirus (COVID-19) Infection Survey, UK - Office for National Statistics. Office for National Statistics; 8 Jan 2021 [cited 1 Feb 2021]. Available: https://www.ons.gov.uk/peoplepopulationandcommunity/healthandsocialcare/conditionsanddiseases/bulletins/coronaviruscovid19infectionsurveypilot/8january2021

20. England NHS. NHS England » Hospitals admit one third of COVID patients in a single month. [cited 1 Mar 2021]. Available: https://www.england.nhs.uk/2021/02/hospitals-admit-one-third-of-covid-patients-in-a-single-month/

21. Scientific Advisory Group for Emergencies. NERVTAG: Update note on B.1.1.7 severity, 11 February 2021. GOV.UK; 12 Feb 2021 [cited 5 Mar 2021]. Available: https://www.gov.uk/government/publications/nervtag-update-note-on-b117-severity-11-february-2021

22. John S. Deaths involving COVID-19 in the care sector, England and Wales - Office for National Statistics. Office for National Statistics; 2 Jul 2020 [cited 25 Feb 2021]. Available: https://www.ons.gov.uk/peoplepopulationandcommunity/birthsdeathsandmarriages/deaths/articles/deathsinvolvingcovid19inthecaresectorenglandandwales/deathsoccurringupto12june2020

For peer review only

andregisteredupto20june2020provisional

23. Ioannidis JPA, Axfors C, Contopoulos-Ioannidis DG. Second versus first wave of COVID-19 deaths: shifts in age distribution and in nursing home fatalities. medRxiv. 2020; 2020.11.28.20240366.

24. Rhee C, Baker M, Vaidya V, Tucker R, Resnick A, Morris CA, et al. Incidence of Nosocomial COVID-19 in Patients Hospitalized at a Large US Academic Medical Center. JAMA Netw Open. 2020;3: e2020498.

25. Carter B, Collins JT, Barlow-Pay F, Rickard F, Bruce E, Verduri A, et al. Nosocomial COVID-19 infection: examining the risk of mortality. The COPE-Nosocomial Study (COVID in Older PEople). J Hosp Infect. 2020;106: 376–384.

26. Snell LB, Fisher CL, Taj U, Merrick B, Alcolea-Medina A, Charalampous T, et al. Combined epidemiological and genomic analysis of nosocomial SARS-CoV-2 transmission identifies community social distancing as the dominant intervention reducing outbreaks. bioRxiv. medRxiv; 2020. doi:10.1101/2020.11.17.20232827

34. Public Health England. COVID-19: infection prevention and control (IPC). GOV.UK; 10 Jan 2020 [cited 15 Mar 2021]. Available: https://www.gov.uk/government/publications/wuhan-novel-coronavirus-infection-prevention-and-control

35. Carter B, Collins JT, Barlow-Pay F, Rickard F, Bruce E, Verduri A, et al. Nosocomial COVID-19 infection: examining the risk of mortality. The COPE-Nosocomial Study (COVID in Older PEople). J Hosp Infect. 2020;106: 376–384. doi:10.1016/j.jhin.2020.07.013

36. Snell LB, Fisher CL, Taj U, Merrick B, Alcolea-Medina A, Charalampous T, et al. Combined epidemiological and genomic analysis of nosocomial SARS-CoV-2 transmission identifies community social distancing as the dominant intervention reducing outbreaks. bioRxiv. medRxiv; 2020. doi:10.1101/2020.11.17.20232827

For peer review only

For peer review only

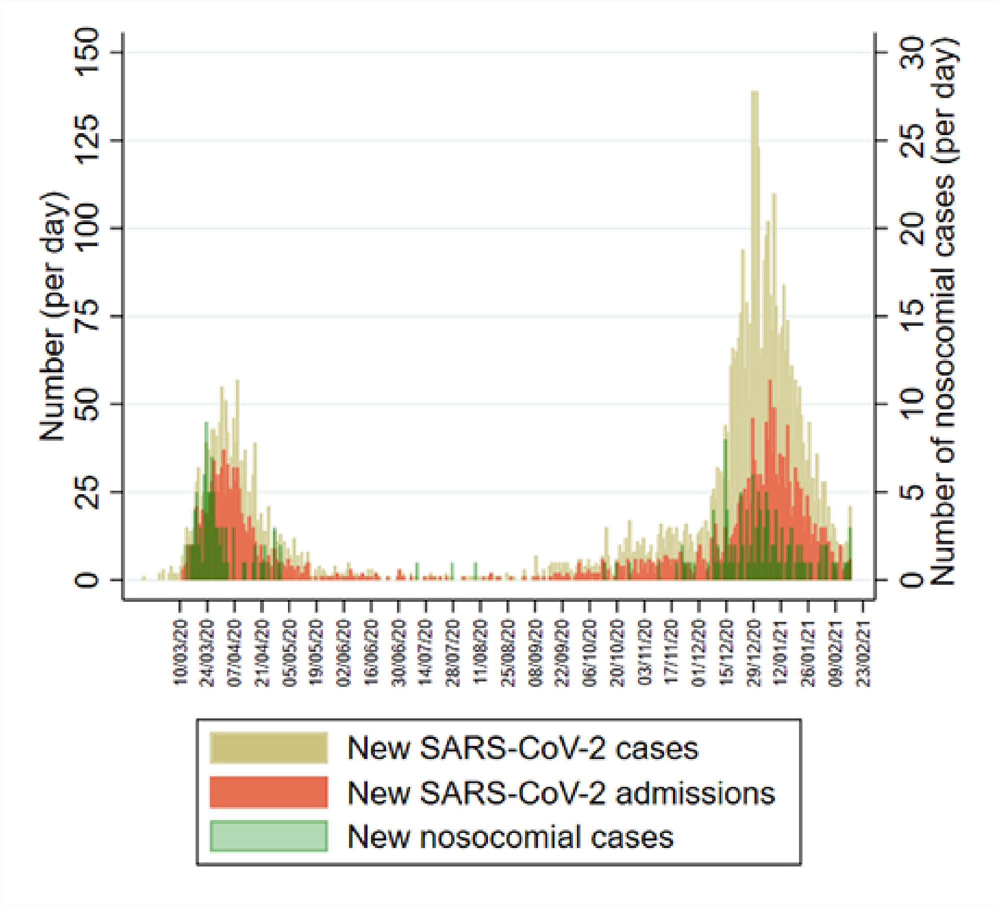

Distribution of laboratory confirmed SARS-CoV-2 cases over time. Daily incidence of new cases (beige), newly admitted cases (orange) and nosocomial acquisitions (green) over time.

190x173mm (72 x 72 DPI)

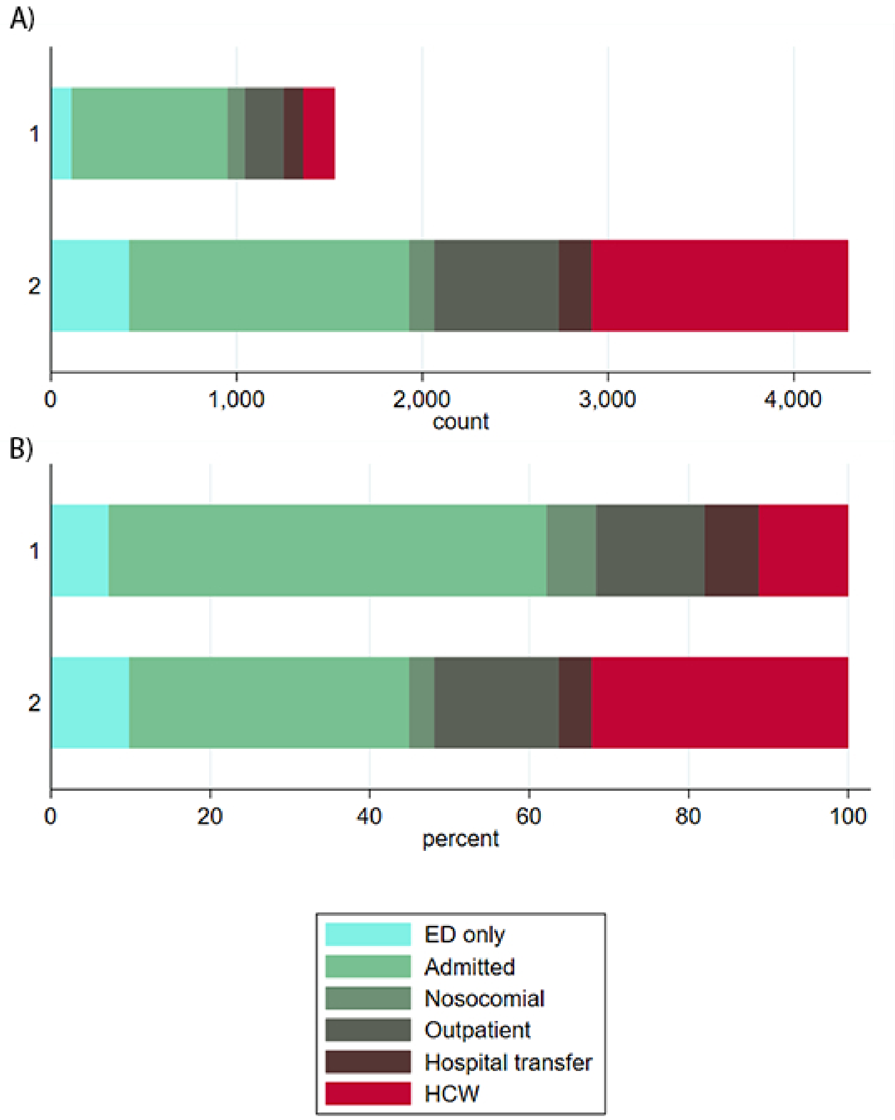

(A) Absolute number of cases within the different hospital cohorts during wave one (upper) and wave two (lower). (B) Proportion of cases within the different hospital cohorts during wave one (upper) and wave two (lower).

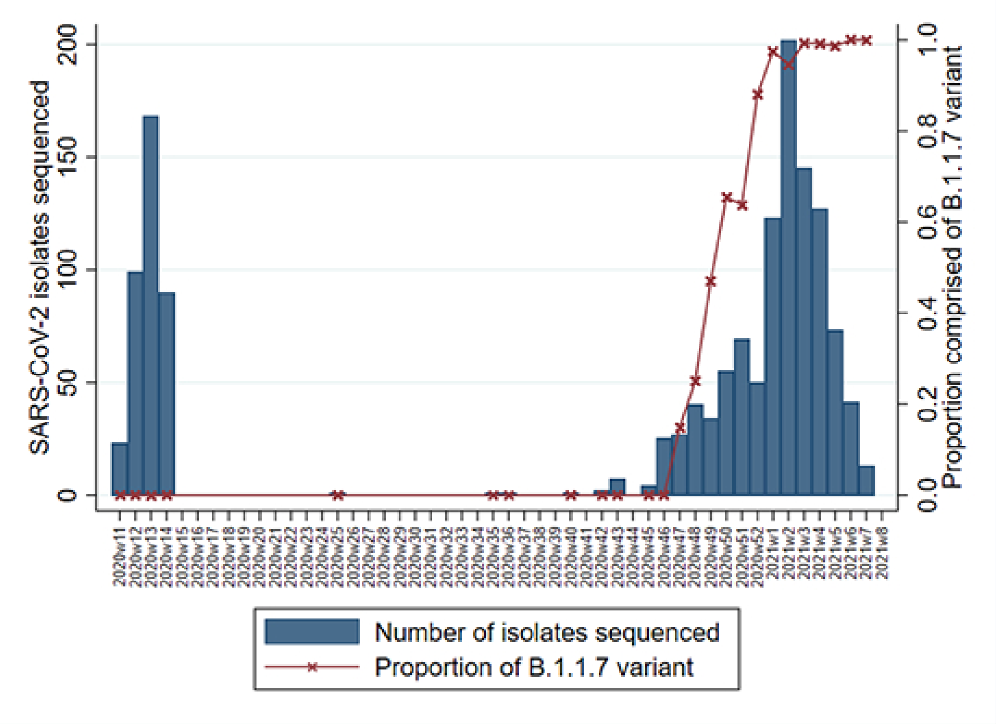

Number of cases with sequenced SARS-CoV-2 isolates by epi-week (bar) and the proportion of which were made up of the alpha variant B.1.1.7 (red line)

190x138mm (72 x 72 DPI)

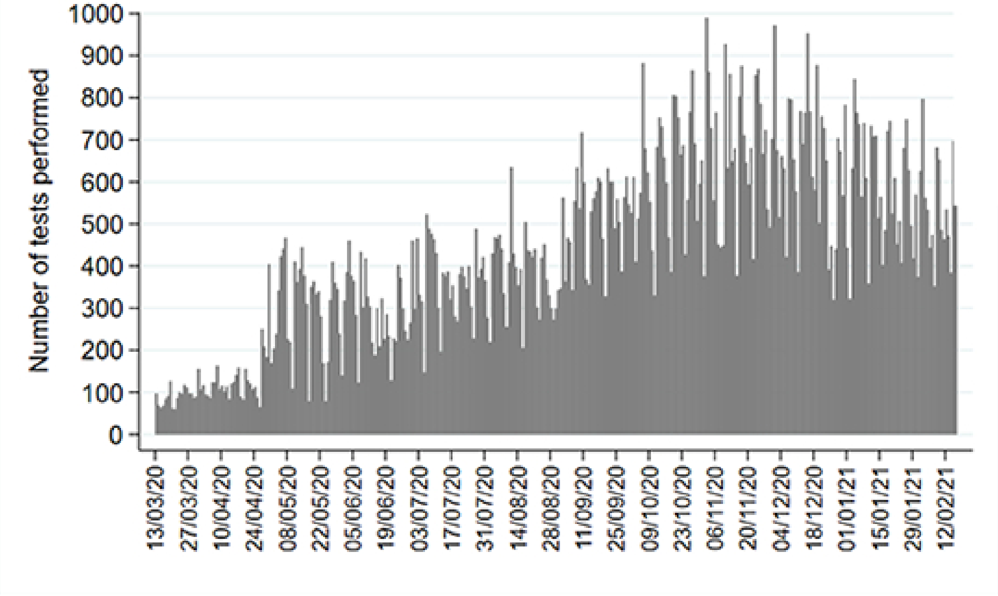

Our laboratory began testing on 13th March 2020 with initial capacity for around 150 PCR tests per day, before increasing to around 500 tests per day in late April during wave one, and up to 1000 tests per day during wave two

190x125mm (72 x 72 DPI)

STROBE 2007 (v4) Statement—Checklist of items that should be included in reports of *cohort studies*

Section/Topic	Item #	Recommendation	Reported on page #
**Title and abstract**	1	(*a*) Indicate the study's design with a commonly used term in the title or the abstract	2
		(*b*) Provide in the abstract an informative and balanced summary of what was done and what was found	2
**Introduction**			
Background/rationale	2	Explain the scientific background and rationale for the investigation being reported	6
Objectives	3	State specific objectives, including any prespecified hypotheses	6
**Methods**			
Study design	4	Present key elements of study design early in the paper	7-9
Setting	5	Describe the setting, locations, and relevant dates, including periods of recruitment, exposure, follow-up, and data collection	7-9
Participants	6	(*a*) Give the eligibility criteria, and the sources and methods of selection of participants. Describe methods of follow-up	8
		(*b*) For matched studies, give matching criteria and number of exposed and unexposed	NA
Variables	7	Clearly define all outcomes, exposures, predictors, potential confounders, and effect modifiers. Give diagnostic criteria, if applicable	7-9
Data sources/ measurement	8*	For each variable of interest, give sources of data and details of methods of assessment (measurement). Describe comparability of assessment methods if there is more than one group	8-9
Bias	9	Describe any efforts to address potential sources of bias	15
Study size	10	Explain how the study size was arrived at	12
Quantitative variables	11	Explain how quantitative variables were handled in the analyses. If applicable, describe which groupings were chosen and why	8-9
Statistical methods	12	(*a*) Describe all statistical methods, including those used to control for confounding	9-10
		(*b*) Describe any methods used to examine subgroups and interactions	NA
		(*c*) Explain how missing data were addressed	9
		(*d*) If applicable, explain how loss to follow-up was addressed	NA
		(*e*) Describe any sensitivity analyses	NA
**Results**			

For peer review only

Participants	13*	(a) Report numbers of individuals at each stage of study—eg numbers potentially eligible, examined for eligibility, confirmed eligible, included in the study, completing follow-up, and analysed	10-12
		(b) Give reasons for non-participation at each stage	15
		(c) Consider use of a flow diagram	Not done
Descriptive data	14*	(a) Give characteristics of study participants (eg demographic, clinical, social) and information on exposures and potential confounders	15-18
		(b) Indicate number of participants with missing data for each variable of interest	In tables
		(c) Summarise follow-up time (eg, average and total amount)	NA
Outcome data	15*	Report numbers of outcome events or summary measures over time	Page 18<br>Table 1, Table 2
Main results	16	(*a*) Give unadjusted estimates and, if applicable, confounder-adjusted estimates and their precision (eg, 95% confidence interval). Make clear which confounders were adjusted for and why they were included	12-18<br>Table 1,2
		(*b*) Report category boundaries when continuous variables were categorized	NA
		(*c*) If relevant, consider translating estimates of relative risk into absolute risk for a meaningful time period	NA
Other analyses	17	Report other analyses done—eg analyses of subgroups and interactions, and sensitivity analyses	None performed
**Discussion**			
Key results	18	Summarise key results with reference to study objectives	22
**Limitations**			
Interpretation	20	Give a cautious overall interpretation of results considering objectives, limitations, multiplicity of analyses, results from similar studies, and other relevant evidence	22-23
Generalisability	21	Discuss the generalisability (external validity) of the study results	23
**Other information**			
Funding	22	Give the source of funding and the role of the funders for the present study and, if applicable, for the original study on which the present article is based	4

*Give information separately for cases and controls in case-control studies and, if applicable, for exposed and unexposed groups in cohort and cross-sectional studies.

Note: An Explanation and Elaboration article discusses each checklist item and gives methodological background and published examples of transparent reporting. The STROBE checklist is best used in conjunction with this article (freely available on the Web sites of PLoS Medicine at http://www.plosmedicine.org/, Annals of Internal Medicine at http://www.annals.org/, and Epidemiology at http://www.epidem.com/). Information on the STROBE Initiative is available at www.strobe-statement.org.

For peer review only

BMJ Open

A descriptive comparison of admission characteristics between pandemic waves and multivariable analysis of the association of the Alpha variant with disease severity in inner London.

Journal:	*BMJ Open*
Manuscript ID	bmjopen-2021-055474.R1
Article Type:	Original research
Date Submitted by the Author:	16-Sep-2021

Complete List of Authors:	Snell, Luke; King's College London; Guy's and St Thomas' NHS Foundation Trust<br>Wang , Wenjuan; King's College London<br>Medina, Adela; Viapath; King's College London<br>Charalampous, Themoula; King's College London<br>Nebbia, Gaia; King's College London; Guy's and St Thomas' NHS Foundation Trust<br>Batra, Rahul; King's College London<br>de Jongh, Leonardo; Guy's and St Thomas' NHS Foundation Trust<br>Higgins, Finola; Guy's and St Thomas' NHS Foundation Trust<br>Wang, Yanzhong; King's College London<br>Edgeworth, Jonathan; King's College London; Guy's and St Thomas' NHS Foundation Trust<br>Curcin, Vasa; King's College London

**Primary Subject Heading**:	Infectious diseases
Secondary Subject Heading:	Epidemiology
Keywords:	VIROLOGY, EPIDEMIOLOGY, Epidemiology < INFECTIOUS DISEASES, Public health < INFECTIOUS DISEASES

I, the Submitting Author has the right to grant and does grant on behalf of all authors of the Work (as defined in the below author licence), an exclusive licence and/or a non-exclusive licence for contributions from authors who are: i) UK Crown employees; ii) where BMJ has agreed a CC-BY licence shall apply, and/or iii) in accordance with the terms applicable for US Federal Government officers or employees acting as part of their official duties; on a worldwide, perpetual, irrevocable, royalty-free basis to BMJ Publishing Group Ltd ("BMJ") its licensees and where the relevant Journal is co-owned by BMJ to the co-owners of the Journal, to publish the Work in this journal and any other BMJ products and to exploit all rights, as set out in our licence.

The Submitting Author accepts and understands that any supply made under these terms is made by BMJ to the Submitting Author unless you are acting as an employee on behalf of your employer or a postgraduate student of an affiliated institution which is paying any applicable article publishing charge ("APC") for Open Access articles. Where the Submitting Author wishes to make the Work available on an Open Access basis (and intends to pay the relevant APC), the terms of reuse of such Open Access shall be governed by a Creative Commons licence – details of these licences and which Creative Commons licence will apply to this Work are set out in our licence referred to above.

Other than as permitted in any relevant BMJ Author's Self Archiving Policies, I confirm this Work has not been accepted for publication elsewhere, is not being considered for publication elsewhere and does not duplicate material already published. I confirm all authors consent to publication of this Work and authorise the granting of this licence.

For peer review only

A descriptive comparison of admission characteristics between pandemic waves and multivariable analysis of the association of the Alpha variant with disease severity in inner London.

Luke B.Snell MBBS*, Wenjuan Wang PhD*, Adela Alcolea-Medina MPhil, Themoula Charalampous PhD, Gaia Nebbia PhD, Rahul Batra MD, Leonardo de Jongh, Finola Higgins, Yanzhong Wang PhD, Jonathan D. Edgeworth PhD♯, Vasa Curcin PhD♯

1 King's College London, London, UK (L.B. Snell, W. Wang, A. Alcolea-Medina, T. Charalampous, G. Nebbia, R. Batra, Y. Wang, J.D. Edgeworth, V Curin)
2 Guy's and St Thomas' NHS Foundation Trust, London, UK (L.B. Snell, G. Nebbia, R. Batra, L. de Jongh, F. Higgins, J.D. Edgeworth)
3 Viapath, St Thomas' Hospital, London, UK (A. Alcolea-Medina)

* These first authors contributed equally

♯ These authors were co–principal investigators

Corresponding author: Dr. Luke B Snell, St. Thomas' Hospital, Westminster Bridge Road, UK SE1 7EH, luke.snell@nhs.net, 02071887188

Running title: Comparison of SARS-CoV-2 waves and alpha variant severity

Article word count: 3714

For peer review only

Abstract

Background. The Alpha variant emerged and became the dominant circulating variant in the UK in late 2020. Current literature is unclear on whether the Alpha variant is associated with increased severity. We linked clinical data with viral genome sequence data to compare admitted cases between SARS-CoV-2 waves in London, and to investigate the association between Alpha variant and the severity of disease.

Methods. Clinical, demographic, laboratory and viral sequence data from electronic health record (EHR) systems was collected for all cases with a positive SARS-CoV-2 RNA test between March 13th 2020 and February 17th 2021 in a multi-site London healthcare institution. Multivariate analysis using logistic regression assessed risk factors for severity as defined by hypoxia at admission.

Results There were 5810 SARS-CoV-2 RNA positive cases of which 2341 were admitted (838 in wave one and 1503 in wave two). Both waves had a temporally aligned rise in nosocomial cases (96 in wave one, 137 in wave two). The Alpha variant was first identified on 15th November 2020 and increased rapidly to comprise 400/472 (85%) of sequenced isolates from admitted cases in wave two. A multivariate analysis identified risk factors for severity on admission, such as age (OR 1.02 [CI 1.01-1.03] for every year older, p<0.001), obesity (OR 1.7 [CI 1.28-2.26], p<0.001) and infection with the Alpha variant (OR 1.68 [CI 1.26-2.24], p<0.001).

Conclusions Our analysis is the first in hospitalised cohorts to show increased severity of disease associated with the Alpha variant. The number of nosocomial cases was similar in both waves despite the introduction of many infection control interventions before wave two.

Abstract word count: 250

For peer review only

Strengths and limitations of this study

- Published evidence on whether the alpha variant of SARS-CoV-2 has a higher case fatality is mixed.
- Our study benefits from a long study window, including patients since the beginning of the SARS-CoV-2 pandemic.
- We combine patient-level data with routinely performed genomic data, capturing a large proportion of inpatients with confirmed SARS-CoV-2 infection.
- Our analysis adjusts for comorbidities, a feature missing from many of the population-level studies currently published.

For peer review only

Ethics

Ethical approval for data informatics was granted by The London Bromley Research Ethics Committee (reference (20/HRA/1871)) to the King's Health Partners Data Analytics and Modelling COVID-19 Group to collect clinically relevant data points from patients' electronic health records.

Whole genome sequencing of residual viral isolates was conducted under the COVID-19 Genomics UK (COG-UK) consortium study protocol, which was approved by the Public Health England Research Ethics and Governance Group (reference: R&D NR0195).

Acknowledgements

The authors acknowledge use of the research computing facility at King's College London, Rosalind (https://rosalind.kcl.ac.uk), which is delivered in partnership with the National Institute for Health Research (NIHR) Biomedical Research Centres at South London & Maudsley and Guy's & St. Thomas' NHS Foundation Trusts, and part-funded by capital equipment grants from the Maudsley Charity (award 980) and Guy's & St. Thomas' Charity (TR130505). The views expressed are those of the author(s) and not necessarily those of the NHS, the NIHR, King's College London, or the Department of Health and Social Care.

Funding

FH, LBS, YW, and VC are supported by the National Institute for Health Research (NIHR) Biomedical Research Centre programme of Infection and Immunity (RJ112/N027) based at Guy's and St Thomas' National Health Service NHS) Foundation Trust and King's College London.

This work was also supported by The Health Foundation and the Guy's and St Thomas' Charity.

COG-UK is supported by funding from the Medical Research Council (MRC) part of UK Research & Innovation (UKRI), the National Institute of Health Research (NIHR) and Genome Research Limited, operating as the Wellcome Sanger Institute.

For peer review only

Data sharing

Genomic data is publicly available on GISAID, please contact the authors for accession numbers. Our ethical approval precludes public sharing of patient-level data.

Patient and public involvement

As this study was conducted to meet an urgent public health need, patient and public involvement was not sought

Competing interests

None to declare.

For peer review only

Background

SARS-CoV-2 infection has led to the death of over 1 million individuals worldwide since its emergence in China during December 2019, with over 120,000 deaths reported in the UK as of July 2021. In London, the estimated incidence of new cases in the first wave peaked around March 23rd 2020 at 2.2% [1] and then rapidly declined following non-pharmacological interventions. Hospital admissions peaked about 1 week later [2] reflecting the median period of symptoms before hospital presentation. A “second wave” of infections started in London around the beginning of October 2020 [3].

Genome sequencing identified the Alpha variant (the B.1.1.7 lineage) around the South East of England, which spread rapidly as part of the emerging second wave [4]. This occurred prior to widespread vaccination, with only 25% of the adult population receiving the first dose by mid-February 2021 [5]. The Alpha variant has been associated with increased transmissibility in community studies [6] [7], and community studies associate the variant with increased mortality [8] [9]. However, the single published study in hospitalised patients suggested no increase in need for ventilation or mortality [10].

Changes in transmissibility and severity have the potential to affect the burden on healthcare systems, and modify the characteristics of cases presenting to hospitals including the demographics, comorbidities and severity of disease associated with SARS-CoV-2 infection.

Objectives

We linked clinical datasets with local SARS-CoV-2 variant analysis to compare admission characteristics of hospitalised cases during the two waves of infection and to look at the association of the alpha variant with the severity of disease at presentation to hospital.

For peer review only

Methods

Setting

Guy's and St Thomas' NHS Foundation Trust (GSTT) is a multi-site healthcare institution providing general and emergency services predominantly to the South London boroughs of Lambeth and Southwark. An acute-admitting site (St Thomas' Hospital) has an adult emergency department, with a large critical care service including one of the UK's eight nationally commissioned extra corporeal membrane oxygenation (ECMO) centres for severe respiratory failure. A second site (Guy's Hospital) provides more inpatient services such as elective surgery, cancer care and other specialist services. A paediatric hospital (Evelina London) acts as a general and specialised referral centre. Several satellite sites for specialist services like dialysis, rehabilitation and long term care are also part of the institution. GSTT receives patients from regional hospitals predominantly to critical care through 'mutual aid' schemes.

SARS-CoV-2 laboratory testing

Our laboratory began testing on 13th March 2020 with initial capacity for around 150 PCR tests per day, before increasing to around 500 tests per day in late April during wave one, and up to 1000 tests per day during wave two (Supplementary Figure 1).

Testing commenced during the first wave on 13th March 2020 limited to cases requiring admission or inpatients who had symptoms of fever or cough, as per national recommendation; guidance suggested cases which did not require admission should not be tested. For wave two, all cases admitted to hospital were screened and underwent universal interval screening at varying time points. Staff testing for symptomatic healthcare workers was also introduced towards the end of wave one. Comparative analysis was therefore restricted to SARS-CoV-2 RNA positive cases requiring admission. Cases without laboratory confirmation of SARS-CoV-2 infection were not included.

For peer review only

Assays used for the detection of SARS-CoV-2 RNA include PCR testing using Aus Diagnostics or by the Hologic Aptima SARS-CoV-2 Assay. Nucleic acid was first extracted using the QIAGEN QIAsymphony SP system and a QIAsymphony DSP Virus/Pathogen Mini Kit (catalogue No: 937036) with the off-board lysis protocol.

Definitions and participants

Cases were identified by the first positive SARS-CoV-2 RNA test. Cases were placed in mutually exclusive categories with the following definitions: 1) outpatients 2) testing through occupational health 3) emergency department attenders not subsequently admitted within 14 days 4) patients admitted within 14 days of a positive test 5) nosocomial cases, defined based on ECDC definitions, as those having a first positive test on day 8 or later after admission to hospital where COVID-19 was not suspected on admission [11] and 6) interhospital transfers.

For the purpose of comparison, only the inpatient group admitted within 14 days following a positive test, were taken forward for onward comparison. This methodology of only including admissions was adopted to prevent increased testing during the pandemic affecting case ascertainment and biasing severity of cases. This is evidenced in Supplementary Figure 1, with tests increasing steadily from 100 per day to more than 1000 per day. Additionally, in wave two more interhospital transfers of severe cases requiring extracorporeal membrane oxygenation were received, mostly several days after admission. This category of patients were therefore excluded from analysis to prevent biasing towards severe disease.

A composite datapoint for 'hypoxia' was created, equivalent to WHO ordinal scale of ≥4 [12], with cases taken to be hypoxic if on admission they had oxygen saturations of <94%, if they were recorded as requiring supplemental oxygen, or if the fraction of inspired oxygen was recorded as being greater than 0.21.

For peer review only

Determination of SARS-CoV-2 lineage

Whole genome sequencing of residual samples from SARS-CoV-2 cases was performed using GridION (Oxford Nanopore Technology), using version 3 of the ARTIC protocol [13] and bioinformatics pipeline [14]. Samples were selected for sequencing if the corrected CT value was 33 or below, or the Hologic Aptima assay was above 1000 RLU. During the first wave, sequencing occurred between March 1st - 31st, whilst sequencing in the second wave restarted in November 2020 - March 2021. Variants were called using updated versions of pangolin 2.0 [15]. We considered all cases in wave one to be non-Alpha variants, as our wave one cut-off of 25th July 2020 was 6 weeks prior to first identified cases of the Alpha variant in the UK [16] and before Alpha variant was first identified in our population in November 2020.

Data sources, extraction and integration

Clinical, laboratory and demographic data for all cases with a laboratory reported SARS-CoV-2 PCR RNA test on nose and throat swabs or lower respiratory tract specimens were extracted from hospital electronic health record (EHR) data sources using records closest to the test date. Data were linked to the Index of Multiple Deprivation (IMD). Age, sex and ethnicity were extracted from EPR. Self-reported ONS ethnic categories were stratified into White (British, Irish, Gypsy and White-Other) or non-White (Black [African, Caribbean, and Black-Other], Asian [Bangladeshi, Chinese, Indian, Pakistan, and Asian-Other] and Mixed/Other). Numbers for which data were missing is listed by each variable. Comorbidities and medication history were extracted from the EPR and e-Noting using natural language processing (NLP). If a comorbidity was not recorded it was assumed not to be present. Cases were characterised as having/not having a past medical history of hypertension, cardiovascular disease (stroke, transient ischaemic attack, atrial fibrillation, congestive heart failure, ischaemic heart disease, peripheral artery disease or atherosclerotic disease), diabetes mellitus, chronic kidney disease, chronic respiratory disease (chronic obstructive pulmonary disease, asthma, bronchiectasis or pulmonary fibrosis) and neoplastic disease (solid tumours, haematological neoplasias or metastatic disease). Obesity was defined as either obesity present in the notes, or recorded Body Mass Index (BMI) >= 30 kg/m^2. Medicines data were extracted using both structured

For peer review only

queries and natural language processing tools with medical and drug dictionaries .Additionally, checks on free text data were performed by a cardiovascular clinician to ensure the information was accurate.

Analysis was carried out on the secure Rosalind high-performance computer infrastructure [17] running Jupyter Notebook 6.0.3, R 3.6.3 and Python 3.7.6.

Statistical analysis and outcome measures

Descriptive statistics were summarised with mean and standard deviation (SD) for continuous variables if the distribution is normal and median and interquartile range (IQR) if the distribution is non-normal. Count and percentages were used for categorical variables. For the comparisons of variables for wave one versus wave two variables, Alpha variant versus non-Alpha variants, as well as sequenced patients versus non-sequenced patients in wave two, Kruskal-Walllis test was used for continuous variables and Chi-squared test for categorical variables with significance level of p=0.05. Multivariate analysis was performed using logistic regression to assess the odds ratios of different risk factors (including age, sex, ethnicity (White, non-White, Unknown), variant status (Alpha or non-Alpha), and cardiovascular disease, hypertension, diabetes, chronic respiratory disease, cancer, kidney disease, HIV, transplant, and frailty) for hypoxia on admission as the binary outcome indicating severity at admission. Cases with missing datapoints were dropped from. Variables to be included in the multivariate analysis. were chosen by literature review and expert opinion (see Supplementary Material).

For peer review only

Results

General epidemiology and results of viral sequencing.

Figure 1 shows the incidence of SARS-CoV-2 cases, SARS-CoV-2 admissions, and nosocomial cases since March 13th 2020. In total 5810 individuals had a positive SARS-CoV-2 PCR test up until the data extraction date of 17th February 2021. Two "waves" are evident with July 25th taken as a separation date between waves, at which point a minimum of 12 wave one cases remained in hospital. Wave one comprised 1528 cases (26.3%) from when laboratory testing commenced on March 13th to peak rapidly between the 1st and 8th April 2020 with 57 new cases, before falling to a baseline by May 12th 2020. Ninety-one percent (1391/1528) of all cases in wave one occurred during these 60 days. Wave two comprised 4282 cases (73.7%), with incidence first increasing gradually from the beginning of October. There was then a period of rapidly escalating incidence from about 10th December, peaking on 28th December 2020 when 139 cases per day were diagnosed. 3446/4282 (80%) of wave two cases were detected during a comparable 60 day period between 10th December 2020 and 8th February 2021. In both waves, nosocomial cases peaked early, increasing along with admissions but then fell while the number of community admissions continued at peak levels.

Figure 1: Distribution of laboratory confirmed SARS-CoV-2 cases over time. Daily incidence of new cases (beige), newly admitted cases (orange) and nosocomial acquisitions (green) over time.

Individuals with a positive test were placed into six categories (Figure 2). The 5810 SARS-CoV-2 cases were categorised as follows, inpatients admitted within 14 days of a positive test (n=2341), healthcare workers (n=1549), outpatients (n=874), emergency department (ED) attenders not subsequently admitted (n=532), inter-hospital transfers (n=281) and nosocomial cases (n=233). Some observed differences between waves one and two reflected the increased availability of testing particularly for outpatients (208;13.6% v 666;15.6%), people sent home from ED (111;7.3% v 421; 9.8%) and healthcare workers (171;11.2% v 1378;32.2%). There were also more interhospital transfers of known COVID-19 cases in wave two (177;4.1% v 104;6.8% in wave one). In wave two,

For peer review only

the number of admissions increased (1503; 35.1% v 838; 54.8%) along with nosocomial cases (137;3.2% v 96;6.3%) compared with wave one.

Figure 2 (A) Absolute number of cases within the different hospital cohorts during wave one (upper) and wave two (lower). **(B)** Proportion of cases within the different hospital cohorts during wave one (upper) and wave two (lower).

Figure 3 shows the 1470 successfully sequenced SARS-CoV-2 isolates over time, with 382 from wave one and 1088 from wave two. Sequencing was successful for 216/838 (26%) admitted cases from wave one, 472/1503 (31%) admitted cases in wave two, and 121/233 (52%) nosocomial cases. The proportion of Alpha variant increased rapidly after the first Alpha isolate was identified on 15th November 2020, accounting for approximately two thirds within 3 weeks, and almost 100% (600/617 isolates, 97%) in January 2021. In the second wave, the Alpha variant made up 83% (908/1088) of all sequenced isolates, 85% (400/472) of sequenced isolates from admitted cases, and 88% (51/59) of sequenced isolates from nosocomial cases. In addition, two cases of the B.1.351 beta variant of concern were also detected in the wave two admission cohort.

Figure 3 Number of cases with sequenced SARS-CoV-2 isolates by epi-week (bar) and the proportion of which were made up of the alpha variant B.1.1.7 (red line)

Comparison of characteristics of admitted cases between waves one and two

Descriptive statistics of cases admitted during wave one (n=838) and wave two (n=1503) were compared (Table 1). There was a statistically significant difference in mean age of 2 years (62yrs in wave one v 60yrs in wave two, p=0.019), however admitted cases were more likely to be female in wave two (47.3% v 41.8%, p=0.011). A larger proportion of admitted cases in wave two were obese (29.1% v 24.6%, p=0.02). Comparison of comorbidities showed that those in wave two were less likely to have a diagnosis of frailty (11.5% v 22.8%, p<0.001), history of stroke (4.3% v 8.6%, p<0.001) or cancer (4.8% v 7.2%, p=0.022). There was no significant difference in proportion with

For peer review only

known comorbidities of diabetes, kidney disease, hypertension, cardiovascular disease or respiratory disease.

Table 1: Descriptive statistics of the cohort for wave one (n=838) and wave two (n=1503) admissions

	Missing	Wave one n (%)	Wave two n (%)	Wave one Median [IQR]	Wave two Median [IQR]	P-Value
Demographics						
Age	0			62.0 [49.0,78.0]	60.0 [47.0,74.0]	0.019
Male	0	488 (58.2)	792 (52.7)			0.011
Ethnicity	0					0.013
White		331 (39.5)	598 (39.8)			
Asian		64 (7.6)	121 (8.1)			
Black-African		177 (21.1)	262 (17.4)			
Black-Caribbean		73 (8.7)	98 (6.5)			
Mixed		15 (1.8)	18 (1.2)			
Other		45 (5.4)	107 (7.1)			
Unknown		133 (15.9)	299 (19.9)			
BMI	577			27.0 [23.8,31.7]	27.7 [24.0,32.9]	0.022
BMI>30		206 (24.6)	438 (29.1)			0.02
BMI>40		34 (4.1)	86 (5.7)			0.098
Physiological parameters						
Heart Rate	360			84.0 [75.0,94.0]	81.0 [72.0,91.0]	<0.001
Heart Rate>100		105 (12.5)	142 (9.4)			0.02
Blood pressure						
Systolic	369			125.0 [113.0,139.0]	127.0 [115.0,141.0]	0.013
Diastolic	369			73.0 [65.0,80.0]	75.0 [68.0,82.0]	<0.001
MAP	369			90.7 [82.2,99.0]	92.3 [84.7,101.3]	<0.001
Respiratory Rate	359			19.0 [18.0,22.0]	19.0 [18.0,22.0]	0.764
Respiratory Rate>20		200 (23.9)	365 (24.3)			0.86
Hypoxia	658	370 (64.3)	726 (65.5)			0.67
Temperature	361			36.9 [36.4,37.5]	36.6 [36.2,37.2]	<0.001
NEWS2	405					0.86
0		95 (11.3)	173 (11.5)			
1		108 (12.9)	192 (12.8)			
2		117 (14.0)	188 (12.5)			
2+		371 (44.3)	692 (46.0)			
Laboratory parameters						

For peer review only - http://bmjopen.bmj.com/site/about/guidelines.xhtml

Neutrophils	8			4.9 [3.4,7.6]	5.0 [3.3,7.5]	0.724
Lymphocytes	7			0.9 [0.6,1.3]	0.9 [0.6,1.4]	0.741
NLR	8			5.4 [3.1,9.9]	5.4 [3.2,9.8]	0.951
Creatinine	43			83.0 [64.0,115.0]	86.0 [68.0,117.0]	0.065
Urea	855			7.0 [4.6,12.2]	6.0 [4.3,9.9]	0.001
Estimated GFR	114			73.0 [48.0,98.0]	69.0 [48.0,89.0]	0.001
Albumin	185			37.0 [32.0,40.0]	38.0 [34.0,41.0]	<0.001
CRP	61			74.5 [26.0,148.0]	51.0 [18.0,103.8]	<0.001
DDimer	1297			1.1 [0.6,3.0]	0.9 [0.5,2.2]	0.001
Ferritin	905			855.0 [394.0,1533.5]	699.0 [342.0,1359.0]	0.05
Co-morbidities						
Stroke	0	72 (8.6)	64 (4.3)			<0.001
TIA	0	9 (1.1)	20 (1.3)			0.731
Hypertension	0	288 (34.4)	464 (30.9)			0.091
Diabetes	0	246 (29.4)	384 (25.5)			0.052
AF	0	63 (7.5)	115 (7.7)			0.972
IHD	0	146 (17.4)	244 (16.2)			0.495
Heart Failure	0	54 (6.4)	105 (7.0)			0.679
COPD	0	64 (7.6)	109 (7.3)			0.796
Asthma	0	74 (8.8)	138 (9.2)			0.835
Cancer	0	60 (7.2)	72 (4.8)			0.022
Kidney disease	0	112 (13.4)	181 (12.0)			0.389
HIV	0	21 (2.5)	36 (2.4)			0.979
Solid organ Transplant	0	24 (2.9)	49 (3.3)			0.686
Frailty	0	191 (22.8)	173 (11.5)			<0.001

†: p-value was from Kruskal-Wallis test for continuous variables and Chi-squared test for categorical variables.

Abbreviations: BMI: Body Mass Index, TIA: Transient Ischaemic Attack, IHD: Ischemic Heart Disease, COPD: Chronic Obstructive Pulmonary Disease, HIV: Human Immunodeficiency Virus, MAP:Mean Arterial Pressure, SPO2: Oxygen Saturation, GFR: Glomerular Filtration Rate, CRP: C-Reactive Protein, NLR: Neutrophils and Lymphocytes Ratio, NEWS2: National Early Warning Score 2.

For peer review only

There were no significant differences between waves in the proportion with severe SARS-CoV-2 disease upon admission as judged by hypoxia (64.3% in wave one vs 65.5% in wave two, p=0.67) or tachypnoea (respiratory rate>20) (23.9% vs 24.3%, p=0.86). There were small differences in other physiological parameters on admission, some of which reached statistical significance but differences were not clinically relevant.

Laboratory markers were compared between waves (Table 1). There were small but significant differences, such as lower CRP (median 51.0 mg/dL (IQR: 18.0-103.8) v 74.5 mg/dL (IQR: 26.0-148.0), p<0.001) and lower ferritin (699.0 [IQR: 342.0-1359.0] v 855.0 [IQR: 394.0-1533.5], p=0.05) in wave two. There were other small statistically significant differences without clear clinical significance, such as a lower D-Dimer in wave two (0.9 mg/L FEU [IQR: 0.5-2.2] v 1.1 mg/L FEU [IQR:0.6-3.0], p=0.001), and lower estimated GFR (69.0 ml/min [IQR: 48.0-89.0] v 73.0 ml/min [IQR: 48.0-98.0], p=0.001), lower urea (6.0 mmol/L [IQR: 4.3-9.3] v 7.0 mmol/L [IQR: 4.6-12.2], p=0.001) and higher albumin (38.0 g/L [IQR: 34.0-41.0 g/L] vs 37.0 g/L [IQR: 32.0-40.0], p<0.001). There was no significant difference with neutrophils, lymphocytes, neutrophils and lymphocytes ratio (NLR), creatinine and glucose.

Comparison of characteristics of admitted cases infected with Alpha and non-Alpha variants

Given the reported association between increased disease severity and transmission with the Alpha variant, we compared demographic, physiological and laboratory parameters between admitted cases with infection caused by Alpha variant (n=400) with non-Alpha (n=910) variants (Table 2).

For peer review only

Table 2: Descriptive statistics of the cohort for non-Alpha variant (n=910) and Alpha variant (n-400) admissions

	Missing	Non Alpha variant n (%)	Alpha variant n (%)	Non Alpha variant value [IQR]	Alpha variant value [IQR]	P-Value
Demographics						
Age	0			62.0 [49.0,78.0]	64.0 [52.0,78.0]	0.22
Male		530 (58.2)	208 (52.0)			0.042
Ethnicity	0					0.402
White		358 (39.3)	164 (41.0)			
Asian		71 (7.8)	38 (9.5)			
Black-African		191 (21.0)	67 (16.8)			
Black-Caribbean		78 (8.6)	27 (6.8)			
Mixed		16 (1.8)	6 (1.5)			
Other		50 (5.5)	23 (5.8)			
Unknown		146 (16.0)	75 (18.8)			
BMI	334			27.1 [23.8,31.7]	28.1 [24.0,34.2]	0.036
BMI>30		226 (24.8)	121 (30.2)			0.048
BMI>40		36 (4.0)	26 (6.5)			0.063
Physiological parameters						
Heart Rate	198			84.0 [74.0,94.0]	80.0 [72.0,90.0]	0.001
Heart Rate>100		118 (13.0)	36 (9.0)			0.05
Blood pressure						
Systolic	201			125.0 [113.0,139.5]	127.0 [115.0,142.0]	0.138
Diastolic	201			73.0 [65.0,80.0]	75.0 [67.0,83.0]	0.01
MAP	201			90.7 [82.3,99.2]	92.7 [84.0,101.7]	0.022
Respiratory Rate	194			19.0 [18.0,21.0]	19.0 [18.0,22.0]	0.591
Respiratory Rate>20		209 (23.0)	96 (24.0)			0.737
Hypoxia	0	392 (62.5)	217 (70.0)			0.029
Temperature	199			36.9 [36.4,37.5]	36.6 [36.2,37.1]	<0.001
NEWS2	0					0.038
0		107 (11.8)	43 (10.8)			
1		125 (13.7)	39 (9.8)			
2		127 (14.0)	53 (13.2)			
2+		391 (43.0)	207 (51.7)			
nan		160 (17.6)	58 (14.5)			
Laboratory parameters						
Neutrophils	2			4.9 [3.4,7.6]	4.8 [3.3,6.9]	0.479
Lymphocytes	1			0.9 [0.6,1.3]	0.8 [0.5,1.2]	0.005

For peer review only - http://bmjopen.bmj.com/site/about/guidelines.xhtml

NLR	2			5.4 [3.1,9.9]	5.8 [3.5,10.2]	0.195
Creatinine	16			83.0 [64.0,115.0]	92.0 [74.0,126.0]	<0.001
Urea	536			6.8 [4.3,12.0]	6.6 [4.4,10.6]	0.573
Estimated GFR	43			73.0 [48.5,97.0]	63.5 [44.0,81.0]	<0.001
Albumin	107			37.0 [33.0,41.0]	38.0 [34.0,41.0]	0.009
CRP	21			70.0 [25.0,142.0]	54.0 [24.0,102.0]	<0.001
DDimer	727			1.1 [0.6,2.8]	0.9 [0.5,1.9]	0.019
Ferritin	501			815.0 [366.2,1499.0]	712.0 [357.5,1294.0]	0.341
Co-morbidities						
Stroke	0	74 (8.1)	22 (5.5)			0.117
TIA	0	12 (1.3)	5 (1.2)			0.87
Hypertension	0	315 (34.6)	144 (36.0)			0.674
Diabetes	0	267 (29.3)	106 (26.5)			0.326
AF	0	72 (7.9)	42 (10.5)			0.154
IHD	0	162 (17.8)	78 (19.5)			0.513
Heart Failure	0	61 (6.7)	34 (8.5)			0.299
COPD	0	74 (8.1)	32 (8.0)			0.977
Asthma	0	84 (9.2)	39 (9.8)			0.846
Cancer	0	64 (7.0)	21 (5.2)			0.278
Kidney disease	0	122 (13.4)	62 (15.5)			0.359
HIV	0	22 (2.4)	10 (2.5)			0.916
Solid organ transplant	0	25 (2.7)	19 (4.8)			0.092
Frailty	0	204 (22.4)	58 (14.5)			0.001

† p-value was from Kruskal-Wallis test for continuous variables and Chi-squared test for categorical variables.

Abbreviations: BMI: Body Mass Index, TIA: Transient Ischaemic Attack, IHD: Ischemic Heart Disease, COPD: Chronic Obstructive Pulmonary Disease, HIV: Human Immunodeficiency Virus, MAP:Mean Arterial Pressure, SPO2: Oxygen Saturation, GFR: Glomerular Filtration Rate, CRP: C-Reactive Protein, NLR: Neutrophils and Lymphocytes Ratio, NEWS2: National Early Warning Score 2.

For peer review only

Groups with non-Alpha and Alpha variants were not significantly different in mean age (62yrs vs 64yrs, p=0.22) or ethnicity. The proportion of admissions who were female was larger in the group infected with the Alpha variant compared to those infected by non-Alpha variants (48.0% vs 41.8%, p=0.01).

Cases infected with the Alpha variant were less likely to be frail (14.5% vs 22.4% p=0.001). A higher proportion of those in the Alpha variant group were obese (30.2% v 24.8%, p=0.048). Other minor differences in comorbidities between groups are shown in Table 2, but did not reach statistical significance.

On admission a higher proportion of those infected with the Alpha variant were hypoxic (70.0% vs 62.5%, p=0.029), the main indicator of severe disease. CRP on admission was lower in the Alpha variant group (54 mg/L IQR: 24.0-102.0) compared to those infected with non-Alpha variants (70 mg/L, IQR: 25.0-142.0 p<0.001). Differences in other laboratory parameters did not meet either statistical or clinical significance.

Multivariate analysis of factors associated with severity of COVID-19 on admission

Multivariate logistic regression was applied to look at associations with severity of disease on admission as measured by hypoxia (Table 3), equivalent to WHO ordinal scale of ≥4 [12]. Age, sex, ethnicity, comorbidities and variant status (Alpha vs non-Alpha) were entered into the model. Severity of disease on admission, as measured by hypoxia, was the outcome variable. Age was a significant predictor of severity, with an odds ratio of 1.02 (CI 1.01-1.03, p<0.001) for hypoxia on admission for every advancing year. Obesity was associated with severity, giving an OR 1.70 (CI 1.28-2.26, p<0.001). Infection with the Alpha variant was also associated with increased hypoxia on admission (OR 1.68 CI: 1.26-2.24, p<0.001). Other variables were not significantly associated with hypoxia on admission, including sex, ethnicity and comorbidities.

For peer review only

Table 3: Odds ratios for severity (hypoxia) at admission from multivariate logistic regression model

	OR	p-value	95% CI
Age	1.02	<0.001	1.01 - 1.03
Male	0.96	0.75	0.73 - 1.25
Ethnicity			
non-White	1.15	0.35	0.86 - 1.55
Unknown	1.20	0.36	0.81 - 1.77
**Comorbidity**			
BMI>30	1.70	<0.001	1.28 - 2.26
Cardiovascular	0.79	0.15	0.58 - 1.09
Hypertension	1.11	0.52	0.81 - 1.51
Diabetes	0.75	0.07	0.55 - 1.02
Chronic respiratory disease	1.20	0.32	0.83 - 1.74
Cancer	0.60	0.06	0.35 - 1.02
Kidney disease	0.74	0.17	0.48 - 1.14
HIV	1.74	0.16	0.80 - 3.78
Organ transplant	0.79	0.55	0.37 -1.71
Frailty	0.96	0.85	0.64 - 1.45
Alpha variant	1.68	<0.001	1.26 - 2.24

Abbreviations: BMI: Body mass index, HIV: Human Immunodeficiency Virus

For peer review only

Comparison of non-sequenced and sequenced cases in wave two

We assessed for differences between the non-sequenced and sequenced inpatient cases to identify any possible bias in those that were sequenced. Demographics, admission physiological and laboratory parameters, and the outcome measure of hypoxia on admission are presented in Table 4. There was no significant difference of the proportion with the outcome measure, hypoxia on admission, in both the sequenced and non-sequenced inpatient group (47% vs 50%, p=0.381). There was no significant difference in the proportion of males in the sequenced group compared to the non-sequenced group (52.2% vs 53.8%, p=0.595), as with obesity (39.5% vs 38.4%, p=0.783) or the proportion of those from non-White ethnic backgrounds (41.4% vs 40.5%, p=0.934). On average, sequenced inpatient cases were significantly older (63 vs 57 years, p<0.001) and had a larger proportion of some comorbidities than non-sequenced cases.

For peer review only

Table 4: Patient characteristics of sequenced and non-sequenced inpatients in wave two.

	Non-sequenced	Sequenced	P-Value
n	1031	472	
Age (SD)	57.3 (21.0)	62.9 (19.9)	<0.001
Male (%)	538 (52.2)	254 (53.8)	0.595
Ethnicity (%)			0.934
White	418 (40.5)	194 (41.1)	
non-White	417 (40.4)	192 (40.7)	
Unknown	196 (19.0)	86 (18.2)	
**Comorbidities**			
BMI>30 (%)	302 (38.4)	139 (39.5)	0.783
Cardiovascular (%)	218 (21.1)	142 (30.1)	<0.001
Hypertension (%)	300 (29.1)	172 (36.4)	0.005
Diabetes (%)	269 (26.1)	127 (26.9)	0.787
Chronic respiratory Disease (%)	143 (13.9)	82 (17.4)	0.091
Cancer (%)	46 (4.5)	26 (5.5)	0.452
Kidney Disease (%)	116 (11.3)	74 (15.7)	0.021
HIV (%)	26 (2.5)	11 (2.3)	0.966
Organ transplant (%)	31 (3.0)	18 (3.8)	0.509
Frailty (%)	108 (10.5)	76 (16.1)	0.003
Hypoxia (%)	491 (47.6)	237 (50.2)	0.381

Abbreviations: BMI: Body mass index, HIV: Human Immunodeficiency Virus.

For peer review only

Discussion

Our data from a large, multi-site healthcare institution in one of the worst affected regions internationally provides a large dataset for in-depth comparison; for instance we report a similar number of cases as reported from a national observational cohort study from Japan [18]. Our hospitalised cohort shares similar demographics to other city populations in the UK, representative of London with around 40% of individuals from non-White ethnicities [19] . This compares to national population studies where the average age of cases was much lower and with lower proportion from non-White ethnciities [8,20].

There were threefold more SARS-CoV-2 RNA positive cases reported by the hospital laboratory in wave two. Partly this is attributed to increased testing capacity and changing testing strategy throughout 2020 (Supplementary Figure 1). Due to capacity limits, during wave one it was not local policy to offer testing to outpatients and those not requiring admission, instead relying on clinical diagnosis. Healthcare workers were not offered occupational health testing until the end of wave one. We therefore restricted comparison to inpatient and nosocomial cases.

There were almost twice as many admitted cases in wave two compared with wave one (1503 v 838). This is consistent with a higher local community incidence as reported by the ONS infection survey with 3.5% of individuals in London infected in January 2021 [21], compared with 2.2% of individuals in London at the peak of wave one [1]. The increase in peak hospital occupancy in wave two has also been reported nationally [22]. A major contributor to this increase in hospital admissions is likely to be the emergence of the Alpha variant, which is reported to be more transmissible [7].

Our finding is the first study in hospitalised cohorts to show increased severity of disease with the Alpha variant, as defined by hypoxia on admission which is equivalent to WHO ordinal scale of ≥4 [12] and a key marker of severe disease. Hypoxia on admission was chosen as a marker of severity

For peer review only

to prevent confounding of results by changes in management of hospitalised patients across the pandemic. For instance treatment steroids, which were introduced during the study period around November 2020, have been shown to reduce risk of ventilation and death [23]. Other changes in management, such as proning, anticoagulation and tocilizumab could also confound the outcomes of death and ICU admission. Hypoxia on admission is not at risk of confounding by changes in management of cases, as currently no significant management or treatment options are deployed in the community. The validity of using hypoxia as a marker of severity is shown by the clinical characteristics of SARS-COV-2, with respiratory illness causing hypoxia in a minority of cases and with a smaller proportion having respiratory failure necessitating ventilation [24].

Our finding of increased virulence with the Alpha variant is consistent with that reported in community studies, which show increased hospitalisation [20] and mortality [8]. Notably however, these community studies failed to control for comorbidities [Keogh]. The association with severity we find persists even after adjustment for age, sex, and comorbidities. Moreover, testing in the first wave prior to emergence of the Alpha variant was strict due to limited testing capacity, potentially leading to an ascertainment bias towards more severe cases in the first wave. This makes it even more striking that the association of the Alpha variant with severe disease is so prominent.

Notably the only other published study in hospital cohorts showed no difference in severity as measured by the composite outcome of need for ventilation or death [10]. Broadly the two cohorts from these hospital cohorts are similar, with an average age of around 60 and a high proportion of non-white ethnicities. In general this supports the external validity of our findings, however replication in dissimilar cohorts are awaited. The difference between findings in our study and those of [10] may be related to the choice of outcome. Our choice of outcome, hypoxia on admission, represents the natural history of disease prior to medical intervention as no treatments are currently deployed in the community. The mortality outcome investigated by Frampton *et al,* is after hospital treatment which may ameliorate the severity that we find with the alpha variant, thereby explaining the differences in severity seen between our studies Interestingly, despite male sex being widely

For peer review only

reported to be a risk factor for severe disease our multivariate model confirms findings by these authors that sex is not significantly associated with severity in hospitalised cohorts after adjusted analysis.

The lack of association between severity and male sex may corresponds to the increase in the proportion of females in the admitted cohort of wave two and those infected with Alpha, accounting for an extra 5% of admissions with SARS-CoV-2 infection. A study in press [25] suggests the Alpha variant may be more severe in hospitalised females, who may have increased mortality and/or requirement for ICU care. Our data, showing an increase in the proportion of females in the admission cohort and lack of expected association of severity with male sex is consistent with the finding that Alpha may show increased virulence in females.

We also included an assessment of bias by comparing characteristics of non-sequenced cases with those successfully sequenced. Whilst sequenced patients were older and more comorbid there was no significant difference between the proportion with the outcome measure of hypoxia on admission between our sequenced and non-sequenced cases. This suggests no significant bias towards severity in the sequenced group, which was predominantly made up of cases of the Alpha variant.

Admitted cases in wave two were also around half as likely to have a diagnosis of frailty, which may be due to fewer admissions from care homes during wave two, which has been reported both nationally [26] and internationally [27]. Additionally, admitted cases were around a third less likely to have cancer in wave two. Both of these reductions may also be as a result of individuals shielding, and therefore at reduced risk for acquiring SARS-CoV-2 infection. Other differences in comorbidities between waves were small and of unclear clinical significance.

One additional striking observation was the similarity in number of nosocomial cases in wave one (n=96 of 934 [10%] inpatient cases) and wave two (n=137 of 1640 [8%] inpatient cases). This incidence of nosocomial infection is a major challenge for UK healthcare institutions, with associated

For peer review only - http://bmjopen.bmj.com/site/about/guidelines.xhtml

crude mortality at around 30% during the first wave [28,29]. Interestingly, nosocomial cases in wave one increased and started to fall before impact of the main infection control interventions of banning hospital visitors (March 25th), introducing universal surgical mask wearing (28th March 2020) and universal regular inpatient screening (after the first wave). In comparison, all these measures were in place prior to the second wave. The similar number of cases in wave two may in part be due to increased inpatient screening, which would identify asymptomatic cases, or introduction of the more transmissible Alpha variant which made up the vast majority of our sequenced nosocomial cases.

Some healthcare institutions report far fewer nosocomial acquisitions; for instance an academic hospital in Boston, USA reported only 2 nosocomial cases in over 9000 admissions [30]. This could be due to greater availability of side rooms for isolation or their use of N95 masks by HCWs, which may decrease transmission between HCWs and patients. In contrast, current UK public health policy recommends surgical facemasks for patient interactions unless performing aerosol generating procedures [31]. For this reason it will be important to further investigate the factors involved in nosocomial acquisition in both waves.

Our study is limited by studying a population comes from one city and therefore needs to be compared with findings in other regions. Our dataset included cases confirmed by SARS-CoV-2 RNA testing in our laboratory, so may miss those diagnosed only clinically. A follow-up study is needed to assess the effect of the Alpha variant on mortality, when the wave two cohort has completed hospital stay. We could not compare outcomes after hospital admission, such as ICU admission or mortality, due to changes in in-hospital management between waves. In addition, we were unable to include some variables associated with severity in other studies due to few cases with these features (e.g. pregnancy) or due to poor coding in the dataset (e.g. liver disease), which prevents us from commenting on the risk associated with these variables.

The number of cases diagnosed, admissions and nosocomial cases were higher in wave two than wave one, likely due to the increased incidence caused by the more transmissible Alpha variant.

For peer review only

Infection with the Alpha variant was associated with severity as measured by hypoxia on admission, the first such finding in hospitalised cohorts. Our findings support growing evidence that emerging variants may have altered virulence as well as increased transmissibility, with such evidence providing support for public health efforts to contain their spread. More broadly, it also increases understanding of the emergence of novel pathogens as they adapt to human hosts.

For peer review only

Contributions

LBS and WW were involved in the conceptualization, methodology, formal analysis of the synthesised data and writing (original drafting, review, and editing). TC, AA-M and GN were involved in investigation being responsible for whole genome sequencing and analysis of results. RB, FH and LdJ were involved in resources, administration and data curation. YW, JE, VC were involved in supervision, funding acquisition and drafting (review and editing). All authors agreed the final manuscript.

For peer review only

References

1 Edelstein M, Obi C, Chand M, *et al.* SARS-CoV-2 infection in London, England: changes to community point prevalence around lockdown time, March–May 2020. *J Epidemiol Community Health* Published Online First: 1 October 2020. doi:10.1136/jech-2020-214730

2 Official UK Coronavirus Dashboard. https://coronavirus.data.gov.uk/details/healthcare (accessed 1 Mar 2021).

3 Kirby T. New variant of SARS-CoV-2 in UK causes surge of COVID-19. *Lancet Respir Med* 2021;**9**:e20–1. doi:10.1016/S2213-2600(21)00005-9

4 Preliminary genomic characterisation of an emergent SARS-CoV-2 lineage in the UK defined by a novel set of spike mutations. 2020.https://virological.org/t/preliminary-genomic-characterisation-of-an-emergent-sars-cov-2-lineage-in-the-uk-defined-by-a-novel-set-of-spike-mutations/563 (accessed 24 Jan 2021).

5 Sparrow A, Carroll R, Pidd H. UK Covid: Boris Johnson says 'great strides' made with vaccinations but many are yet to be reached – as it happened. The Guardian. 2021.http://www.theguardian.com/politics/live/2021/feb/10/uk-covid-news-shapps-summer-holidays-abroad-travel-coronavirus-vaccine-live-latest-updates (accessed 1 Mar 2021).

6 Chand M, Others. Investigation of novel SARS-COV-2 variant: Variant of Concern 202012/01 (PDF). *Public Health England PHE* 2020.

7 Volz E, Mishra S, Chand M, *et al.* Transmission of SARS-CoV-2 Lineage B.1.1.7 in England: Insights from linking epidemiological and genetic data. doi:10.1101/2020.12.30.20249034

8 Davies NG, Jarvis CI, John Edmunds W, *et al.* Increased mortality in community-tested cases of SARS-CoV-2 lineage B.1.1.7. *Nature* 2021;**593**:270–4. doi:10.1038/s41586-021-03426-1

9 Challen R, Brooks-Pollock E, Read JM, *et al.* Risk of mortality in patients infected with SARS-CoV-2 variant of concern 202012/1: matched cohort study. *BMJ* 2021;**372**. doi:10.1136/bmj.n579

10 Frampton D, Rampling T, Cross A, *et al.* Genomic characteristics and clinical effect of the emergent SARS-CoV-2 B.1.1.7 lineage in London, UK: a whole-genome sequencing and hospital-based cohort study. *Lancet Infect Dis* Published Online First: 12 April 2021. doi:10.1016/S1473-3099(21)00170-5

11 Surveillance definitions for COVID-19. https://www.ecdc.europa.eu/en/covid-19/surveillance/surveillance-definitions (accessed 24 Jan 2021).

12 Print RB. COVID-19 Therapeutic Trial Synopsis. 2020.https://www.who.int/publications/i/item/covid-19-therapeutic-trial-synopsis (accessed 8 Aug 2021).

13 Quick J. nCoV-2019 sequencing protocol v1 (protocols.io.bbmuik6w). protocols.io. 2020. doi:10.17504/protocols.io.bbmuik6w

14 Artic Network. https://artic.network/ncov-2019/ncov2019-bioinformatics-sop.html (accessed 24 Jan 2021).

15 *pangolin*. Github https://github.com/cov-lineages/pangolin (accessed 24 Jan 2021).

16 Rambaut A, Loman N, Pybus O, *et al.* C.-19 GC UK, Preliminary genomic characterisation of an emergent SARS-CoV-2 lineage in the UK defined by a novel set of spike mutations. virological. org (2020).

17 e-research. Acknowledging - Rosalind research computing infrastructure. https://rosalind.kcl.ac.uk/acknowledging/ (accessed 6 Mar 2021).

18 Saito S, Asai Y, Matsunaga N, *et al.* First and second COVID-19 waves in Japan: A comparison of disease severity and characteristics. Journal of Infection. 2020. doi:10.1016/j.jinf.2020.10.033

19 White E. Ethnicity and National Identity in England and Wales - Office for National Statistics. 2012.https://www.ons.gov.uk/peoplepopulationandcommunity/culturalidentity/ethnicity/articles/ethnicityandnationalidentityinenglandandwales/2012-12-11 (accessed 18 Jun 2021).

20 Nyberg T, Twohig KA, Harris RJ, *et al.* Risk of hospital admission for patients with SARS-CoV-2 variant B.1.1.7: cohort analysis. *BMJ* 2021;**373**. doi:10.1136/bmj.n1412

21 Kara Steel And. Coronavirus (COVID-19) Infection Survey, UK - Office for National Statistics. 2021.https://www.ons.gov.uk/peoplepopulationandcommunity/healthandsocialcare/conditionsanddiseases/bulletins/coronaviruscovid19infectionsurveypilot/8january2021 (accessed 1 Feb 2021).

22 England NHS. NHS England » Hospitals admit one third of COVID patients in a single month. https://www.england.nhs.uk/2021/02/hospitals-admit-one-third-of-covid-patients-in-a-single-month/ (accessed 1 Mar 2021).

23 RECOVERY Collaborative Group, Horby P, Lim WS, *et al.* Dexamethasone in Hospitalized Patients with Covid-19 - Preliminary Report. *N Engl J Med* Published Online First: 17 July 2020. doi:10.1056/NEJMoa2021436

24 Wu Z, McGoogan JM. Characteristics of and important lessons from the coronavirus disease 2019 (COVID-19) outbreak in China: summary of a report of 72 314 cases from the Chinese Center for Disease Control and Prevention. *JAMA* 2020;**323**:1239–42.https://jamanetwork.com/journals/jama/article-abstract/2762130

25 Stirrup OT, Boshier FAT, Venturini C, et al. SARS-CoV-2 lineage B.1.1.7 is associated with greater disease severity among hospitalised women but not men. medRxiv; 2021. DOI: 10.1101/2021.06.24.21259107.

26 John S. Deaths involving COVID-19 in the care sector, England and Wales - Office for National Statistics. 2020.https://www.ons.gov.uk/peoplepopulationandcommunity/birthsdeathsandmarriages/deat

For peer review only

hs/articles/deathsinvolvingcovid19inthecaresectorenglandandwales/deathsoccurringupto12june2020andregisteredupto20june2020provisional (accessed 25 Feb 2021).

27 Ioannidis JPA, Axfors C, Contopoulos-Ioannidis DG. Second versus first wave of COVID-19 deaths: shifts in age distribution and in nursing home fatalities. *medRxiv* 2020;:2020.11.28.20240366. doi:10.1101/2020.11.28.20240366

28 Carter B, Collins JT, Barlow-Pay F, *et al.* Nosocomial COVID-19 infection: examining the risk of mortality. The COPE-Nosocomial Study (COVID in Older PEople). *J Hosp Infect* 2020;**106**:376–84. doi:10.1016/j.jhin.2020.07.013

29 Snell LB, Fisher CL, Taj U, Stirrup O, Merrick B, Alcolea-Medina A, Charalampous T, Signell AW, Wilson HD, Betancor G, Kia Ik MT, Cunningham E, Cliff PR, Pickering S, Galao RP, Batra R, Neil SJD, Malim MH, Doores KJ, Douthwaite ST, Nebbia G; COVID-19 Genomics UK (COG-UK) consortium. Combined epidemiological and genomic analysis of nosocomial SARS-CoV-2 infection early in the pandemic and the role of unidentified cases in transmission. Clin Microbiol Infect. 2021 Aug 13:S1198-743X(21)00435-3. doi: 10.1016/j.cmi.2021.07.040. Epub ahead of print

30 Rhee C, Baker M, Vaidya V, *et al.* Incidence of Nosocomial COVID-19 in Patients Hospitalized at a Large US Academic Medical Center. *JAMA Netw Open* 2020;**3**:e2020498. doi:10.1001/jamanetworkopen.2020.20498

31. Public Health England. COVID-19: infection prevention and control (IPC). GOV.UK; 10 Jan 2020 [cited 15 Mar 2021]. Available: https://www.gov.uk/government/publications/wuhan-novel-coronavirus-infection-prevention-and-control

For peer review only

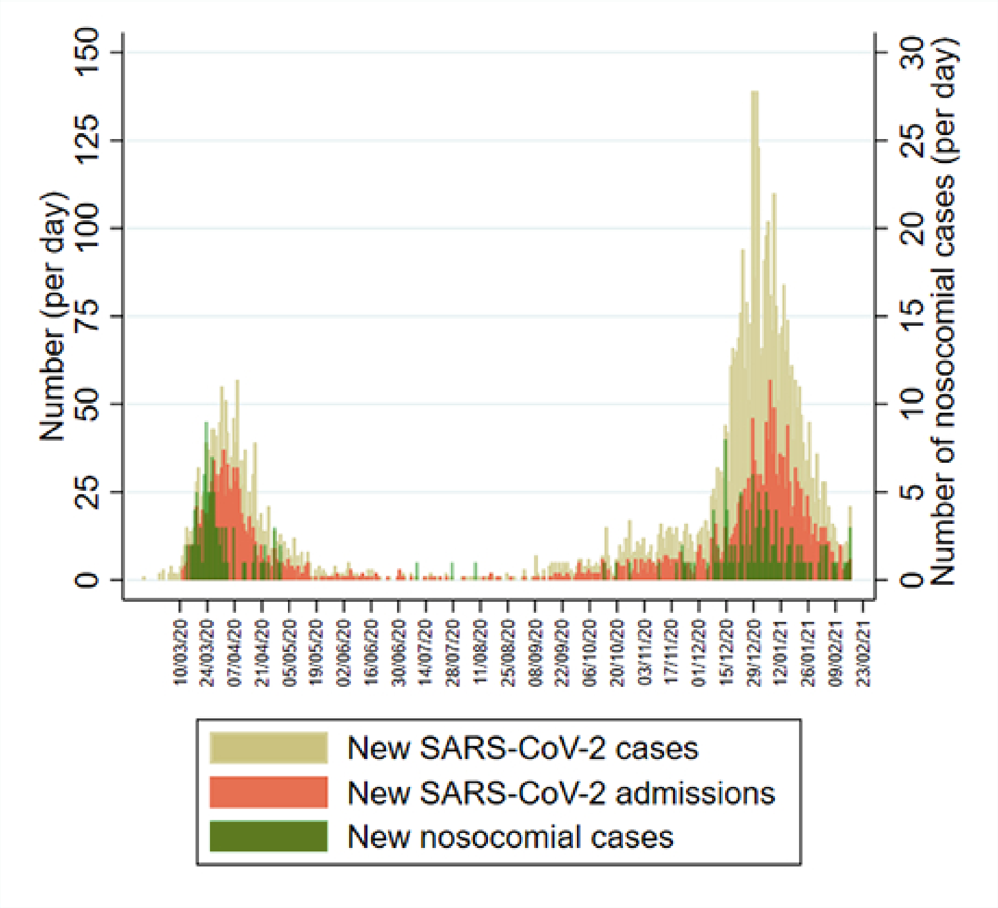

Distribution of laboratory confirmed SARS-CoV-2 cases over time. Daily incidence of new cases (beige), newly admitted cases (orange) and nosocomial acquisitions (green) over time.

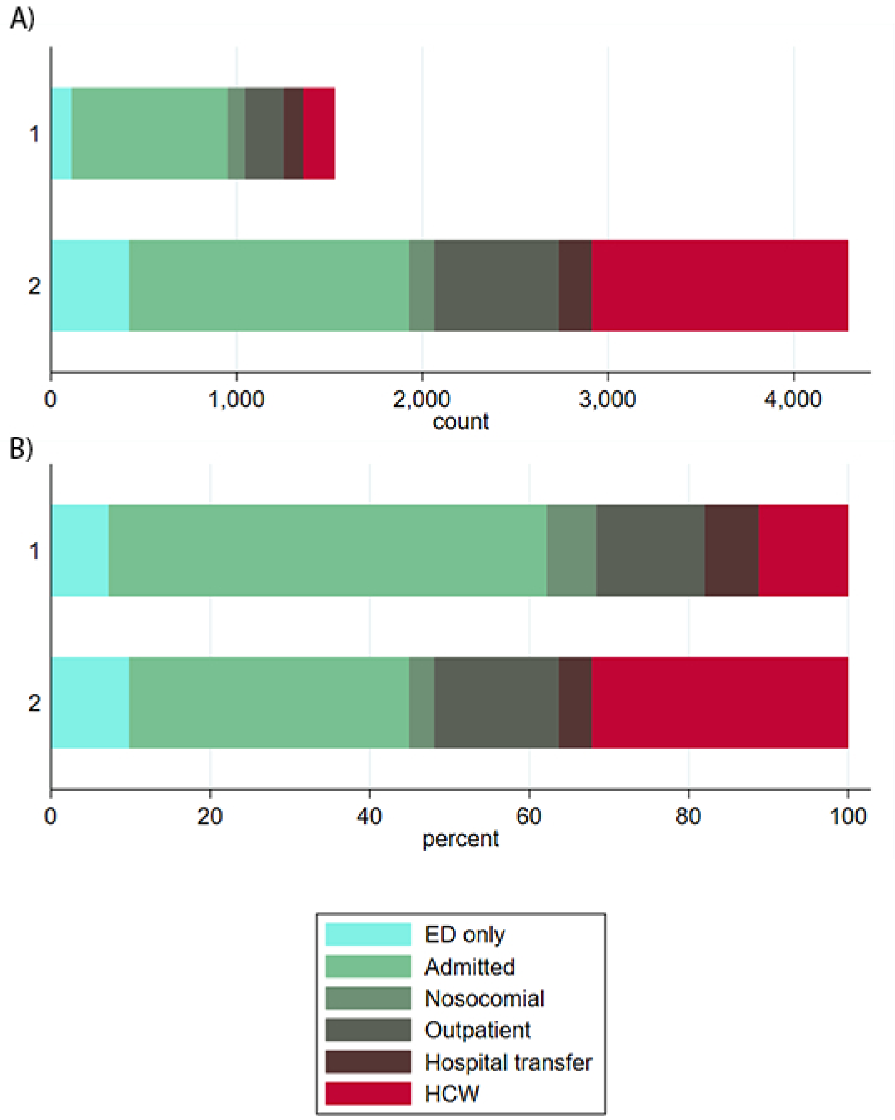

(A) Absolute number of cases within the different hospital cohorts during wave one (upper) and wave two (lower). (B) Proportion of cases within the different hospital cohorts during wave one (upper) and wave two (lower).

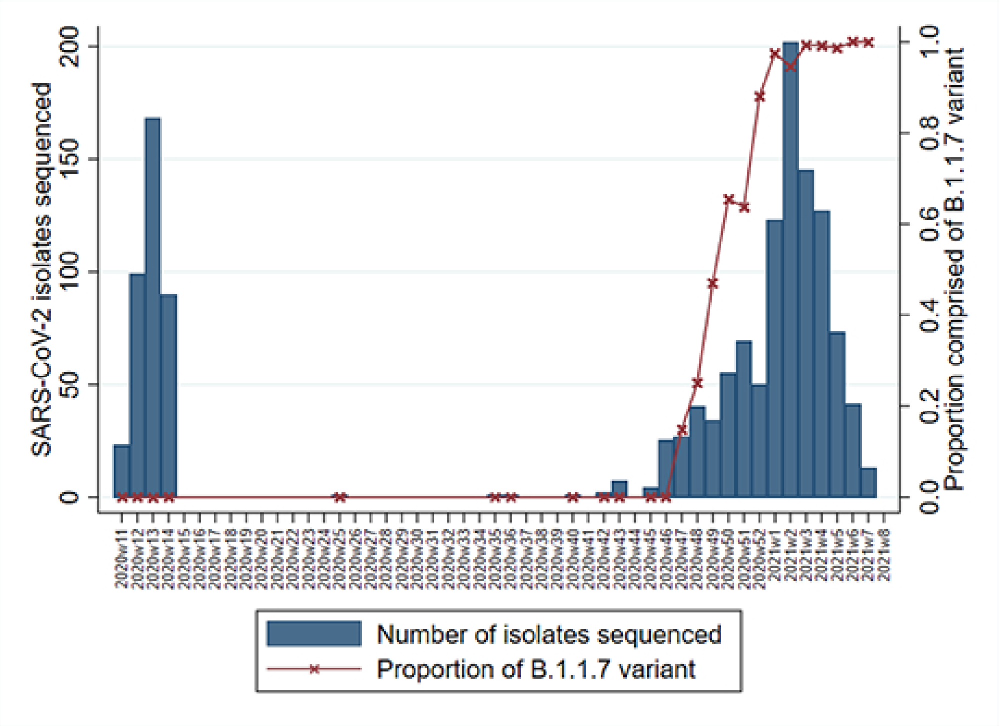

Number of cases with sequenced SARS-CoV-2 isolates by epi-week (bar) and the proportion of which were made up of the alpha variant B.1.1.7 (red line)

190x138mm (72 x 72 DPI)

Supplementary Material

Risk factors for severe disease from COVID-19 - lIterature review

Risk factor	References	Comment on inclusion in multivariable model
Age	[1–5]	Included as a continuous variable.
Sex	[2,5]	Included as binary variable.
Co-morbiidities<br>1. Cancer<br>2. Chronic kidney disease<br>3. Chronic lung disease<br>4. Dementia<br>5. DIabetes<br>6. Cardiac disease<br>7. HIV<br>8. Immunocompromise<br>9. Liver disease<br>10. Obesity<br>11. Pregnancy<br>12. Transplant<br>13. Stroke<br>14. Frailty	1. [2,3,5–7]<br>2. [2,4,6]<br>3. [2,3,5,6]<br>4. [5,6]<br>5. [2,3,6]<br>6. [2,4–6]<br>7. [6,8]<br>8. [2,6]<br>9. [2,5,6]<br>10. [4,6,9–11]<br>11. [6,12]<br>12. [2,6]<br>13. [2,6]<br>14. [13–15]	1. Included<br>2. Included<br>3. Included<br>4. Poorly coded in our dataset so not included.<br>5. Included<br>6. Included<br>7. Included<br>8. Poorly coded in our dataset so not included.<br>9. Poorly coded in our dataset so not included<br>10. Included<br>11. Small number of pregnant individuals in our dataset so not included<br>12. Included<br>13. Included with cardiovascular disease
Ethnicity	[2,5,16,17]	Evidence suggests individuals of non-White ethnicities are at increased risk in multiple territories. Included as categorical variable.
Socioeconomic background	[18,19]	These data suggest risk posed by certain ethnicity may be related to socioeconomic background. For simplicity we included only ethnicity variable.
Variant status	[20–22]	Alpha variant associated with increased mortality in population level studies, but not in single hospitalised study (full discussion in main text)

REFERENCES

1 Richardson S, Hirsch JS, Narasimhan M, *et al.* Presenting Characteristics, Comorbidities, and Outcomes Among 5700 Patients Hospitalized With COVID-19 in the New York City Area. *JAMA* 2020;**323**:2052–9.

2 Williamson EJ, Walker AJ, Bhaskaran K, *et al.* Factors associated with COVID-19-related death using OpenSAFELY. *Nature* 2020;**584**:430–6.

3 Wu Z, McGoogan JM. Characteristics of and Important Lessons From the Coronavirus Disease 2019 (COVID-19) Outbreak in China: Summary of a Report of 72 314 Cases From the Chinese Center for Disease Control and Prevention. *JAMA* 2020;**323**:1239–42.

4 Petrilli CM, Jones SA, Yang J, *et al.* Factors associated with hospital admission and critical illness among 5279 people with coronavirus disease 2019 in New York City: prospective cohort study. *BMJ* 2020;**369**:m1966.

5 Harrison SL, Fazio-Eynullayeva E, Lane DA, *et al.* Comorbidities associated with mortality in 31,461 adults with COVID-19 in the United States: A federated electronic medical record analysis. *PLoS Med* 2020;**17**:e1003321.

6 Know WYNT. People with Certain Medical Conditions. https://www.cdc.gov/coronavirus/2019-ncov/need-

For peer review only

For peer review only - http://bmjopen.bmj.com/site/about/guidelines.xhtml

extra-precautions/people-with-medical-conditions.html

7 Dai M, Liu D, Liu M, *et al.* Patients with Cancer Appear More Vulnerable to SARS-CoV-2: A Multicenter Study during the COVID-19 Outbreak. *Cancer Discov* 2020;**10**:783–91.

8 Geretti AM, Stockdale AJ, Kelly SH, *et al.* Outcomes of COVID-19 related hospitalization among people with HIV in the ISARIC WHO Clinical Characterization Protocol (UK): a prospective observational study. *Clin Infect Dis* Published Online First: 23 October 2020. doi:10.1093/cid/ciaa1605

9 Lighter J, Phillips M, Hochman S, *et al.* Obesity in Patients Younger Than 60 Years Is a Risk Factor for COVID-19 Hospital Admission. *Clin Infect Dis* 2020;**71**:896–7.

10 Tartof SY, Qian L, Hong V, *et al.* Obesity and Mortality Among Patients Diagnosed With COVID-19: Results From an Integrated Health Care Organization. *Ann Intern Med* 2020;**173**:773–81.

11 Kompaniyets L, Goodman AB, Belay B, *et al.* Body Mass Index and Risk for COVID-19-Related Hospitalization, Intensive Care Unit Admission, Invasive Mechanical Ventilation, and Death - United States, March-December 2020. *MMWR Morb Mortal Wkly Rep* 2021;**70**:355–61.

12 Allotey J, Stallings E, Bonet M, *et al.* Clinical manifestations, risk factors, and maternal and perinatal outcomes of coronavirus disease 2019 in pregnancy: living systematic review and meta-analysis. *BMJ* 2020;**370**:m3320.

13 Dumitrascu F, Branje KE, Hladkowicz ES, *et al.* Association of frailty with outcomes in individuals with COVID-19: A living review and meta-analysis. *J Am Geriatr Soc* 2021;**69**:2419–29.

14 Kastora S, Kounidas G, Perrott S, *et al.* Clinical frailty scale as a point of care prognostic indicator of mortality in COVID-19: a systematic review and meta-analysis. *EClinicalMedicine* 2021;**36**:100896.

15 Yang Y, Luo K, Jiang Y, *et al.* The Impact of Frailty on COVID-19 Outcomes: A Systematic Review and Meta-analysis of 16 Cohort Studies. *J Nutr Health Aging* 2021;**25**:702–9.

16 Price-Haywood EG, Burton J, Fort D, *et al.* Hospitalization and Mortality among Black Patients and White Patients with Covid-19. *N Engl J Med* 2020;**382**:2534–43.

17 Gold JAW, Rossen LM, Ahmad FB, *et al.* Race, Ethnicity, and Age Trends in Persons Who Died from COVID-19 - United States, May-August 2020. *MMWR Morb Mortal Wkly Rep* 2020;**69**:1517–21.

18 Kabarriti R, Brodin NP, Maron MI, *et al.* Association of Race and Ethnicity With Comorbidities and Survival Among Patients With COVID-19 at an Urban Medical Center in New York. *JAMA Netw Open* 2020;**3**:e2019795.

19 Muñoz-Price LS, Nattinger AB, Rivera F, *et al.* Racial Disparities in Incidence and Outcomes Among Patients With COVID-19. *JAMA Netw Open* 2020;**3**:e2021892.

20 Davies NG, Jarvis CI, CMMID COVID-19 Working Group, *et al.* Increased mortality in community-tested cases of SARS-CoV-2 lineage B.1.1.7. *Nature* 2021;**593**:270–4.

21 Frampton D, Rampling T, Cross A, *et al.* Genomic characteristics and clinical effect of the emergent SARS-CoV-2 B.1.1.7 lineage in London, UK: a whole-genome sequencing and hospital-based cohort study. *Lancet Infect Dis* Published Online First: 12 April 2021. doi:10.1016/S1473-3099(21)00170-5

22 Challen R, Brooks-Pollock E, Read JM, *et al.* Risk of mortality in patients infected with SARS-CoV-2 variant of concern 202012/1: matched cohort study. *BMJ* 2021;**372**:n579.

For peer review only

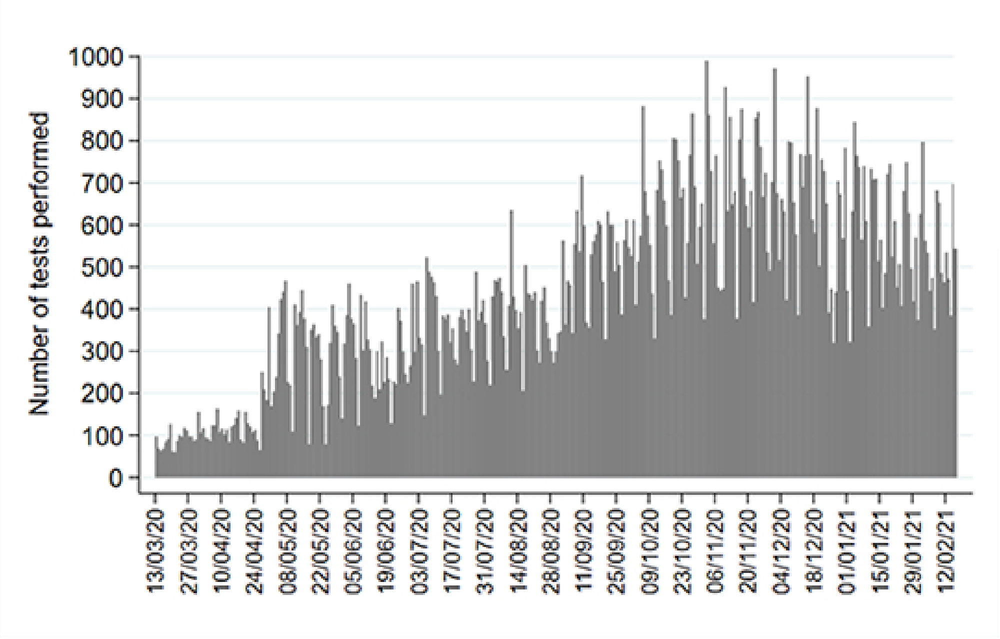

Our laboratory began testing on 13th March 2020 with initial capacity for around 150 PCR tests per day, before increasing to around 500 tests per day in late April during wave one, and up to 1000 tests per day during wave two

190x125mm (72 x 72 DPI)

STROBE 2007 (v4) Statement—Checklist of items that should be included in reports of *cohort studies*

Section/Topic	Item #	Recommendation	Reported on page #
**Title and abstract**	1	(*a*) Indicate the study's design with a commonly used term in the title or the abstract	2
		(*b*) Provide in the abstract an informative and balanced summary of what was done and what was found	2
**Introduction**			
Background/rationale	2	Explain the scientific background and rationale for the investigation being reported	6
Objectives	3	State specific objectives, including any prespecified hypotheses	6
**Methods**			
Study design	4	Present key elements of study design early in the paper	7-9
Setting	5	Describe the setting, locations, and relevant dates, including periods of recruitment, exposure, follow-up, and data collection	7-9
Participants	6	(*a*) Give the eligibility criteria, and the sources and methods of selection of participants. Describe methods of follow-up	8
		(*b*) For matched studies, give matching criteria and number of exposed and unexposed	NA
Variables	7	Clearly define all outcomes, exposures, predictors, potential confounders, and effect modifiers. Give diagnostic criteria, if applicable	7-9
Data sources/ measurement	8*	For each variable of interest, give sources of data and details of methods of assessment (measurement). Describe comparability of assessment methods if there is more than one group	8-9
Bias	9	Describe any efforts to address potential sources of bias	15
Study size	10	Explain how the study size was arrived at	12
Quantitative variables	11	Explain how quantitative variables were handled in the analyses. If applicable, describe which groupings were chosen and why	8-9
Statistical methods	12	(*a*) Describe all statistical methods, including those used to control for confounding	9-10
		(*b*) Describe any methods used to examine subgroups and interactions	NA
		(*c*) Explain how missing data were addressed	9
		(*d*) If applicable, explain how loss to follow-up was addressed	NA
		(*e*) Describe any sensitivity analyses	NA
**Results**			

For peer review only

Participants	13*	(a) Report numbers of individuals at each stage of study—eg numbers potentially eligible, examined for eligibility, confirmed eligible, included in the study, completing follow-up, and analysed	10-12
		(b) Give reasons for non-participation at each stage	15
		(c) Consider use of a flow diagram	Not done
Descriptive data	14*	(a) Give characteristics of study participants (eg demographic, clinical, social) and information on exposures and potential confounders	15-18
		(b) Indicate number of participants with missing data for each variable of interest	In tables
		(c) Summarise follow-up time (eg, average and total amount)	NA
Outcome data	15*	Report numbers of outcome events or summary measures over time	Page 18<br>Table 1, Table 2
Main results	16	(*a*) Give unadjusted estimates and, if applicable, confounder-adjusted estimates and their precision (eg, 95% confidence interval). Make clear which confounders were adjusted for and why they were included	12-18<br>Table 1,2
		(*b*) Report category boundaries when continuous variables were categorized	NA
		(*c*) If relevant, consider translating estimates of relative risk into absolute risk for a meaningful time period	NA
Other analyses	17	Report other analyses done—eg analyses of subgroups and interactions, and sensitivity analyses	None performed
**Discussion**			
Key results	18	Summarise key results with reference to study objectives	22
**Limitations**			
Interpretation	20	Give a cautious overall interpretation of results considering objectives, limitations, multiplicity of analyses, results from similar studies, and other relevant evidence	22-23
Generalisability	21	Discuss the generalisability (external validity) of the study results	23
**Other information**			
Funding	22	Give the source of funding and the role of the funders for the present study and, if applicable, for the original study on which the present article is based	4

*Give information separately for cases and controls in case-control studies and, if applicable, for exposed and unexposed groups in cohort and cross-sectional studies.

Note: An Explanation and Elaboration article discusses each checklist item and gives methodological background and published examples of transparent reporting. The STROBE checklist is best used in conjunction with this article (freely available on the Web sites of PLoS Medicine at http://www.plosmedicine.org/, Annals of Internal Medicine at http://www.annals.org/, and Epidemiology at http://www.epidem.com/). Information on the STROBE Initiative is available at www.strobe-statement.org.

For peer review only

BMJ Open

A descriptive comparison of admission characteristics between pandemic waves and multivariable analysis of the association of the Alpha variant (B.1.1.7 lineage) of SARS-CoV-2 with disease severity in inner London.

Journal:	*BMJ Open*
Manuscript ID	bmjopen-2021-055474.R2
Article Type:	Original research
Date Submitted by the Author:	07-Oct-2021
Complete List of Authors:	Snell, Luke; King's College London; Guy's and St Thomas' NHS Foundation Trust<br>Wang , Wenjuan; King's College London<br>Medina, Adela; Viapath; King's College London<br>Charalampous, Themoula; King's College London<br>Batra, Rahul; King's College London<br>de Jongh, Leonardo; Guy's and St Thomas' NHS Foundation Trust<br>Higgins, Finola; Guy's and St Thomas' NHS Foundation Trust<br>Investigators, COG-UK HOCI ; Not applicable<br>Nebbia, Gaia; King's College London; Guy's and St Thomas' NHS Foundation Trust<br>Wang, Yanzhong; King's College London<br>Edgeworth, Jonathan; King's College London; Guy's and St Thomas' NHS Foundation Trust<br>Curcin, Vasa; King's College London
**Primary Subject Heading**:	Infectious diseases
Secondary Subject Heading:	Epidemiology
Keywords:	VIROLOGY, EPIDEMIOLOGY, Epidemiology < INFECTIOUS DISEASES, Public health < INFECTIOUS DISEASES

SCHOLARONE™
Manuscripts

I, the Submitting Author has the right to grant and does grant on behalf of all authors of the Work (as defined in the below author licence), an exclusive licence and/or a non-exclusive licence for contributions from authors who are: i) UK Crown employees; ii) where BMJ has agreed a CC-BY licence shall apply, and/or iii) in accordance with the terms applicable for US Federal Government officers or employees acting as part of their official duties; on a worldwide, perpetual, irrevocable, royalty-free basis to BMJ Publishing Group Ltd ("BMJ") its licensees and where the relevant Journal is co-owned by BMJ to the co-owners of the Journal, to publish the Work in this journal and any other BMJ products and to exploit all rights, as set out in our licence.

The Submitting Author accepts and understands that any supply made under these terms is made by BMJ to the Submitting Author unless you are acting as an employee on behalf of your employer or a postgraduate student of an affiliated institution which is paying any applicable article publishing charge ("APC") for Open Access articles. Where the Submitting Author wishes to make the Work available on an Open Access basis (and intends to pay the relevant APC), the terms of reuse of such Open Access shall be governed by a Creative Commons licence – details of these licences and which Creative Commons licence will apply to this Work are set out in our licence referred to above.

Other than as permitted in any relevant BMJ Author's Self Archiving Policies, I confirm this Work has not been accepted for publication elsewhere, is not being considered for publication elsewhere and does not duplicate material already published. I confirm all authors consent to publication of this Work and authorise the granting of this licence.

For peer review only

A descriptive comparison of admission characteristics between pandemic waves and multivariable analysis of the association of the Alpha variant (B.1.1.7 lineage) of SARS-CoV-2 with disease severity in inner London.

Luke B.Snell MBBS*, Wenjuan Wang PhD*, Adela Alcolea-Medina MPhil, Themoula Charalampous PhD, , Rahul Batra MD, Leonardo de Jongh, Finola Higgins, COG-UK HOCI Investigators†, Gaia Nebbia PhD, Yanzhong Wang PhD, Jonathan D. Edgeworth PhD♯, Vasa Curcin PhD♯

1 King's College London, London, UK (L.B. Snell, W. Wang, A. Alcolea-Medina, T. Charalampous, G. Nebbia, R. Batra, Y. Wang, J.D. Edgeworth, V Curin)
2 Guy's and St Thomas' NHS Foundation Trust, London, UK (L.B. Snell, G. Nebbia, R. Batra, L. de Jongh, F. Higgins, J.D. Edgeworth)
3 Viapath, St Thomas' Hospital, London, UK (A. Alcolea-Medina)

* These first authors contributed equally
† Full list of investigator names and affiliations are given in the appendix.
♯ These authors were co–principal investigators

Corresponding author: Dr. Luke B Snell, St. Thomas' Hospital, Westminster Bridge Road, UK SE1 7EH, luke.snell@nhs.net, 02071887188

Running title: Comparison of SARS-CoV-2 waves and alpha variant severity

Article word count: 3714

For peer review only

Abstract

Background. The Alpha variant (B.1.1.7 lineage) of SARS-CoV-2 emerged and became the dominant circulating variant in the UK in late 2020. Current literature is unclear on whether the Alpha variant is associated with increased severity. We linked clinical data with viral genome sequence data to compare admitted cases between SARS-CoV-2 waves in London, and to investigate the association between Alpha variant and the severity of disease.

Methods. Clinical, demographic, laboratory and viral sequence data from electronic health record (EHR) systems was collected for all cases with a positive SARS-CoV-2 RNA test between March 13th 2020 and February 17th 2021 in a multi-site London healthcare institution. Multivariate analysis using logistic regression assessed risk factors for severity as defined by hypoxia at admission.

Results There were 5810 SARS-CoV-2 RNA positive cases of which 2341 were admitted (838 in wave one and 1503 in wave two). Both waves had a temporally aligned rise in nosocomial cases (96 in wave one, 137 in wave two). The Alpha variant was first identified on 15th November 2020 and increased rapidly to comprise 400/472 (85%) of sequenced isolates from admitted cases in wave two. A multivariate analysis identified risk factors for severity on admission, such as age (OR 1.02 [CI 1.01-1.03] for every year older, p<0.001), obesity (OR 1.70 [CI 1.28-2.26], p<0.001) and infection with the Alpha variant (OR 1.68 [CI 1.26-2.24], p<0.001).

Conclusions Our analysis is the first in hospitalised cohorts to show increased severity of disease associated with the Alpha variant. The number of nosocomial cases was similar in both waves despite the introduction of many infection control interventions before wave two.

Abstract word count: 250

For peer review only

Strengths and limitations of this study

- Published evidence on whether the Alpha variant of SARS-CoV-2 causes more severe disease (COVD-19) is mixed.
- Our study benefits from a long study window, including patients since the beginning of the SARS-CoV-2 pandemic.
- Our outcome measure for severity, hypoxia on admission, reflects the natural history of disease prior to medical intervention and hospital treatment.
- Our analysis adjusts for comorbidities, a feature missing from many of the population-level studies currently published.

For peer review only

Ethics

Ethical approval for data informatics was granted by The London Bromley Research Ethics Committee (reference (20/HRA/1871)) to the King's Health Partners Data Analytics and Modelling COVID-19 Group to collect clinically relevant data points from patients' electronic health records.

Whole genome sequencing of residual viral isolates was conducted under the COVID-19 Genomics UK (COG-UK) consortium study protocol, which was approved by the Public Health England Research Ethics and Governance Group (reference: R&D NR0195).

Acknowledgements

The authors acknowledge use of the research computing facility at King's College London, Rosalind (https://rosalind.kcl.ac.uk), which is delivered in partnership with the National Institute for Health Research (NIHR) Biomedical Research Centres at South London & Maudsley and Guy's & St. Thomas' NHS Foundation Trusts, and part-funded by capital equipment grants from the Maudsley Charity (award 980) and Guy's & St. Thomas' Charity (TR130505). The views expressed are those of the author(s) and not necessarily those of the NHS, the NIHR, King's College London, or the Department of Health and Social Care.

Funding

FH, LBS, YW, and VC are supported by the National Institute for Health Research (NIHR) Biomedical Research Centre programme of Infection and Immunity (RJ112/N027) based at Guy's and St Thomas' National Health Service NHS) Foundation Trust and King's College London.

This work was also supported by The Health Foundation and the Guy's and St Thomas' Charity.

COG-UK is supported by funding from the Medical Research Council (MRC) part of UK Research & Innovation (UKRI), the National Institute of Health Research (NIHR) and Genome Research Limited, operating as the Wellcome Sanger Institute.

For peer review only

Data sharing

Genomic data is publicly available on GISAID, please contact the authors for accession numbers.

Our ethical approval precludes public sharing of patient-level data.

Patient and public involvement

As this study was conducted to meet an urgent public health need, patient and public involvement was not sought

Competing interests

None to declare.

For peer review only

Background

SARS-CoV-2 infection has led to the death of over 4 million individuals worldwide since its emergence in China during December 2019, with over 120,000 deaths reported in the UK as of July 2021. In London, the estimated incidence of new cases in the first wave peaked around March 23rd 2020 at 2.2% [1] and then rapidly declined following non-pharmacological interventions. Hospital admissions peaked about 1 week later [2] reflecting the median period of symptoms before hospital presentation. A "second wave" of infections started in London around the beginning of October 2020 [3].

Genome sequencing identified the Alpha variant (the B.1.1.7 lineage) around the South East of England, which spread rapidly as part of the emerging second wave [4]. This occurred prior to widespread vaccination, with only 25% of the adult population receiving the first dose by mid-February 2021 [5]. The Alpha variant has been associated with increased transmissibility in community studies [6] [7], and community studies associate the variant with increased mortality [8] [9]. However, published studies in hospitalised patients suggested no increase in need for ventilation or mortality [10].

Changes in transmissibility and severity have the potential to affect the burden on healthcare systems, and modify the characteristics of cases presenting to hospitals including the demographics, comorbidities and severity of disease associated with SARS-CoV-2 infection.

Objectives

We linked clinical datasets with local SARS-CoV-2 variant analysis to compare admission characteristics of hospitalised cases during the two waves of infection and to look at the association of the Alpha variant with severity of disease at presentation to hospital.

For peer review only

Methods

Setting

Guy's and St Thomas' NHS Foundation Trust (GSTT) is a multi-site healthcare institution providing general and emergency services predominantly to the South London boroughs of Lambeth and Southwark. An acute-admitting site (St Thomas' Hospital) has an adult emergency department, with a large critical care service including one of the UK's eight nationally commissioned extra corporeal membrane oxygenation (ECMO) centres for severe respiratory failure. A second site (Guy's Hospital) provides more inpatient services such as elective surgery, cancer care and other specialist services. A paediatric hospital (Evelina London) acts as a general and specialised referral centre. Several satellite sites for specialist services like dialysis, rehabilitation and long term care are also part of the institution. GSTT receives patients from regional hospitals predominantly to critical care through ‘mutual aid' schemes.

SARS-CoV-2 laboratory testing

Our laboratory began testing on 13th March 2020 with initial capacity for around 150 PCR tests per day, before increasing to around 500 tests per day in late April during wave one, and up to 1000 tests per day during wave two (Supplementary Figure 1).

Testing commenced during the first wave on 13th March 2020 limited to cases requiring admission or inpatients who had symptoms of fever or cough, as per national recommendation; guidance suggested cases which did not require admission should not be tested. For wave two, all cases admitted to hospital were screened and underwent universal interval screening at varying time points. Staff testing for symptomatic healthcare workers was also introduced towards the end of wave one. Comparative analysis was therefore restricted to SARS-CoV-2 RNA positive cases requiring admission. Cases without laboratory confirmation of SARS-CoV-2 infection were not included.

For peer review only - http://bmjopen.bmj.com/site/about/guidelines.xhtml

Assays used for the detection of SARS-CoV-2 RNA include PCR testing using Aus Diagnostics or by the Hologic Aptima SARS-CoV-2 Assay. Nucleic acid was first extracted using the QIAGEN QIAsymphony SP system and a QIAsymphony DSP Virus/Pathogen Mini Kit (catalogue No: 937036) with the off-board lysis protocol.

Definitions and participants

Cases were identified by the first positive SARS-CoV-2 RNA test. Cases were placed in mutually exclusive categories with the following definitions: 1) outpatients 2) testing through occupational health 3) emergency department attenders not subsequently admitted within 14 days 4) patients admitted within 14 days of a positive test 5) nosocomial cases, defined based on ECDC definitions, as those having a first positive test on day 8 or later after admission to hospital where COVID-19 was not suspected on admission [11] and 6) interhospital transfers.

For the purpose of comparison, only the inpatient group admitted within 14 days following a positive test, were taken forward for onward comparison. This methodology of only including admissions was adopted to prevent increased testing during the pandemic affecting case ascertainment and biasing severity of cases. This is evidenced in Supplementary Figure 1, with tests increasing steadily from 100 per day to more than 1000 per day. Additionally, in wave two more interhospital transfers of severe cases requiring extracorporeal membrane oxygenation were received, mostly several days after admission. This category of patients were therefore excluded from analysis to prevent biasing towards severe disease.

A composite datapoint for ‘hypoxia' was created, equivalent to WHO ordinal scale of ≥4 [12], with cases taken to be hypoxic if on admission they had oxygen saturations of <94%, if they were recorded as requiring supplemental oxygen, or if the fraction of inspired oxygen was recorded as being greater than 0.21.

Determination of SARS-CoV-2 lineage

For peer review only

Whole genome sequencing of residual samples from SARS-CoV-2 cases was performed using GridION (Oxford Nanopore Technology), using version 3 of the ARTIC protocol [13] and bioinformatics pipeline [14]. Samples were selected for sequencing if the corrected CT value was 33 or below, or the Hologic Aptima assay was above 1000 RLU. During the first wave, sequencing occurred between March 1st - 31st, whilst sequencing in the second wave restarted in November 2020 - March 2021. Variants were called using updated versions of pangolin 2.0 [15]. We considered all cases in wave one to be non-Alpha variants, as our wave one cut-off of 25th July 2020 was 6 weeks prior to first identified cases of the Alpha variant in the UK [16] and before Alpha variant was first identified in our population in November 2020.

Data sources, extraction and integration

Clinical, laboratory and demographic data for all cases with a laboratory reported SARS-CoV-2 PCR RNA test on nose and throat swabs or lower respiratory tract specimens were extracted from hospital electronic health record (EHR) data sources using records closest to the test date. Data were linked to the Index of Multiple Deprivation (IMD). Age, sex and ethnicity were extracted from EPR. Self-reported ONS ethnic categories were stratified into White (British, Irish, Gypsy and White-Other) or non-White (Black [African, Caribbean, and Black-Other], Asian [Bangladeshi, Chinese, Indian, Pakistan, and Asian-Other] and Mixed/Other). Numbers for which data were missing is listed by each variable. Comorbidities and medication history were extracted from the EPR and e-Noting using natural language processing (NLP). If a comorbidity was not recorded it was assumed not to be present. Cases were characterised as having/not having a past medical history of hypertension, cardiovascular disease (stroke, transient ischaemic attack, atrial fibrillation, congestive heart failure, ischaemic heart disease, peripheral artery disease or atherosclerotic disease), diabetes mellitus, chronic kidney disease, chronic respiratory disease (chronic obstructive pulmonary disease, asthma, bronchiectasis or pulmonary fibrosis) and neoplastic disease (solid tumours, haematological neoplasias or metastatic disease). Obesity was defined as either obesity present in the notes, or recorded Body Mass Index (BMI) >= 30 kg/m^2. Medicines data were extracted using both structured queries and natural language processing tools with medical and drug dictionaries .Additionally,

For peer review only - http://bmjopen.bmj.com/site/about/guidelines.xhtml

checks on free text data were performed by a cardiovascular clinician to ensure the information was accurate.

Analysis was carried out on the secure Rosalind high-performance computer infrastructure [17] running Jupyter Notebook 6.0.3, R 3.6.3 and Python 3.7.6.

Statistical analysis and outcome measures

Descriptive statistics were summarised with mean and standard deviation (SD) for continuous variables if the distribution is normal and median and interquartile range (IQR) if the distribution is non-normal. Count and percentages were used for categorical variables. For the comparisons of variables for wave one versus wave two variables, Alpha variant versus non-Alpha variants, as well as sequenced patients versus non-sequenced patients in wave two, Kruskal-Walllis test was used for continuous variables and Chi-squared test for categorical variables with significance level of p=0.05. Multivariate analysis was performed using logistic regression to assess the odds ratios of different risk factors (including age, sex, ethnicity (White, non-White, Unknown), variant status (Alpha or non-Alpha), and cardiovascular disease, hypertension, diabetes, chronic respiratory disease, cancer, kidney disease, HIV, transplant, and frailty) for hypoxia on admission as the binary outcome indicating severity at admission. Variables to be included in the multivariate analysis were chosen by literature review and expert opinion (see Supplementary Material). Cases with missing datapoints were dropped from analysis.

For peer review only

Results

General epidemiology and results of viral sequencing.

Figure 1 shows the incidence of SARS-CoV-2 cases, SARS-CoV-2 admissions, and nosocomial cases since March 13th 2020. In total 5810 individuals had a positive SARS-CoV-2 PCR test up until the data extraction date of 17th February 2021. Two “waves” are evident with July 25th taken as a separation date between waves, at which point a minimum of 12 wave one cases remained in hospital. Wave one comprised 1528 cases (26.3%) from when laboratory testing commenced on March 13th to peak rapidly between the 1st and 8th April 2020 with 57 new cases per day, before falling to a baseline by May 12th 2020. Ninety-one percent (1391/1528) of all cases in wave one occurred during these 60 days. Wave two comprised 4282 cases (73.7%), with incidence first increasing gradually from the beginning of October. There was then a period of rapidly escalating incidence from about 10th December, peaking on 28th December 2020 when 139 cases per day were diagnosed. 3446/4282 (80%) of wave two cases were detected during a comparable 60 day period between 10th December 2020 and 8th February 2021. In both waves, nosocomial cases peaked early, increasing along with admissions but then fell while the number of community admissions continued at peak levels.

Figure 1: Distribution of laboratory confirmed SARS-CoV-2 cases over time. Daily incidence of new cases (beige), newly admitted cases (orange) and nosocomial acquisitions (green) over time.

Individuals with a positive test were placed into six categories (Figure 2). The 5810 SARS-CoV-2 cases were categorised as follows, inpatients admitted within 14 days of a positive test (n=2341), healthcare workers (n=1549), outpatients (n=874), emergency department (ED) attenders not subsequently admitted (n=532), inter-hospital transfers (n=281) and nosocomial cases (n=233). Some observed differences between waves one and two reflected the increased availability of testing particularly for outpatients (208;13.6% v 666;15.6%), people sent home from ED (111;7.3% v 421; 9.8%) and healthcare workers (171;11.2% v 1378;32.2%). There were also more interhospital transfers of known COVID-19 cases in wave two (177;4.1% v 104;6.8% in wave one). In wave two,

For peer review only

the number of admissions increased (1503; 35.1% v 838; 54.8%) along with nosocomial cases (137;3.2% v 96;6.3%) compared with wave one.

Figure 2 (A) Absolute number of cases within the different hospital cohorts during wave one (upper) and wave two (lower). **(B)** Proportion of cases within the different hospital cohorts during wave one (upper) and wave two (lower).

Figure 3 shows the 1470 successfully sequenced SARS-CoV-2 isolates over time, with 382 from wave one and 1088 from wave two. Sequencing was successful for 216/838 (26%) admitted cases from wave one, 472/1503 (31%) admitted cases in wave two, and 121/233 (52%) nosocomial cases. The proportion of Alpha variant increased rapidly after the first Alpha isolate was identified on 15th November 2020, accounting for approximately two thirds within 3 weeks, and almost 100% (600/617 isolates, 97%) in January 2021. In the second wave, the Alpha variant made up 83% (908/1088) of all sequenced isolates, 85% (400/472) of sequenced isolates from admitted cases, and 88% (51/59) of sequenced isolates from nosocomial cases. In addition, two cases of the B.1.351 beta variant of concern were also detected in the wave two admission cohort.

Figure 3 Number of cases with sequenced SARS-CoV-2 isolates by epi-week (bar) and the proportion of which were made up of the alpha variant B.1.1.7 (red line)

Comparison of characteristics of admitted cases between waves one and two

Descriptive statistics of cases admitted during wave one (n=838) and wave two (n=1503) were compared (Table 1). There was a statistically significant difference in median age of 2 years (62yrs in wave one v 60yrs in wave two, p=0.019) and admitted cases were more likely to be female in wave two (47.3% v 41.8%, p=0.011). A larger proportion of admitted cases in wave two were obese (29.1% v 24.6%, p=0.02). Comparison of comorbidities showed that those in wave two were less likely to have a diagnosis of frailty (11.5% v 22.8%, p<0.001), history of stroke (4.3% v 8.6%, p<0.001) or cancer (4.8% v 7.2%, p=0.022). There was no significant difference in proportion with

For peer review only

known comorbidities of diabetes, kidney disease, hypertension, cardiovascular disease or respiratory disease.

Table 1: Descriptive statistics of the cohort for wave one (n=838) and wave two (n=1503) admissions

	Missing	Wave one n (%)	Wave two n (%)	Wave one Median [IQR]	Wave two Median [IQR]	P-Value
Demographics						
Age	0			62.0 [49.0,78.0]	60.0 [47.0,74.0]	0.019
Male	0	488 (58.2)	792 (52.7)			0.011
Ethnicity	0					0.013
White		331 (39.5)	598 (39.8)			
Asian		64 (7.6)	121 (8.1)			
Black-African		177 (21.1)	262 (17.4)			
Black-Caribbean		73 (8.7)	98 (6.5)			
Mixed		15 (1.8)	18 (1.2)			
Other		45 (5.4)	107 (7.1)			
Unknown		133 (15.9)	299 (19.9)			
BMI	577			27.0 [23.8,31.7]	27.7 [24.0,32.9]	0.022
BMI>30		206 (24.6)	438 (29.1)			0.02
BMI>40		34 (4.1)	86 (5.7)			0.098
Physiological parameters						
Heart Rate (bpm)	360			84.0 [75.0,94.0]	81.0 [72.0,91.0]	<0.001
Heart Rate>100		105 (12.5)	142 (9.4)			0.02
Blood pressure (mmHg)						
Systolic	369			125.0 [113.0,139.0]	127.0 [115.0,141.0]	0.013
Diastolic	369			73.0 [65.0,80.0]	75.0 [68.0,82.0]	<0.001
MAP	369			90.7 [82.2,99.0]	92.3 [84.7,101.3]	<0.001
Respiratory Rate (per min)	359			19.0 [18.0,22.0]	19.0 [18.0,22.0]	0.764
Respiratory Rate>20		200 (23.9)	365 (24.3)			0.86
Hypoxia	658	370 (64.3)	726 (65.5)			0.67
Temperature (ºC)	361			36.9 [36.4,37.5]	36.6 [36.2,37.2]	<0.001
NEWS2	405					0.86
0		95 (11.3)	173 (11.5)			
1		108 (12.9)	192 (12.8)			
2		117 (14.0)	188 (12.5)			
>2		371 (44.3)	692 (46.0)			
Laboratory parameters						

For peer review only - http://bmjopen.bmj.com/site/about/guidelines.xhtml

Neutrophils (x 10^9/L)	8			4.9 [3.4,7.6]	5.0 [3.3,7.5]	0.724
Lymphocytes (x 10^9/L)	7			0.9 [0.6,1.3]	0.9 [0.6,1.4]	0.741
NLR	8			5.4 [3.1,9.9]	5.4 [3.2,9.8]	0.951
Creatinine (umol/L)	43			83.0 [64.0,115.0]	86.0 [68.0,117.0]	0.065
Urea (mmol/L)	855			7.0 [4.6,12.2]	6.0 [4.3,9.9]	0.001
Estimated GFR (ml/min)	114			73.0 [48.0,98.0]	69.0 [48.0,89.0]	0.001
Albumin (g/L)	185			37.0 [32.0,40.0]	38.0 [34.0,41.0]	<0.001
CRP (mg/L)	61			74.5 [26.0,148.0]	51.0 [18.0,103.8]	<0.001
D-Dimer (mg/L FEU)	1297			1.1 [0.6,3.0]	0.9 [0.5,2.2]	0.001
Ferritin (ug/L))	905			855.0 [394.0,1533.5]	699.0 [342.0,1359.0]	0.05
Co-morbidities						
Stroke	0	72 (8.6)	64 (4.3)			<0.001
TIA	0	9 (1.1)	20 (1.3)			0.731
Hypertension	0	288 (34.4)	464 (30.9)			0.091
Diabetes	0	246 (29.4)	384 (25.5)			0.052
AF	0	63 (7.5)	115 (7.7)			0.972
IHD	0	146 (17.4)	244 (16.2)			0.495
Heart Failure	0	54 (6.4)	105 (7.0)			0.679
COPD	0	64 (7.6)	109 (7.3)			0.796
Asthma	0	74 (8.8)	138 (9.2)			0.835
Cancer	0	60 (7.2)	72 (4.8)			0.022
Kidney disease	0	112 (13.4)	181 (12.0)			0.389
HIV	0	21 (2.5)	36 (2.4)			0.979
Solid organ Transplant	0	24 (2.9)	49 (3.3)			0.686
Frailty	0	191 (22.8)	173 (11.5)			<0.001

†: p-value was from Kruskal-Wallis test for continuous variables and Chi-squared test for categorical variables.

Abbreviations: BMI: Body Mass Index, TIA: Transient Ischaemic Attack, IHD: Ischemic Heart Disease, COPD: Chronic Obstructive Pulmonary Disease, HIV: Human Immunodeficiency Virus, MAP:Mean Arterial Pressure, SPO2: Oxygen Saturation, GFR: Glomerular Filtration Rate, CRP: C-Reactive Protein, NLR: Neutrophils and Lymphocytes Ratio, NEWS2: National Early Warning Score 2.

For peer review only

There were no significant differences between waves in the proportion with severe SARS-CoV-2 disease upon admission as judged by hypoxia (64.3% in wave one vs 65.5% in wave two, p=0.67) or tachypnoea (respiratory rate>20) (23.9% vs 24.3%, p=0.86). There were small differences in other physiological parameters on admission, some of which reached statistical significance but differences were not clinically relevant.

Laboratory markers were compared between waves (Table 1). There were small but significant differences, such as lower CRP (median 51.0 mg/dL (IQR: 18.0-103.8) v 74.5 mg/dL (IQR: 26.0-148.0), p<0.001) and lower ferritin (699.0 [IQR: 342.0-1359.0] v 855.0 [IQR: 394.0-1533.5], p=0.05) in wave two. There were other small statistically significant differences without clear clinical significance, such as a lower D-Dimer in wave two (0.9 mg/L FEU [IQR: 0.5-2.2] v 1.1 mg/L FEU [IQR:0.6-3.0], p=0.001), and lower estimated GFR (69.0 ml/min [IQR: 48.0-89.0] v 73.0 ml/min [IQR: 48.0-98.0], p=0.001), lower urea (6.0 mmol/L [IQR: 4.3-9.3] v 7.0 mmol/L [IQR: 4.6-12.2], p=0.001) and higher albumin (38.0 g/L [IQR: 34.0-41.0 g/L] vs 37.0 g/L [IQR: 32.0-40.0], p<0.001). There was no significant difference with neutrophils, lymphocytes, neutrophils and lymphocytes ratio (NLR), creatinine and glucose.

Comparison of characteristics of admitted cases infected with Alpha and non-Alpha variants

Given the reported association between increased disease severity and transmission with the Alpha variant, we compared demographic, physiological and laboratory parameters between admitted cases with infection caused by Alpha variant (n=400) with non-Alpha (n=910) variants (Table 2).

For peer review only

Table 2: Descriptive statistics of the cohort for non-Alpha variant (n=910) and Alpha variant (n-400) admissions

	Missing	Non Alpha variant n (%)	Alpha variant n (%)	Non Alpha variant value [IQR]	Alpha variant value [IQR]	P-Value
Demographics						
Age	0			62.0 [49.0,78.0]	64.0 [52.0,78.0]	0.22
Male		530 (58.2)	208 (52.0)			0.042
Ethnicity	0					0.402
White		358 (39.3)	164 (41.0)			
Asian		71 (7.8)	38 (9.5)			
Black-African		191 (21.0)	67 (16.8)			
Black-Caribbean		78 (8.6)	27 (6.8)			
Mixed		16 (1.8)	6 (1.5)			
Other		50 (5.5)	23 (5.8)			
Unknown		146 (16.0)	75 (18.8)			
BMI	334			27.1 [23.8,31.7]	28.1 [24.0,34.2]	0.036
BMI>30		226 (24.8)	121 (30.2)			0.048
BMI>40		36 (4.0)	26 (6.5)			0.063
Physiological parameters						
Heart Rate (bpm)	198			84.0 [74.0,94.0]	80.0 [72.0,90.0]	0.001
Heart Rate>100		118 (13.0)	36 (9.0)			0.05
Blood pressure (mmHg)						
Systolic	201			125.0 [113.0,139.5]	127.0 [115.0,142.0]	0.138
Diastolic	201			73.0 [65.0,80.0]	75.0 [67.0,83.0]	0.01
MAP	201			90.7 [82.3,99.2]	92.7 [84.0,101.7]	0.022
Respiratory Rate (per min)	194			19.0 [18.0,21.0]	19.0 [18.0,22.0]	0.591
Respiratory Rate>20		209 (23.0)	96 (24.0)			0.737
Hypoxia	0	392 (62.5)	217 (70.0)			0.029
Temperature (°C)	199			36.9 [36.4,37.5]	36.6 [36.2,37.1]	<0.001
NEWS2	218					0.038
0		107 (11.8)	43 (10.8)			
1		125 (13.7)	39 (9.8)			
2		127 (14.0)	53 (13.2)			
>2		391 (43.0)	207 (51.7)			
Laboratory parameters						
Neutrophils ($x10^9/L$)	2			4.9 [3.4,7.6]	4.8 [3.3,6.9]	0.479
Lymphocytes ($x10^9/L$)	1			0.9 [0.6,1.3]	0.8 [0.5,1.2]	0.005
NLR	2			5.4 [3.1,9.9]	5.8 [3.5,10.2]	0.195

For peer review only - http://bmjopen.bmj.com/site/about/guidelines.xhtml

Creatinine (umol/L)	16			83.0 [64.0,115.0]	92.0 [74.0,126.0]	<0.001
Urea (mmol/L)	536			6.8 [4.3,12.0]	6.6 [4.4,10.6]	0.573
Estimated GFR (ml/min)	43			73.0 [48.5,97.0]	63.5 [44.0,81.0]	<0.001
Albumin (g/L)	107			37.0 [33.0,41.0]	38.0 [34.0,41.0]	0.009
CRP (mg/L)	21			70.0 [25.0,142.0]	54.0 [24.0,102.0]	<0.001
D-Dimer (mg/L FEU)	727			1.1 [0.6,2.8]	0.9 [0.5,1.9]	0.019
Ferritin (ug/L)	501			815.0 [366.2,1499.0]	712.0 [357.5,1294.0]	0.341
Co-morbidities						
Stroke	0	74 (8.1)	22 (5.5)			0.117
TIA	0	12 (1.3)	5 (1.2)			0.87
Hypertension	0	315 (34.6)	144 (36.0)			0.674
Diabetes	0	267 (29.3)	106 (26.5)			0.326
AF	0	72 (7.9)	42 (10.5)			0.154
IHD	0	162 (17.8)	78 (19.5)			0.513
Heart Failure	0	61 (6.7)	34 (8.5)			0.299
COPD	0	74 (8.1)	32 (8.0)			0.977
Asthma	0	84 (9.2)	39 (9.8)			0.846
Cancer	0	64 (7.0)	21 (5.2)			0.278
Kidney disease	0	122 (13.4)	62 (15.5)			0.359
HIV	0	22 (2.4)	10 (2.5)			0.916
Solid organ transplant	0	25 (2.7)	19 (4.8)			0.092
Frailty	0	204 (22.4)	58 (14.5)			0.001

† p-value was from Kruskal-Wallis test for continuous variables and Chi-squared test for categorical variables.

Abbreviations: BMI: Body Mass Index, TIA: Transient Ischaemic Attack, IHD: Ischemic Heart Disease, COPD: Chronic Obstructive Pulmonary Disease, HIV: Human Immunodeficiency Virus, MAP:Mean Arterial Pressure, SPO2: Oxygen Saturation, GFR: Glomerular Filtration Rate, CRP: C-Reactive Protein, NLR: Neutrophils and Lymphocytes Ratio, NEWS2: National Early Warning Score 2.

For peer review only

Groups with non-Alpha and Alpha variants were not significantly different in median age (62yrs vs 64yrs, p=0.22) or ethnicity. The proportion of admissions who were female was larger in the group infected with the Alpha variant compared to those infected by non-Alpha variants (48.0% vs 41.8%, p=0.01).

Cases infected with the Alpha variant were less likely to be frail (14.5% vs 22.4% p=0.001). A higher proportion of those in the Alpha variant group were obese (30.2% v 24.8%, p=0.048). Other minor differences in comorbidities between groups are shown in Table 2, but did not reach statistical significance.

On admission a higher proportion of those infected with the Alpha variant were hypoxic (70.0% vs 62.5%, p=0.029), the main indicator of severe disease. CRP on admission was lower in the Alpha variant group (54 mg/L IQR: 24.0-102.0) compared to those infected with non-Alpha variants (70 mg/L, IQR: 25.0-142.0 p<0.001). Differences in other laboratory parameters did not meet either statistical or clinical significance.

Multivariate analysis of factors associated with severity of COVID-19 on admission

Multivariate logistic regression was applied to look at associations with severity of disease on admission as measured by hypoxia (Table 3), equivalent to WHO ordinal scale of ≥4 [12]. Age, sex, ethnicity, comorbidities and variant status (Alpha vs non-Alpha) were entered into the model. Severity of disease on admission, as measured by hypoxia, was the outcome variable. Age was a significant predictor of severity, with an odds ratio of 1.02 (CI 1.01-1.03, p<0.001) for hypoxia on admission for every advancing year. Obesity was associated with severity, giving an OR 1.70 (CI 1.28-2.26, p<0.001). Infection with the Alpha variant was also associated with increased hypoxia on admission (OR 1.68 CI: 1.26-2.24, p<0.001). Other variables were not significantly associated with hypoxia on admission, including sex, ethnicity and comorbidities.

For peer review only

Table 3: Odds ratios for severity (hypoxia) at admission from multivariate logistic regression model

	OR	p-value	95% CI
Age	1.02	<0.001	1.01 - 1.03
Male	0.96	0.75	0.73 - 1.25
Ethnicity			
non-White	1.15	0.35	0.86 - 1.55
Unknown	1.20	0.36	0.81 - 1.77
**Comorbidity**			
BMI>30	1.70	<0.001	1.28 - 2.26
Cardiovascular	0.79	0.15	0.58 - 1.09
Hypertension	1.11	0.52	0.81 - 1.51
Diabetes	0.75	0.07	0.55 - 1.02
Chronic respiratory disease	1.20	0.32	0.83 - 1.74
Cancer	0.60	0.06	0.35 - 1.02
Kidney disease	0.74	0.17	0.48 - 1.14
HIV	1.74	0.16	0.80 - 3.78
Organ transplant	0.79	0.55	0.37 -1.71
Frailty	0.96	0.85	0.64 - 1.45
Alpha variant	1.68	<0.001	1.26 - 2.24

Abbreviations: BMI: Body mass index, HIV: Human Immunodeficiency Virus

For peer review only

Comparison of non-sequenced and sequenced cases in wave two

We assessed for differences between the non-sequenced and sequenced inpatient cases to identify any possible bias in those that were sequenced. Demographics, admission physiological and laboratory parameters, and the outcome measure of hypoxia on admission are presented in Table 4. There was no significant difference of the proportion with the outcome measure, hypoxia on admission, in both the sequenced and non-sequenced inpatient group (47% vs 50%, p=0.381). There was no significant difference in the proportion of males in the sequenced group compared to the non-sequenced group (52.2% vs 53.8%, p=0.595), as with obesity (39.5% vs 38.4%, p=0.783) or the proportion of those from non-White ethnic backgrounds (41.4% vs 40.5%, p=0.934). On average, sequenced inpatient cases were significantly older (63 vs 57 years, p<0.001) and had a larger proportion of some comorbidities than non-sequenced cases.

For peer review only

Table 4: Patient characteristics of sequenced and non-sequenced inpatients in wave two.

	Non-sequenced	Sequenced	P-Value
n	1031	472	
Age (SD)	57.3 (21.0)	62.9 (19.9)	<0.001
Male (%)	538 (52.2)	254 (53.8)	0.595
Ethnicity (%)			0.934
White	418 (40.5)	194 (41.1)	
non-White	417 (40.4)	192 (40.7)	
Unknown	196 (19.0)	86 (18.2)	
**Comorbidities**			
BMI>30 (%)	302 (38.4)	139 (39.5)	0.783
Cardiovascular (%)	218 (21.1)	142 (30.1)	<0.001
Hypertension (%)	300 (29.1)	172 (36.4)	0.005
Diabetes (%)	269 (26.1)	127 (26.9)	0.787
Chronic respiratory Disease (%)	143 (13.9)	82 (17.4)	0.091
Cancer (%)	46 (4.5)	26 (5.5)	0.452
Kidney Disease (%)	116 (11.3)	74 (15.7)	0.021
HIV (%)	26 (2.5)	11 (2.3)	0.966
Organ transplant (%)	31 (3.0)	18 (3.8)	0.509
Frailty (%)	108 (10.5)	76 (16.1)	0.003
Hypoxia (%)	491 (47.6)	237 (50.2)	0.381

Abbreviations: BMI: Body mass index, HIV: Human Immunodeficiency Virus.

For peer review only

Discussion

Our data from a large, multi-site healthcare institution in one of the worst affected regions internationally provides a large dataset for in-depth comparison; for instance we report a similar number of cases as reported from a national observational cohort study from Japan [18]. Our hospitalised cohort shares similar demographics to other city populations in the UK, representative of London with around 40% of individuals from non-White ethnicities [19] . This compares to national population studies where the average age of cases was much lower and with lower proportion from non-White ethnciities [8,20].

There were threefold more SARS-CoV-2 RNA positive cases reported by the hospital laboratory in wave two. Partly this is attributed to increased testing capacity and changing testing strategy throughout 2020 (Supplementary Figure 1). Due to capacity limits, during wave one it was not local policy to offer testing to outpatients and those not requiring admission, instead relying on clinical diagnosis. Healthcare workers were not offered occupational health testing until the end of wave one. We therefore restricted comparison to inpatient and nosocomial cases.

There were almost twice as many admitted cases in wave two compared with wave one (1503 v 838). This is consistent with a higher local community incidence as reported by the ONS infection survey with 3.5% of individuals in London infected in January 2021 [21], compared with 2.2% of individuals in London at the peak of wave one [1]. The increase in peak hospital occupancy in wave two has also been reported nationally [22]. A major contributor to this increase in hospital admissions is likely to be the emergence of the Alpha variant, which is reported to be more transmissible [7].

Our finding is the first study in hospitalised cohorts to show increased severity of disease with the Alpha variant, as defined by hypoxia on admission which is equivalent to WHO ordinal scale of ≥4 [12] and a key marker of severe disease. The validity of using hypoxia as a marker of severity is

For peer review only

shown by the clinical characteristics of SARS-COV-2, with respiratory illness causing hypoxia in a minority of cases and with a smaller proportion having respiratory failure necessitating ventilation [23].Hypoxia on admission was chosen as a marker of severity to prevent confounding of results by changes in management of hospitalised patients across the pandemic. For instance treatment steroids, which were introduced during the study period around November 2020, have been shown to reduce risk of ventilation and death [24]. Other improvements in management, such as proning, anticoagulation and tocilizumab could also confound the outcomes of death and ICU admission. Hypoxia on admission is not at risk of confounding by changes in management of cases, as currently no significant management or treatment options are deployed in the community.

Our finding of increased sewverity with the Alpha variant is consistent with that reported in community studies, which show increased hospitalisation [20] and mortality [8] with a similar hazard to which we find here for hypoxia on admission. Notably however, these community studies failed to control for comorbidities [8, 20]. The association with severity we find persists even after adjustment for age, sex, and comorbidities. Moreover, testing in the first wave prior to emergence of the Alpha variant was strict due to limited testing capacity, potentially leading to an ascertainment bias towards more severe cases in the first wave. In comparison in the second wave testing was more widespread, potentially leading to increased ascertainment of less severe cases. This makes it even more striking that the association of the Alpha variant, which dominated the second wave, with severe disease is so prominent.

Notably the only other published study in hospital cohorts showed no difference in severity as measured by the composite outcome of need for ventilation or death [10]. Broadly the two cohorts from these hospital cohorts are similar, with an average age of around 60 and a high proportion of non-white ethnicities. In general this supports the external validity of our findings, however replication in dissimilar cohorts are awaited. The difference between findings in our study and those of [10] may be related to the choice of outcome. Our choice of outcome, hypoxia on admission, represents the natural history of disease prior to medical intervention as no treatments are currently deployed in

For peer review only

the community. The mortality outcome investigated by Frampton *et al,* is after hospital treatment which may ameliorate the severity increase that we find with the alpha variant, thereby explaining the differences in severity seen between our studies Interestingly, despite male sex being widely reported to be a risk factor for severe disease our multivariate model confirms findings by these authors that sex is not significantly associated with severity in hospitalised cohorts after adjusted analysis [10].

The lack of association between severity and male sex may corresponds to the increase in the proportion of females in the admitted cohort of wave two and those infected with Alpha, accounting for an extra 5% of admissions with SARS-CoV-2 infection. A study in press [25] suggests the Alpha variant may be more severe in hospitalised females, who may have increased mortality and/or requirement for ICU care. Our data, showing an increase in the proportion of females in the admission cohort and lack of expected association of severity with male sex is consistent with the finding that Alpha may show increased virulence in females.

We also included an assessment of bias by comparing characteristics of non-sequenced cases with those successfully sequenced. Whilst sequenced patients were older and more comorbid there was no significant difference between the proportion with the outcome measure of hypoxia on admission between our sequenced and non-sequenced cases. This suggests no significant bias towards severity in the sequenced group, which was predominantly made up of cases of the Alpha variant.

Admitted cases in wave two were also around half as likely to have a diagnosis of frailty, which may be due to fewer admissions from care homes during wave two, which has been reported both nationally [26] and internationally [27]. Additionally, admitted cases were around a third less likely to have cancer in wave two. Both of these reductions may also be as a result of individuals shielding, and therefore at reduced risk for acquiring SARS-CoV-2 infection. Other differences in comorbidities between waves were small and of unclear clinical significance.

For peer review only

One additional striking observation was the similarity in number of nosocomial cases in wave one (n=96 of 934 [10%] inpatient cases) and wave two (n=137 of 1640 [8%] inpatient cases). This incidence of nosocomial infection is a major challenge for UK healthcare institutions, with associated crude mortality at around 30% during the first wave [28,29]. Interestingly, nosocomial cases in wave one increased and started to fall before impact of the main infection control interventions of banning hospital visitors (March 25th), introducing universal surgical mask wearing (28th March 2020) and universal regular inpatient screening (after the first wave). In comparison, all these measures were in place prior to the second wave. The similar number of cases in wave two may in part be due to increased inpatient screening, which would identify asymptomatic cases, or introduction of the more transmissible Alpha variant which made up the vast majority of our sequenced nosocomial cases.

Some healthcare institutions report far fewer nosocomial acquisitions; for instance an academic hospital in Boston, USA reported only 2 nosocomial cases in over 9000 admissions [30]. This could be due to greater availability of side rooms for isolation or their use of N95 masks by HCWs, which may decrease transmission between HCWs and patients. In contrast, current UK public health policy recommends surgical facemasks for patient interactions unless performing aerosol generating procedures [31]. For this reason it will be important to further investigate the factors involved in nosocomial acquisition in both waves.

One limitation of our study is that the population comes from one city and findings therefore needs to be compared with findings in other regions. Our dataset included cases confirmed by SARS-CoV-2 RNA testing in our laboratory, so may miss those diagnosed only clinically. We could not compare outcomes after hospital admission, such as ICU admission or mortality, due to changes in in-hospital management between waves. In addition, we were unable to include some variables associated with severity in other studies due to few cases with these features (e.g. pregnancy) or due to poor coding in the dataset (e.g. liver disease), which prevents us from commenting on the risk associated with these variables.

For peer review only

The number of cases diagnosed, admissions and nosocomial cases were higher in wave two than wave one, likely due to the increased incidence caused by the more transmissible Alpha variant. Infection with the Alpha variant was associated with severity as measured by hypoxia on admission, the first such finding in hospitalised cohorts. Our findings support growing evidence that emerging variants may have altered virulence as well as increased transmissibility, with such evidence providing support for public health efforts to contain their spread. More broadly, it also increases understanding of the emergence of novel pathogens as they adapt to human hosts.

For peer review only

Contributions

LBS and WW were involved in the conceptualization, methodology, formal analysis of the synthesised data and writing (original drafting, review, and editing). TC, AA-M and GN were involved in investigation being responsible for whole genome sequencing and analysis of results. RB, FH and LdJ were involved in resources, administration and data curation. YW, JE, VC were involved in supervision, funding acquisition and drafting (review and editing). All authors agreed the final manuscript.

For peer review only

References

1 Edelstein M, Obi C, Chand M, *et al.* SARS-CoV-2 infection in London, England: changes to community point prevalence around lockdown time, March–May 2020. *J Epidemiol Community Health* Published Online First: 1 October 2020. doi:10.1136/jech-2020-214730

2 [dataset] UK Health Security Agency. Official UK Coronavirus Dashboard. https://coronavirus.data.gov.uk/details/healthcare (Accessed Mar 1, 2021).

3 Kirby T. New variant of SARS-CoV-2 in UK causes surge of COVID-19. *Lancet Respir Med* 2021;**9**:e20–1. doi:10.1016/S2213-2600(21)00005-9

4 Preliminary genomic characterisation of an emergent SARS-CoV-2 lineage in the UK defined by a novel set of spike mutations. 2020.https://virological.org/t/preliminary-genomic-characterisation-of-an-emergent-sars-cov-2-lineage-in-the-uk-defined-by-a-novel-set-of-spike-mutations/563 (accessed 24 Jan 2021).

5 Sparrow A, Carroll R, Pidd H. UK Covid: Boris Johnson says ‘great strides’ made with vaccinations but many are yet to be reached – as it happened. The Guardian. 2021.http://www.theguardian.com/politics/live/2021/feb/10/uk-covid-news-shapps-summer-holidays-abroad-travel-coronavirus-vaccine-live-latest-updates (accessed 1 Mar 2021).

6 Chand M, Others. Investigation of novel SARS-COV-2 variant: Variant of Concern 202012/01 (PDF). *Public Health England PHE* 2020.

7 Volz E, Mishra S, Chand M, *et al.* Transmission of SARS-CoV-2 Lineage B.1.1.7 in England: Insights from linking epidemiological and genetic data. *Medrxiv* 2021 doi:10.1101/2020.12.30.20249034

8 Davies NG, Jarvis CI, John Edmunds W, *et al.* Increased mortality in community-tested cases of SARS-CoV-2 lineage B.1.1.7. *Nature* 2021;**593**:270–4. doi:10.1038/s41586-021-03426-1

9 Challen R, Brooks-Pollock E, Read JM, *et al.* Risk of mortality in patients infected with SARS-CoV-2 variant of concern 202012/1: matched cohort study. *BMJ* 2021;**372**. doi:10.1136/bmj.n579

10 Frampton D, Rampling T, Cross A, *et al.* Genomic characteristics and clinical effect of the emergent SARS-CoV-2 B.1.1.7 lineage in London, UK: a whole-genome sequencing and hospital-based cohort study. *Lancet Infect Dis* Published Online First: 12 April 2021.

doi:10.1016/S1473-3099(21)00170-5

11 ECDC. Surveillance definitions for COVID-19. https://www.ecdc.europa.eu/en/covid-19/surveillance/surveillance-definitions (accessed 24 Jan 2021).

12 Print RB. COVID-19 Therapeutic Trial Synopsis. *World Health Organisation* 2020.https://www.who.int/publications/i/item/covid-19-therapeutic-trial-synopsis (accessed 8 Aug 2021).

13 Quick J. nCoV-2019 sequencing protocol v1 (protocols.io.bbmuik6w). protocols.io. 2020. doi:10.17504/protocols.io.bbmuik6w

14 Artic Network. nCoV-2019 novel coronavirus bioinformatics protocol https://artic.network/ncov-2019/ncov2019-bioinformatics-sop.html (accessed 24 Jan 2021).

15 O'Toole Á, Scher E, Underwood A, et al. Assignment of epidemiological lineages in an emerging pandemic using the pangolin tool. Virus Evol 2021;7. doi:10.1093/ve/veab064

16 Rambaut A, Loman N, Pybus O, *et al.* C.-19 GC UK, Preliminary genomic characterisation of an emergent SARS-CoV-2 lineage in the UK defined by a novel set of spike mutations. virological. org (2020).

17 King's College London. Acknowledging - Rosalind research computing infrastructure. https://rosalind.kcl.ac.uk/acknowledging/ (accessed 6 Mar 2021).

18 Saito S, Asai Y, Matsunaga N, *et al.* First and second COVID-19 waves in Japan: A comparison of disease severity and characteristics. Journal of Infection. 2020. doi:10.1016/j.jinf.2020.10.033

19 White E. Ethnicity and National Identity in England and Wales - Office for National Statistics. 2012.https://www.ons.gov.uk/peoplepopulationandcommunity/culturalidentity/ethnicity/articles/ethnicityandnationalidentityinenglandandwales/2012-12-11 (accessed 18 Jun 2021).

20 Nyberg T, Twohig KA, Harris RJ, *et al.* Risk of hospital admission for patients with SARS-CoV-2 variant B.1.1.7: cohort analysis. *BMJ* 2021;**373**. doi:10.1136/bmj.n1412

21 Steel, K & Donnarumma, H. Coronavirus (COVID-19) Infection Survey, UK. Office for National Statistics. 2021.https://www.ons.gov.uk/peoplepopulationandcommunity/healthandsocialcare/conditionsanddiseases/bulletins/coronaviruscovid19infectionsurveypilot/8january2021 (accessed 1 Feb 2021).

22 NHS England Hospitals admit one third of COVID patients in a single month. https://www.england.nhs.uk/2021/02/hospitals-admit-one-third-of-covid-patients-in-a-single-month/ (accessed 1 Mar 2021).

23 Wu Z, McGoogan JM. Characteristics of and important lessons from the coronavirus disease 2019 (COVID-19) outbreak in China: summary of a report of 72 314 cases from the Chinese Center for Disease Control and Prevention. *JAMA* 2020;**323**:1239–42.https://jamanetwork.com/journals/jama/article-abstract/2762130

24 RECOVERY Collaborative Group, Horby P, Lim WS, *et al.* Dexamethasone in Hospitalized Patients with Covid-19 - Preliminary Report. *N Engl J Med* Published Online First: 17 July 2020. doi:10.1056/NEJMoa202143625 Stirrup OT, Boshier FAT, Venturini C, et al. SARS-CoV-2 lineage B.1.1.7 is associated with greater disease severity among hospitalised women but not men. medRxiv; 2021. DOI: 10.1101/2021.06.24.21259107.

26 [dataset] John S. Deaths involving COVID-19 in the care sector, England and Wales - Office

For peer review only - http://bmjopen.bmj.com/site/about/guidelines.xhtml

for National Statistics. 2020. https://www.ons.gov.uk/peoplepopulationandcommunity/birthsdeathsandmarriages/deaths/articles/deathsinvolvingcovid19inthecaresectorenglandandwales/deathsoccurringupto12june2020andregisteredupto20june2020provisional (accessed 25 Feb 2021).

27 Ioannidis JPA, Axfors C, Contopoulos-Ioannidis DG. Second versus first wave of COVID-19 deaths: shifts in age distribution and in nursing home fatalities. *medRxiv* 2020;:2020.11.28.20240366. doi:10.1101/2020.11.28.20240366

28 Carter B, Collins JT, Barlow-Pay F, *et al.* Nosocomial COVID-19 infection: examining the risk of mortality. The COPE-Nosocomial Study (COVID in Older PEople). *J Hosp Infect* 2020;**106**:376–84. doi:10.1016/j.jhin.2020.07.013

29 Snell LB, Fisher CL, Taj U, Stirrup O, Merrick B, Alcolea-Medina A, Charalampous T, Signell AW, Wilson HD, Betancor G, Kia Ik MT, Cunningham E, Cliff PR, Pickering S, Galao RP, Batra R, Neil SJD, Malim MH, Doores KJ, Douthwaite ST, Nebbia G; COVID-19 Genomics UK (COG-UK) consortium. Combined epidemiological and genomic analysis of nosocomial SARS-CoV-2 infection early in the pandemic and the role of unidentified cases in transmission. Clin Microbiol Infect. 2021 Aug 13:S1198-743X(21)00435-3. doi: 10.1016/j.cmi.2021.07.040. Epub ahead of print

30 Rhee C, Baker M, Vaidya V, *et al.* Incidence of Nosocomial COVID-19 in Patients Hospitalized at a Large US Academic Medical Center. *JAMA Netw Open* 2020;**3**:e2020498. doi:10.1001/jamanetworkopen.2020.20498

31. Public Health England. COVID-19: infection prevention and control (IPC). GOV.UK; Available: https://www.gov.uk/government/publications/wuhan-novel-coronavirus-infection-prevention-and-control [Published Jan 10, 2021; Accessed Mar 15, 2021].

For peer review only

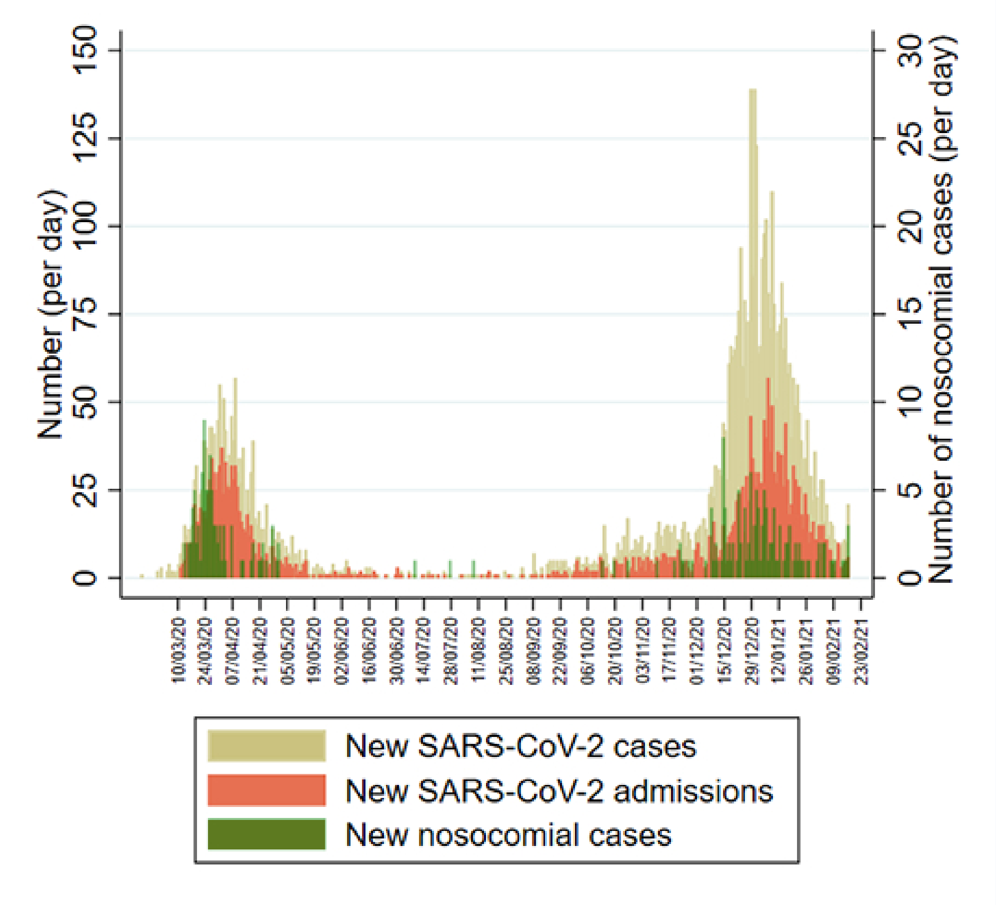

Distribution of laboratory confirmed SARS-CoV-2 cases over time. Daily incidence of new cases (beige), newly admitted cases (orange) and nosocomial acquisitions (green) over time.

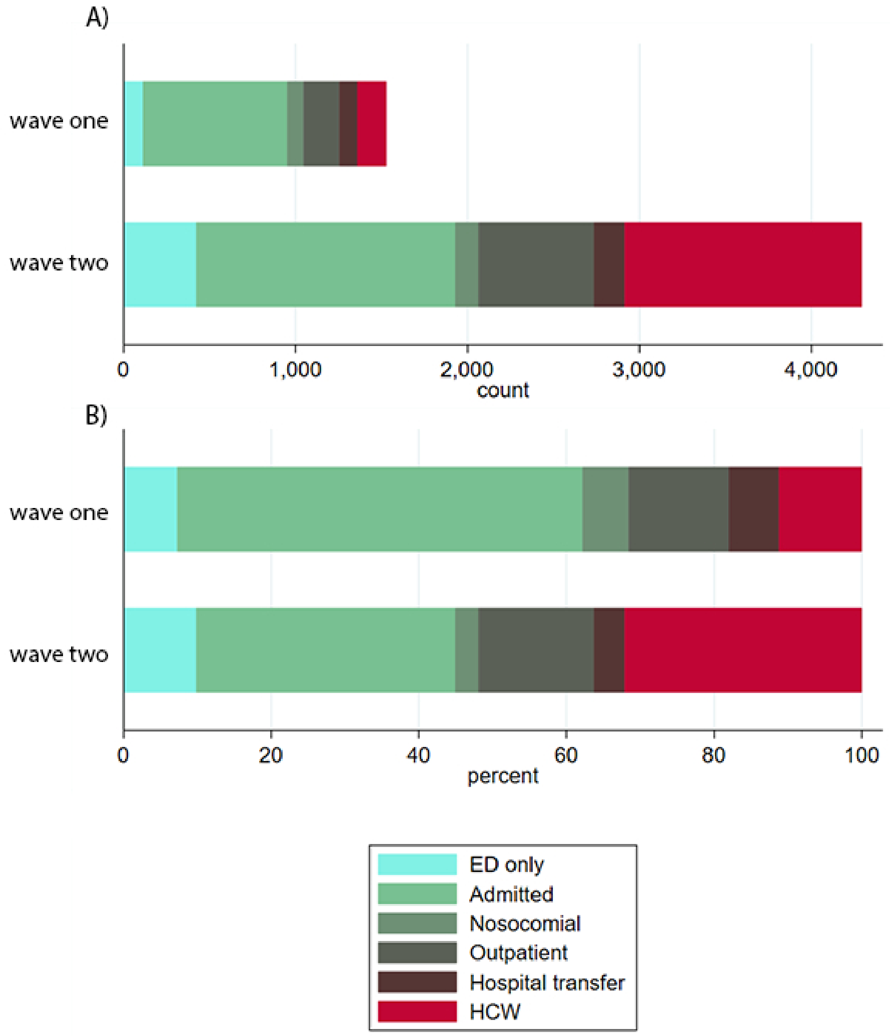

(A) Absolute number of cases within the different hospital cohorts during wave one (upper) and wave two (lower). (B) Proportion of cases within the different hospital cohorts during wave one (upper) and wave two (lower).

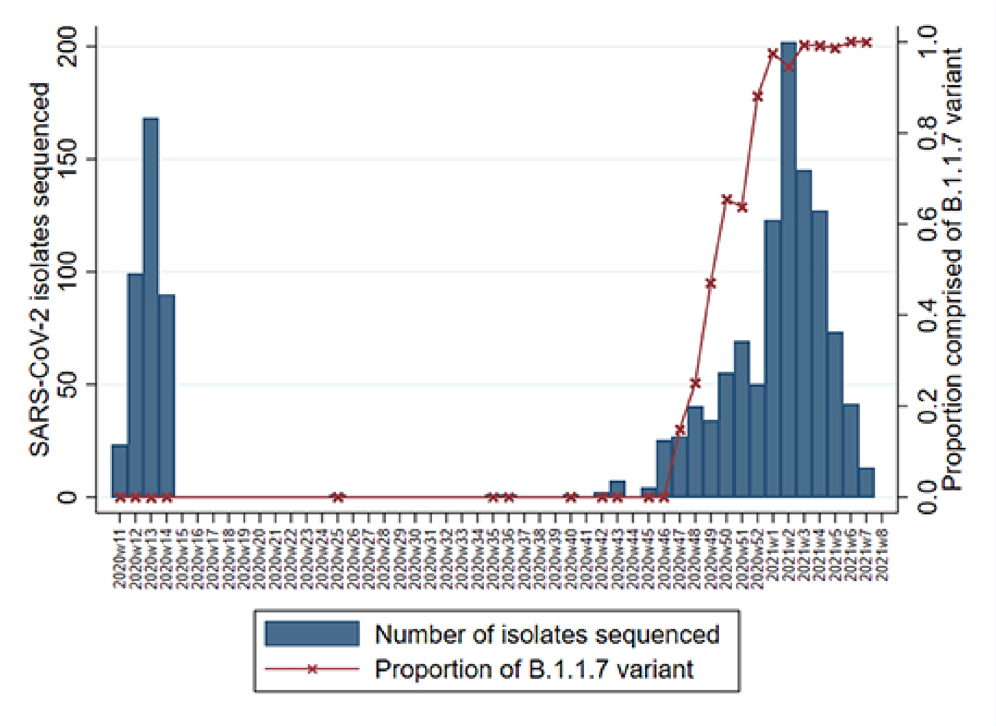

Number of cases with sequenced SARS-CoV-2 isolates by epi-week (bar) and the proportion of which were made up of the alpha variant B.1.1.7 (red line)

190x138mm (72 x 72 DPI)

Supplementary Material

Risk factors for severe disease from COVID-19 - lIterature review

Risk factor	References	Comment on inclusion in multivariable model
Age	[1–5]	Included as a continuous variable.
Sex	[2,5]	Included as binary variable.
Co-morbiidities<br>1. Cancer<br>2. Chronic kidney disease<br>3. Chronic lung disease<br>4. Dementia<br>5. DIabetes<br>6. Cardiac disease<br>7. HIV<br>8. Immunocompromise<br>9. Liver disease<br>10. Obesity<br>11. Pregnancy<br>12. Transplant<br>13. Stroke<br>14. Frailty	1. [2,3,5–7]<br>2. [2,4,6]<br>3. [2,3,5,6]<br>4. [5,6]<br>5. [2,3,6]<br>6. [2,4–6]<br>7. [6,8]<br>8. [2,6]<br>9. [2,5,6]<br>10. [4,6,9–11]<br>11. [6,12]<br>12. [2,6]<br>13. [2,6]<br>14. [13–15]	1. Included<br>2. Included<br>3. Included<br>4. Poorly coded in our dataset so not included.<br>5. Included<br>6. Included<br>7. Included<br>8. Poorly coded in our dataset so not included.<br>9. Poorly coded in our dataset so not included<br>10. Included<br>11. Small number of pregnant individuals in our dataset so not included<br>12. Included<br>13. Included with cardiovascular disease
Ethnicity	[2,5,16,17]	Evidence suggests individuals of non-White ethnicities are at increased risk in multiple territories. Included as categorical variable.
Socioeconomic background	[18,19]	These data suggest risk posed by certain ethnicity may be related to socioeconomic background. For simplicity we included only ethnicity variable.
Variant status	[20–22]	Alpha variant associated with increased mortality in population level studies, but not in single hospitalised study (full discussion in main text)

For peer review only

REFERENCES

1 Richardson S, Hirsch JS, Narasimhan M, *et al.* Presenting Characteristics, Comorbidities, and Outcomes Among 5700 Patients Hospitalized With COVID-19 in the New York City Area. *JAMA* 2020;**323**:2052–9.

2 Williamson EJ, Walker AJ, Bhaskaran K, *et al.* Factors associated with COVID-19-related death using OpenSAFELY. *Nature* 2020;**584**:430–6.

3 Wu Z, McGoogan JM. Characteristics of and Important Lessons From the Coronavirus Disease 2019 (COVID-19) Outbreak in China: Summary of a Report of 72 314 Cases From the Chinese Center for Disease Control and Prevention. *JAMA* 2020;**323**:1239–42.

4 Petrilli CM, Jones SA, Yang J, *et al.* Factors associated with hospital admission and critical illness among 5279 people with coronavirus disease 2019 in New York City: prospective cohort study. *BMJ* 2020;**369**:m1966.

5 Harrison SL, Fazio-Eynullayeva E, Lane DA, *et al.* Comorbidities associated with mortality in 31,461 adults with COVID-19 in the United States: A federated electronic medical record analysis. *PLoS Med* 2020;**17**:e1003321.

6 Know WYNT. People with Certain Medical Conditions. https://www.cdc.gov/coronavirus/2019-ncov/need-

For peer review only - http://bmjopen.bmj.com/site/about/guidelines.xhtml

extra-precautions/people-with-medical-conditions.html

7 Dai M, Liu D, Liu M, *et al.* Patients with Cancer Appear More Vulnerable to SARS-CoV-2: A Multicenter Study during the COVID-19 Outbreak. *Cancer Discov* 2020;**10**:783–91.

8 Geretti AM, Stockdale AJ, Kelly SH, *et al.* Outcomes of COVID-19 related hospitalization among people with HIV in the ISARIC WHO Clinical Characterization Protocol (UK): a prospective observational study. *Clin Infect Dis* Published Online First: 23 October 2020. doi:10.1093/cid/ciaa1605

9 Lighter J, Phillips M, Hochman S, *et al.* Obesity in Patients Younger Than 60 Years Is a Risk Factor for COVID-19 Hospital Admission. *Clin Infect Dis* 2020;**71**:896–7.

10 Tartof SY, Qian L, Hong V, *et al.* Obesity and Mortality Among Patients Diagnosed With COVID-19: Results From an Integrated Health Care Organization. *Ann Intern Med* 2020;**173**:773–81.

11 Kompaniyets L, Goodman AB, Belay B, *et al.* Body Mass Index and Risk for COVID-19-Related Hospitalization, Intensive Care Unit Admission, Invasive Mechanical Ventilation, and Death - United States, March-December 2020. *MMWR Morb Mortal Wkly Rep* 2021;**70**:355–61.

12 Allotey J, Stallings E, Bonet M, *et al.* Clinical manifestations, risk factors, and maternal and perinatal outcomes of coronavirus disease 2019 in pregnancy: living systematic review and meta-analysis. *BMJ* 2020;**370**:m3320.

13 Dumitrascu F, Branje KE, Hladkowicz ES, *et al.* Association of frailty with outcomes in individuals with COVID-19: A living review and meta-analysis. *J Am Geriatr Soc* 2021;**69**:2419–29.

14 Kastora S, Kounidas G, Perrott S, *et al.* Clinical frailty scale as a point of care prognostic indicator of mortality in COVID-19: a systematic review and meta-analysis. *EClinicalMedicine* 2021;**36**:100896.

15 Yang Y, Luo K, Jiang Y, *et al.* The Impact of Frailty on COVID-19 Outcomes: A Systematic Review and Meta-analysis of 16 Cohort Studies. *J Nutr Health Aging* 2021;**25**:702–9.

16 Price-Haywood EG, Burton J, Fort D, *et al.* Hospitalization and Mortality among Black Patients and White Patients with Covid-19. *N Engl J Med* 2020;**382**:2534–43.

17 Gold JAW, Rossen LM, Ahmad FB, *et al.* Race, Ethnicity, and Age Trends in Persons Who Died from COVID-19 - United States, May-August 2020. *MMWR Morb Mortal Wkly Rep* 2020;**69**:1517–21.

18 Kabarriti R, Brodin NP, Maron MI, *et al.* Association of Race and Ethnicity With Comorbidities and Survival Among Patients With COVID-19 at an Urban Medical Center in New York. *JAMA Netw Open* 2020;**3**:e2019795.

19 Muñoz-Price LS, Nattinger AB, Rivera F, *et al.* Racial Disparities in Incidence and Outcomes Among Patients With COVID-19. *JAMA Netw Open* 2020;**3**:e2021892.

20 Davies NG, Jarvis CI, CMMID COVID-19 Working Group, *et al.* Increased mortality in community-tested cases of SARS-CoV-2 lineage B.1.1.7. *Nature* 2021;**593**:270–4.

21 Frampton D, Rampling T, Cross A, *et al.* Genomic characteristics and clinical effect of the emergent SARS-CoV-2 B.1.1.7 lineage in London, UK: a whole-genome sequencing and hospital-based cohort study. *Lancet Infect Dis* Published Online First: 12 April 2021. doi:10.1016/S1473-3099(21)00170-5

22 Challen R, Brooks-Pollock E, Read JM, *et al.* Risk of mortality in patients infected with SARS-CoV-2 variant of concern 202012/1: matched cohort study. *BMJ* 2021;**372**:n579.

For peer review only

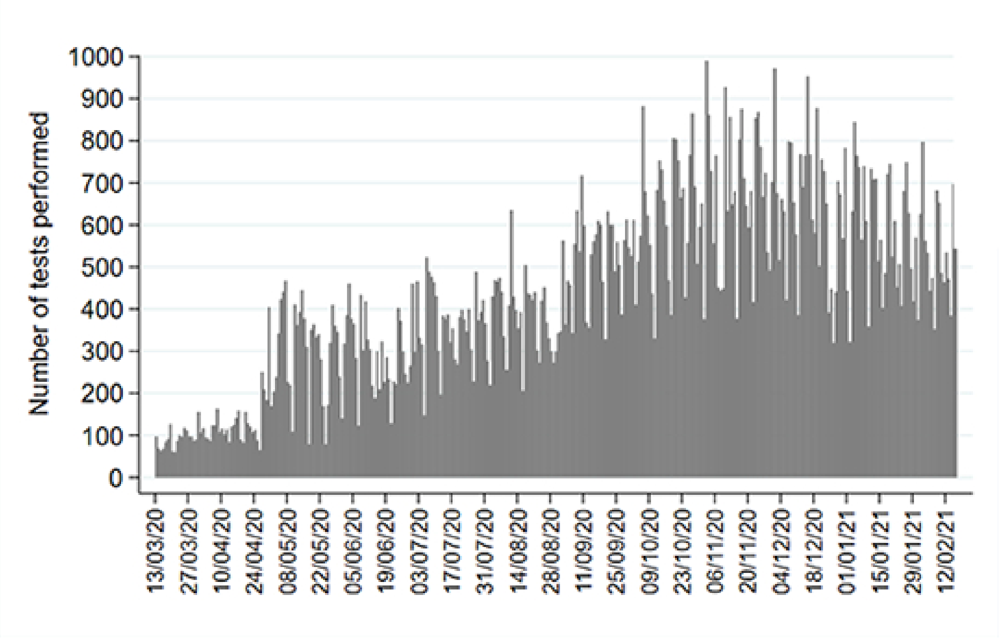

Our laboratory began testing on 13th March 2020 with initial capacity for around 150 PCR tests per day, before increasing to around 500 tests per day in late April during wave one, and up to 1000 tests per day during wave two

190x125mm (72 x 72 DPI)

Appendix - List of COG-UK HOCI Investigators

Barts site

Name of individual	Employing Institution	Role on HOCI study
**Teresa Cutino-Moguel**	**Barts Heath NHS Trust**	**PI Barts Health**
Tabassum Khan	Barts Heath NHS Trust	Research assistant
Beatrix Kele	Barts Heath NHS Trust	Sequencing scientist
Raghavendran Kulasegaran-Shylini	Barts Heath NHS Trust	Sequencing scientist
Claire E. Broad	Barts Heath NHS Trust	Sequencing scientist
Dola Owoyemi	Barts Heath NHS Trust	Sequencing scientist
David Harrington	Barts Heath NHS Trust	Infection Control Doctor
Clare Coffey	Barts Heath NHS Trust	Infection Control nurse
Martina Cummins	Barts Heath NHS Trust	Infection Control nurse
Anna Riddell	Barts Heath NHS Trust	Virology Consultant
Tyrra D'Souza	Barts Heath NHS Trust	Research Assistant

Glasgow site

Name of individual	Employing Institution	Role on HOCI study
Guy Mollett	MRC-University of Glasgow Centre for Virus Research	Clinical Research Fellow
**Emma Thomson**	**MRC-University of Glasgow Centre for Virus Research and NHS Greater Glasgow and Clyde**	**Principal Investigator**
Christine Peters	NHS Greater Glasgow and Clyde	Microbiology Consultant
Aleks Marek	NHS Greater Glasgow and Clyde	Infection Control Lead/Microbiology Consultant
Rory Gunson	NHS Greater Glasgow and Clyde	Virology laboratory lead
Emily Goldstein	NHS Greater Glasgow and Clyde	Sample extraction
Emilie Shepherd	NHS Greater Glasgow and Clyde	Sample extraction
James Shepherd	MRC-University of Glasgow Centre for Virus Research	Clinical Research Fellow
David Robertson	MRC-University of Glasgow Centre for Virus Research	Lead bioinformatician
Katherine Smollett	MRC-University of Glasgow Centre for Virus Research	Sequencing
Ana da Silva Filipe	MRC-University of Glasgow Centre for Virus Research	Sequencing
Alice Broos	MRC-University of Glasgow Centre for Virus Research	Sequencing
Stephen Carmichael	MRC-University of Glasgow Centre for Virus Research	Sequencing
Nicholas Suarez	MRC-University of Glasgow Centre for Virus Research	Sequencing

For peer review only

Chris Davis	MRC-University of Glasgow Centre for Virus Research	Sample extraction
Sreenu Vattipally	MRC-University of Glasgow Centre for Virus Research	Bioinformatician
Joseph Hughes	MRC-University of Glasgow Centre for Virus Research	Bioinformatician
Ioulia Tsatsani	MRC-University of Glasgow Centre for Virus Research	Bioinformatician
Jacqueline McTaggart	NHS Greater Glasgow and Clyde	Research Nurse
Stephanie McEnhill	NHS Greater Glasgow and Clyde	Research Nurse

Guy's and St Thomas' site

Name of individual	Employing Institution	Role on HOCI study
Adela Medina	Viapath	Sequence
Themoula Charalampous	KCL	Sequence
Bindi Patel	GSTT NHS Trust	Sequence
Flavia Flaviani	GSTT NHS Trust	Bioinformatics
Jörg Saßmannshausen	GSTT NHS Trust	Bioinformatics/IT
May Rabuya	GSTT NHS Trust	Research Nurse-data collection
Sulekha Gurung	GSTT NHS Trust	Research Nurse-data collection
Anu Augustine	GSTT NHS Trust	Research Nurse-data collection
Rahul Batra	GSTT NHS Trust	Sequencing/IT/manager
Luke Snell	GSTT NHS Trust	Sequence, bioinf, data collection, IPC
**Gaia Nebbia**	**GSTT NHS Trust**	**Principal Investigator**

Imperial site

Name of individual	Employing Institution	Role on HOCI study
**Alison Holmes**	**Imperial Healthcare NHS Trust**	**Principal Investigator**
Sid Mookerjee	Imperial Healthcare NHS Trust	Data lead
James Price	Imperial Healthcare NHS Trust	Site IPC Lead
Paul Randell	Imperial Healthcare NHS Trust	Laboratory Lead
Krystal Johnson	Imperial Healthcare NHS Trust	Research Nurse
Thilipan Thaventhiran	Imperial Healthcare NHS Trust	Research Nurse
Damien Mine	Imperial Healthcare NHS Trust	Clinician
Sophie Hunter	Imperial Healthcare NHS Trust	Research Nurse
Isa Ahmad	Imperial Healthcare NHS Trust	Data Analyst
Anitha Ramanathan	Imperial Healthcare NHS Trust	Research Nurse

For peer review only

Liverpool site

Name of individual	Employing Institution	Role on HOCI study
**Anu Chawla**	**Liverpool NHS Foundation Trust**	**Principal Investigator**
Alistair Derby	University of Liverpool	Sequencing lab lead
Sam Haldenby	University of Liverpool	Bioinformatics lead
Becky Taylor	Liverpool NHS Foundation Trust	Research data coordinator
Keith Morris	Liverpool NHS Foundation Trust	Research nurse
Charles Numbere	Liverpool NHS Foundation Trust	Healthcare assistant
Mark Hopkins	Liverpool NHS Foundation Trust	Consultant clinical scientist
Jenifer Mason	Liverpool NHS Foundation Trust	Consultant microbiologist
Alexandra Bailey	Liverpool NHS Foundation Trust	Research administrator
Debbie Lankstead	Liverpool NHS Foundation Trust	Assistant Director of Infection Control
Damian Burns	Liverpool NHS Foundation Trust	Infection Control Nurse

Manchester site

Name of individual	Employing Institution	Role on HOCI study
**Nicholas Machin**	**PHE and MFT**	**Principal Investigator**
Shazaad Ahmad	MFT	Consultant Virologist and IPC Doctor: review of sequencing reports
Julie Cawthorne	MFT	Clinical Director of Infection Prevention and Control: review of sequencing reports and assistance with CRF completion
Ryan George	MFT	IPC surveillance officer: co-ordination of metadata and sequencing reports
James Montgomery	MFT	IPC Nurse: review of sequencing reports and implementation of IPC actions
Deborah McKew	MFT	IPC Nurse: review of sequencing reports and implementation of IPC actions

Newcastle site

Name of individual	Employing Institution	Role on HOCI study
**Yusri Taha**	**Newcastle NHS Trust**	**Site PI**
Angela Cobb	Newcastle NHS Trust	IPC matron
Michelle Ramsay	Newcastle NHS Trust	Infection Control
Maria Leader	Newcastle NHS Trust	Infection Control
Shirelle Burton-Fanning	Newcastle NHS Trust	Virologist
Julie Samuel	Newcastle NHS Trust	Microbiologist and IPC doctor

For peer review only

Sarah Francis	Newcastle NHS Trust	Trial coordinator
Lydia Taylor	Newcastle NHS Trust	Trial's Research Nurse
Darren Smith	Northumbria University	Lead, sequencing
Matthew Bashton	Northumbria University	Bioinformatics lead
Matthew Crown	Northumbria University	Bioinformatics scientist

Nottingham site

Name of individual	Employing Institution	Role on HOCI study
**Nikunj Mahida**	**Nottingham NHS Trust**	**Principal Investigator**
Matthew Loose	University of Nottingham	Sequencing/Bioinfomatics
Patrick McClure	University of Nottingham	Sequencing/Bioinfomatics
Mitch Clarke	Nottingham NHS Trust	IPC - IPC Lead - review of cases, sequencing data
Elaine Baxter	Nottingham NHS Trust	IPC - Senior IPC team member, review of cases, sequencing data
Carl Yates	Nottingham NHS Trust	IPC - Senior IPC team member, review of cases, sequencing data
Irfan Aslam	Nottingham NHS Trust	Data Entry
Vicki Fleming	Nottingham NHS Trust	Sample collection and processing
Michelle Lister	Nottingham NHS Trust	Sample collection and processing
Johnny Debebe	University of Nottingham	Bioinformatics
Nadine Holmes	University of Nottingham	Sequencing
Christopher Moore	University of Nottingham	Sequencing
Matt Carlile	University of Nottingham	Sequencing

Royal Free site

Name of individual	Employing Institution	Role on study
**Tabitha Mahungu**	**Royal Free London NHS Trust**	**Principal Investigator**
Sophie Weller	Royal Free London NHS Trust	Sub-Investigator
Tanzina Haque	Royal Free London NHS Trust	Sub-Investigator
Jennifer Hart	Royal Free London NHS Trust	Sub-Investigator
Dianne Irish-Tavares	Royal Free London NHS Trust	Sub-Investigator
Eric Witele	Royal Free London NHS Trust	Clinical Research Nurse
Mia De Mesa	Royal Free London NHS Trust	Clinical Research Nurse
Vicky Pang	Royal Free London NHS Trust	Head of Infection Prevention & Control Nursing – provided IPC data for CRFs
Jelena Heaphy	Royal Free London NHS Trust	Clinical Lead Nurse Infection Prevention and Control - provided IPC data for CRFs

For peer review only

Wendy Chatterton	Health Services Laboratory	Virology Service Manager, Organised samples & Logistics
Monika Pusok	Health Services Laboratory	Medical laboratory assistant , Organised samples & Logistics

Sandwell site

Name of individual	Employing Institution	Role on HOCI study
**Dr Tranprit Saluja**	**Sandwell & West Birmingham Hospitals NHS Trust**	**Principal Investigator - Consultant Microbiologist and IPC doctor**
Zahira Maqsood	Sandwell NHS Trust	Clinical Research Practitioner
Angie Williams	Sandwell NHS Trust	Research Data Coordinators.
Debbie Devonport	Sandwell NHS Trust	Research Data Coordinators.
Lucy Palinkas	Sandwell NHS Trust	Infection control Data Analyst
Diane Thomlinson	Sandwell NHS Trust	Infection control Nurse
Julie Booth	Sandwell NHS Trust	Lead Nurse IPC
Ashok Dadrah	Sandwell NHS Trust	Laboratory Services Manager
Amanda Symonds	Sandwell NHS Trust	Senior Biomedical Scientist (Microbiology
Cassandra Craig	Sandwell NHS Trust	Laboratory Associate Practitioner
Dr Abhinav Kumar	Sandwell NHS Trust	Consultant microbiologist

Sheffield site

Name of individual	Employing Institution	Role on HOCI study
**Thushan de Silva**	**University of Sheffield**	**Principal Investigator**
Matthew D Parker	University of Sheffield	Bioinformatics processing/management
Peijun Zhang	University of Sheffield	WGS
Max Whiteley	University of Sheffield	WGS
Benjamin B Lindsey	University of Sheffield	WGS
Paige Wolverson	University of Sheffield	WGS
Benjamin H Foulkes	University of Sheffield	WGS
Luke Green	University of Sheffield	WGS
Marta Gallis Ramalho	University of Sheffield	WGS
Stavroula F Louka	University of Sheffield	WGS
Adrienn Angyal	University of Sheffield	WGS
Nikki Smith	University of Sheffield	Management/admin
David G Partridge	Sheffield NHS Trust	Investigator
Cariad Evans	Sheffield NHS Trust	Investigator
Mohammad Raza	Sheffield NHS Trust	Investigator
Hayley Colton	Sheffield NHS Trust	Investigator

Rebecca Gregory	Sheffield NHS Trust	Clinical trial assistant
Phillip Ravencroft	Sheffield NHS Trust	Clinical trial assistant
Katie Johnson	Sheffield NHS Trust	Sample collection and processing
Sharon Hsu	University of Sheffield	Bioinformatics support
Alexander J Keeley	Sheffield NHS Trust	
Alison Cope	Sheffield NHS Trust	
Amy State	Sheffield NHS Trust	Sample collection and processing
Nasar Ali	Sheffield NHS Trust	
Rasha Raghei	Sheffield NHS Trust	
Joe Heffer	Sheffield NHS Trust	
Stella Christou	University of Sheffield	WGS
Samantha E Hansford	University of Sheffield	Management/admin
Hailey R Hornsby	University of Sheffield	WGS
Phil Wade	Sheffield NHS Trust	Data collection
Kay Cawthron	Sheffield NHS Trust	Data collection
Maqsood Khan	Sheffield NHS Trust	Data collection
Amber Ford	Sheffield NHS Trust	Data input
Imogen Wilson	Sheffield NHS Trust	Data input
Kate Harrington	Sheffield NHS Trust	Sample collection
Nic Tinker	Sheffield NHS Trust	Sample collection
Sally Nyinza	Sheffield NHS Trust	Investigator

Southampton site

Name of individual	Employing Institution	Role on study
**Kordo Saeed**	**Southampton NHS Trust**	**Principal Investigator**
Jacqui Prieto	Southampton NHS Trust	Samples/logistics
Adhyana Mahanama	Southampton NHS Trust	Samples/logistics
Buddhini Samaraweera	Southampton NHS Trust	Samples/logistics
Siona Silviera	Southampton NHS Trust	Samples/logistics
Emanuela Pelosi	Southampton NHS Trust	Samples/logistics
Eleri Wilson-Davies	Southampton NHS Trust	Samples/logistics
Sarah Jeremiah	Southampton NHS Trust	Data collection
Helen Wheeler	Southampton NHS Trust	Data collection
Matthew Harvey	Southampton NHS Trust	Data collection
Thea Sass	Southampton NHS Trust	Data collection
Helen Umpleby	Southampton NHS Trust	Data collection
Stephen Aplin	Southampton NHS Trust	Data collection
Samuel Robson	Portsmouth University	Sequencing lead
Sharon Glaysher	Portsmouth Hospital NHS Trust	Sequencing
Scott Elliott	Portsmouth Hospital NHS Trust	Sequencing
Kate Cook	Portsmouth University	Sequencing
Christopher Fearn	Portsmouth University	Sequencing
Salman Goudarzi	Portsmouth University	Sequencing
Katie Loveson	Portsmouth University	Sequencing

For peer review

St George's site

Name of individual	Employing Institution	Role on HOCI study
Kenneth Laing	St Georges, UoL	Sequencing
Irene Monahan	St Georges, UoL	Sequencing
Adam Witney	St Georges, UoL	Bioinformatician
Joshua Taylor	St Georges NHS Trust	Virology, data collection, CRF completion and upload to MACRO
NgeeKeong Tan	St Georges NHS Trust	Virology, data collection, CRF completion and upload to MACRO
**Cassie Pope**	**St Georges NHS Trust and St Georges, UoL**	**PI, data collection, CRF completion and upload to Macro**
Claudia Cardosa Pereira	St Georges NHS Trust	IPC nurse
Vaz Malik	St Georges, UoL	Upload to macro

UCLH site

Name of individual	Employing Institution	Role on HOCI study
**Gee Yen Shin**	**UCLH NHS Trust**	**Principal Investigator, virologist**
Eleni Nastouli	UCLH NHS Trust	Virologist
Catherine Houlihan	UCLH NHS Trust	Virologist
Judith Heaney	UCLH NHS Trust	Clinical scientist
Matt Byott	UCLH NHS Trust	Bioinformatician
Dan Frampton	UCL / UCLH	Bioinformatician
Gema Martinez-Garcia	UCLH NHS Trust	Senior infection control nurse
Leila Hail	UCLH NHS Trust	Senior infection control nurse
Ndifreke Atang	UCLH NHS Trust	Clinical trials practitioner
Helen Francis	UCLH NHS Trust	Research nurse
Milica Rajkov	UCLH NHS Trust	Clinical trials co-ordinator

UCL Genomics

Name of individual	Employing Institution	Role on HOCI study
**Judith Breuer**	**UCL**	**Chief Investigator**
Rachel Williams	UCL	Sequencing
Sunando Roy	UCL	Sequencing
Charlotte Williams	UCL	Sequencing
Nadua Bayzid	UCL	Sequencing
Marius Cotic	UCL	Sequencing

UCL Comprehensive Clinical Trials Unit

Name of individual	Employing Institution	Role on HOCI study
James Blackstone	UCL	Project Manager
Leanne Hockey	UCL	Trial Manager

For peer review only

Alyson MacNeil	UCL	Trial Manager
Rachel McComish	UCL	Data Analyst
Monica Panca	UCL	Health Economist
Georgia Marley	UCL	Data Manager

UCL Institute for Global Health

Name of individual	Employing Institution	Role on HOCI study
Andrew Copas	UCL	Senior Statistician
Oliver Stirrup	UCL	Statistician
Fiona Mapp	UCL	Qualitative Researcher

UCL Research IT Services

Name of individual	Employing Institution	Role on HOCI study
Alif Tamuri	UCL	IT Developer
Stefan Piatek	UCL	IT Developer

University of Strathclyde

Name of individual	Employing Institution	Role on HOCI study
Paul Flowers	UoS	Senior Qualitative Researcher

Francis Crick Institute

Name of individual	Employing Institution	Role on HOCI study
Marg Crawford	Francis Crick Institute	Sample processing/sequencing
Laura Cubitt	Francis Crick Institute	Sample processing/sequencing
Deborah J Jackson	Francis Crick Institute	Sample processing/sequencing
Jimena Perez-Lloret	Francis Crick Institute	Sample processing/sequencing
Sophie Ward	Francis Crick Institute	Sample processing/sequencing
Makis Fidanis	Francis Crick Institute	Sample processing/sequencing
Aaron Sait	Francis Crick Institute	Sample processing/sequencing
Robert Goldstone	Francis Crick Institute	Data Processing
Harshil Patel	Francis Crick Institute	Data Processing
Chelsea Sawyer	Francis Crick Institute	Data Processing
Aengus Stewart	Francis Crick Institute	Data Processing
Steve Gamblin	Francis Crick Institute	Methodology/Supervision
Charles Swanton	Francis Crick Institute	Methodology/Supervision
Jerome Nicod	Francis Crick Institute	Methodology/Supervision

For peer review only

STROBE 2007 (v4) Statement—Checklist of items that should be included in reports of *cohort studies*

Section/Topic	Item #	Recommendation	Reported on page #
**Title and abstract**	1	(*a*) Indicate the study's design with a commonly used term in the title or the abstract	2
		(*b*) Provide in the abstract an informative and balanced summary of what was done and what was found	2
**Introduction**			
Background/rationale	2	Explain the scientific background and rationale for the investigation being reported	6
Objectives	3	State specific objectives, including any prespecified hypotheses	6
**Methods**			
Study design	4	Present key elements of study design early in the paper	7-9
Setting	5	Describe the setting, locations, and relevant dates, including periods of recruitment, exposure, follow-up, and data collection	7-9
Participants	6	(*a*) Give the eligibility criteria, and the sources and methods of selection of participants. Describe methods of follow-up	8
		(*b*) For matched studies, give matching criteria and number of exposed and unexposed	NA
Variables	7	Clearly define all outcomes, exposures, predictors, potential confounders, and effect modifiers. Give diagnostic criteria, if applicable	7-9
Data sources/ measurement	8*	For each variable of interest, give sources of data and details of methods of assessment (measurement). Describe comparability of assessment methods if there is more than one group	8-9
Bias	9	Describe any efforts to address potential sources of bias	15
Study size	10	Explain how the study size was arrived at	12
Quantitative variables	11	Explain how quantitative variables were handled in the analyses. If applicable, describe which groupings were chosen and why	8-9
Statistical methods	12	(*a*) Describe all statistical methods, including those used to control for confounding	9-10
		(*b*) Describe any methods used to examine subgroups and interactions	NA
		(*c*) Explain how missing data were addressed	9
		(*d*) If applicable, explain how loss to follow-up was addressed	NA
		(*e*) Describe any sensitivity analyses	NA
**Results**			

For peer review only

Participants	13*	(a) Report numbers of individuals at each stage of study—eg numbers potentially eligible, examined for eligibility, confirmed eligible, included in the study, completing follow-up, and analysed	10-12
		(b) Give reasons for non-participation at each stage	15
		(c) Consider use of a flow diagram	Not done
Descriptive data	14*	(a) Give characteristics of study participants (eg demographic, clinical, social) and information on exposures and potential confounders	15-18
		(b) Indicate number of participants with missing data for each variable of interest	In tables
		(c) Summarise follow-up time (eg, average and total amount)	NA
Outcome data	15*	Report numbers of outcome events or summary measures over time	Page 18<br>Table 1, Table 2
Main results	16	(*a*) Give unadjusted estimates and, if applicable, confounder-adjusted estimates and their precision (eg, 95% confidence interval). Make clear which confounders were adjusted for and why they were included	12-18<br>Table 1,2
		(*b*) Report category boundaries when continuous variables were categorized	NA
		(*c*) If relevant, consider translating estimates of relative risk into absolute risk for a meaningful time period	NA
Other analyses	17	Report other analyses done—eg analyses of subgroups and interactions, and sensitivity analyses	None performed
**Discussion**			
Key results	18	Summarise key results with reference to study objectives	22
**Limitations**			
Interpretation	20	Give a cautious overall interpretation of results considering objectives, limitations, multiplicity of analyses, results from similar studies, and other relevant evidence	22-23
Generalisability	21	Discuss the generalisability (external validity) of the study results	23
**Other information**			
Funding	22	Give the source of funding and the role of the funders for the present study and, if applicable, for the original study on which the present article is based	4

*Give information separately for cases and controls in case-control studies and, if applicable, for exposed and unexposed groups in cohort and cross-sectional studies.

Note: An Explanation and Elaboration article discusses each checklist item and gives methodological background and published examples of transparent reporting. The STROBE checklist is best used in conjunction with this article (freely available on the Web sites of PLoS Medicine at http://www.plosmedicine.org/, Annals of Internal Medicine at http://www.annals.org/, and Epidemiology at http://www.epidem.com/). Information on the STROBE Initiative is available at www.strobe-statement.org.

For peer review only
